# Expression profiling of *S. pombe *acetyltransferase mutants identifies redundant pathways of gene regulation

**DOI:** 10.1186/1471-2164-11-59

**Published:** 2010-01-22

**Authors:** Rebecca L Nugent, Anna Johnsson, Brian Fleharty, Madelaine Gogol, Yongtao Xue-Franzén, Chris Seidel, Anthony PH Wright, Susan L Forsburg

**Affiliations:** 1 Molecular and Computational Biology Section, University of Southern California, Los Angeles, California 90089-2910, USA; 2 Center for Biosciences, Department of Biosciences and Nutrition, Karolinska Institutet, S-141-57 Huddinge, Sweden and School of Life Sciences, Södertörn University, S-141 89 Huddinge, Sweden; 3 Stowers Institute for Medical Research, Kansas City, Missouri 64110, USA

## Abstract

**Background:**

Histone acetyltransferase enzymes (HATs) are implicated in regulation of transcription. HATs from different families may overlap in target and substrate specificity.

**Results:**

We isolated the *elp3*^+ ^gene encoding the histone acetyltransferase subunit of the Elongator complex in fission yeast and characterized the phenotype of an Δ*elp3 *mutant. We examined genetic interactions between Δ*elp3 *and two other HAT mutants, Δ*mst2 *and Δ*gcn5 *and used whole genome microarray analysis to analyze their effects on gene expression.

**Conclusions:**

Comparison of phenotypes and expression profiles in single, double and triple mutants indicate that these HAT enzymes have overlapping functions. Consistent with this, overlapping specificity in histone H3 acetylation is observed. However, there is no evidence for overlap with another HAT enzyme, encoded by the essential *mst1*^+ ^gene.

## Background

Chromatin is modulated in part by the pattern of different histone modifications, leading to the speculation that a "histone code" provides epigenetic information that facilitates chromosome activities [1,2]. Not surprisingly, the enzymes that modify chromatin have diverse roles in the cell that affect multiple DNA-dependent events. Acetylation was the first histone modification identified, and is associated with a variety of chromatin functions [2,3]. While some evidence suggests that acetylation changes association of DNA with the underlying nucleosomes [4], it also creates specific binding sites for proteins involved in a variety of DNA transactions [2,3]. Acetylation has long been linked to transcriptional activation, so that the histone acetyltransferase enzymes function generally as transcriptional activators [5].

Histone acetyltransferases are well conserved in eukaryotes and can be separated into multiple gene families based on primary sequence. The MYST family of histone acetyltransferases was named for founding members identified in yeast and humans, **M**OZ, **Y**BF2/SAS3, **S**AS2 and **T**IP60 [6]. Most species contain multiple members of this family. The Kat5 group, the most conserved family members, includes *Sp*Mst1 (*S. pombe*), *Sc*Esa1 (*S. cerevisiae*) and *h*Tip60 (human), which acetylates residues in the tails of histones H2A and H4 [7]. These proteins are the catalytic HATs for the NuA4 complex in their respective organisms [8,9]. In the yeasts the Kat5 orthologues are the only MYST family HATs that are essential for viability [10,11].

In budding yeast there are two other MYST family members, *Sc*Sas2 and *Sc*Sas3, which were originally isolated with defects in silencing the mating locus [12,13]. *Sc*Sas2 is the catalytic HAT for the budding yeast SAS complex that acetylates H4K16 [14]. It antagonizes the histone deacetylase Sir2 [15]. *Sc*Sas3 is the catalytic HAT for the NuA3 complex in budding yeast [16] which specifically acetylates histone H3 [17]. In *S. pombe *by contrast there is only one additional MYST family member, *Sp*Mst2 [11]. Mst2 shows sequence similarity to both *Sc*Sas2 and *Sc*Sas3. However, its biological function, in which it antagonizes *Sp*Sir2 in telomere silencing, suggests that it is a functional homologue of *Sc*Sas2 [11].

Another conserved family of HATs is the Gcn5-related N-acetyltransferase (GNAT) super-family consisting of Gcn5, Elp3, Hat1 and Hpa2, with Gcn5 (KAT2) and Elp3 (KAT9) as the most studied members [18]. The evolutionarily conserved Gcn5 is the catalytic subunit of several multi-subunit complexes including the SAGA co-activator complex [19]. Gcn5 acetylates multiple lysine residues of histones H3 and H2B and mediates both targeted and non-targeted (global) acetylation [20]. In *S. cerevisiae*, SAGA is recruited to promoter regions by transcription factors like *Sc*Gal4 and *Sc*Gcn4. Elongating RNA Pol II is associated with the Elongator complex that contains *Sc*Elp3 and has been shown to acetylate H3K14 and H4K8 in budding yeast [21]. Elp3 is highly conserved in structure and function between human and budding yeast [22].

While neither *ScGCN5 *nor *ScELP3 *is essential in budding yeast, a double mutation in *Δgcn5 Δelp3 *double mutant is significantly sicker than either single mutant [23], resulting in hypoacetylation of histone H3 in gene coding regions [24] and spreading of *Sc*Sir3 into sub-telomeric DNA [25]. Data from budding yeast suggest that *Sc*Gcn5 also overlaps with *Sc*Sas3, because a double deletion Δ*gcn5 *Δ*sas3 *is lethal [26]. This suggests that there are shared functions or targets of the NuA3, SAGA, and Elongator complexes in budding yeast, which implies that different HAT families may perform the same modifications. However, because the complement of HATs is somewhat different in *S. pombe*, it is not clear how general this overlap may be. *Sp*Gcn5 has been previously characterized for its effects on gene expression [27,28] and for chromatin binding genome-wide [29]. Surprisingly, relatively little change in gene expression profile is observed in *gcn5 *mutants despite a very high association with the chromatin. This suggests that *Sp*Gcn5 overlaps with other HATs.

Here, we examine evidence for redundancy between HAT enzymes in *S. pombe*. We report the initial characterization of fission yeast *elp3*^+ ^and examine phenotypes associated with single, double, and triple mutations of Δ*mst2*, Δ*gcn5 *and Δ*elp3*. We show that these enzymes affect expression of overlapping but nonidentical sets of genes, suggesting overlapping contributions to gene expression. Consistent with this, we observe biochemical evidence that these proteins share common histone substrates. We also observe that these phenotypes are distinct from the gene expression effects of a temperature sensitive allele of *mst1*, and suggest that multiple histone targets regulate gene activation in fission yeast.

## Results

**Characterization of Δ*elp3 *and interaction with other HAT mutants**.

**Figure 1:**
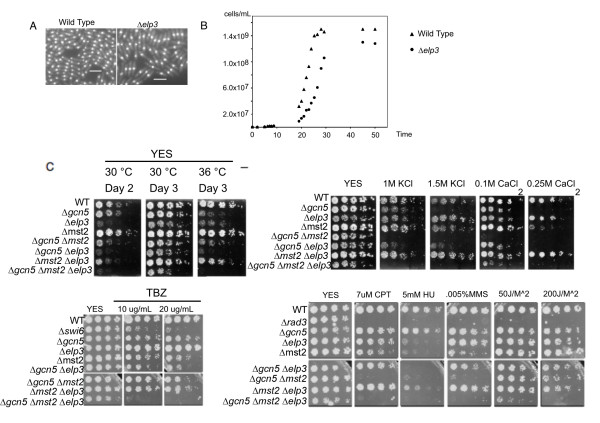
**Characterization of mutant phenotypes **. A: Δ*elp3 *morphology. Wild-type (FY368) and Δ*elp3 *(FY3851)cells were asynchronously grown at 32°C in YES. Cells were fixed and stained with DAPI. The scale bar represents 10 microns. B: Δ*elp3 *mutants have a growth lag. Representative growth curves for wild type (triangle) (FY368) and Δ*elp3 *(circle) (FY3851) cells in rich media (YES) at 30°C. The x-axis time scale indicates hours after inoculation that stared at a cell concentration of 4 × 10^5 ^cells/ml. The y-axis indicates number of cells/ml. C: Comparison of growth rates on YES at different temperatures and with different levels of salt. Cells were grown to exponential phase, serially diluted 5× and compared for growth on rich media after 2 or 3 days at 30°C. and 36°C, or onYES supplemented with indicated salt and grown for 2 to 4 days at 30°C. Strain list: wild-type (FY368), Δ*elp3*(FY3851), Δ*mst2 *(FY1890), Δ*gcn5 *Δ*mst2*(Hu990), Δ*gcn5 *Δ*elp3 *(FY3847), Δ*mst2 *Δ*elp3 *(FY3850), Δ*gcn5 *Δ*elp3 *Δ*mst2 *(3854) D: Comparison of drug sensitivity. Exponentially growing cells were diluted 5-fold on YES plates with the indicated drugs and grown 2-3 days at 32°C. Strain list: wild-type (FY261), Δ*swi6 *(FY1584), Δ*rad3 *(FY1104), Δ*elp3 *(FY 3851), Δ*gcn5 *(FY2943), Δ*mst2 *(FY1890), Δ*gcn5 *Δ*elp3 *(FY3847), Δ*gcn5 *Δ*mst2*(FY3361), Δ*mst2 *Δ*elp3 *(FY3850), Δ*gcn5 *Δ*elp3 *Δ*mst2 *(3854).

In order to compare the roles of different histone acetyltransferases on transcription in fission yeast, we first characterized the disruption of the histone acetyltransferase gene *elp3*^+^, the likely orthologue of the *ScELP3 *subunit of Elongator complex [21]. We found that *S. pombe elp3*^+ ^is a non-essential gene, as is its budding yeast orthologue [30]. However, Δ*elp3 *mutant cells have elongated morphology compared to wild type, indicative of cell cycle delay (Fig. 1A). The mean length of mononucleate cells is 6.7 μm in wild type, and 10.7 μm in Δ*elp3*. For binucleate cells, wild type averages 9.8 μm compared to 15.9 μm in Δ*elp3*. Thus Δ*elp3 *is approximately 60% larger than wild type. Also as in budding yeast [30], fission yeast Δ*elp3 *grows slowly due to a prolonged lag phase (Fig. 1B). Although the growth rate of Δ*elp3 *cells once they have reached exponential phase is similar to wild type, they appear to enter stationary phase at a lower cell density. Given the presumed role of Elp3 as part of the Elongator complex, we presume that this reflects defects in one or more growth-associated transcription program.

We examined other phenotypes associated with Δ*elp3*, and compared them to phenotypes associated with mutations in other non-essential histone acetyltransferases, Δ*mst2 *and Δ*gcn5*. We observed that Δ*elp3 *has poor growth overall compared to either Δ*gcn5 *or Δ*mst2 *(Fig. 1C). Both Δ*gcn5 *and Δ*elp3 *showed slight temperature sensitivity. As reported previously [28], Δ*gcn5 *is sensitive to salts (KCl or CaCl_2_), but we observed no overlap in this phenotype with Δ*mst2 *or Δ*elp3*. We also assayed the sensitivities of these HAT mutants to different DNA damaging agents (Fig. 1D), since histone acetylation is necessary for proper repair from DNA damage [31]. The *Δmst2 *mutants are sensitive to hydroxyurea, which depletes nucleotides and results in replication fork stalling, and to high doses of the DNA alkylating agent MMS, which causes DNA damage [11]. We observed that Δ*gcn5 *and Δ*mst2 *show similar sensitivity to hydroxyurea. Consistent with our previous results, Δ*mst2 *is modestly sensitive to low levels of MMS, but we observed no sensitivity in Δ*gcn5 *or Δ*elp3*. We also observed no sensitivity to the topoisomerase inhibitor camptothecin (which causes S phase specific DNA breaks) or UV irradiation. Δ*mst2 *is also sensitive to the microtubule poison thiabendazole (TBZ), a phenotype typical of mutations that affect centromere function or chromosome segregation.

We next constructed double and triple mutants to see whether there was evidence for epistasis or synthetic growth defects under these conditions. All combinations were viable, although the triple mutant Δ*gcn5 Δelp3 Δmst2 *was very slow-growing. The slow-growth phenotype of the triple mutant strain made it difficult to distinguish pathway specific effects from general sickness in this strain.

Previously, we assigned Mst2 as the likely *Sc*Sas2 orthologue, based on the similarity of their phenotypes, although it is also phylogenetically related to *Sc*Sas3. In budding yeast Δ*sas3 *Δ*gcn5 *is lethal [26], but in fission yeast Δ*mst2 *Δ*gcn5 *is viable. We assayed growth at 30° and 36°C for the single and double mutants. At 30°C, there was little effect observed. At 36°C, the Δ*gcn5 *Δ*mst2 *mutant displays a temperature sensitivity resembling Δ*gcn5*, indicating that there is no overlap between Gcn5 and Mst2 with regards to heat stress. The Δ*gcn5 *Δ*elp3 *mutant, however, shows increased temperature sensitivity compared to the single mutant strains. The Δ*mst2 *Δ*elp3 *mutant displayed a phenotype intermediate between the parents, suggesting that the faster growth associated with Δ*mst2 *in relation to wild type is also able to overcome the slow growth associated with Δ*elp3 *(Fig. 1C).

We examined the salt sensitivity of the double mutants, because Δ*gcn5 *has been associated with this phenotype [28]. The Δ*gcn5 *Δ*mst2 *double mutant is hypersensitive to salt compared to Δ*gcn5 *alone (Fig. 1C), suggesting that for this function, these two HATs overlap. In contrast, the Δ*gcn5 *Δ*elp3 *double mutant has a phenotype similar to Δ*gcn5 *alone suggesting that Elp3 does not contribute to proper salt stress response.

Since Δ*mst2 *has been linked to damage sensitivity, we also examined the response to different DNA damaging agents (Fig. 1D). Δ*mst2 *Δ*gcn5 *cells also showed increased sensitivity to MMS and CPT treatment relative to the two single mutants, suggesting that these two HAT enzymes make redundant contributions to replication-specific DNA damage. Interestingly, Δ*elp3 *modestly suppressed the sensitivity of Δ*mst2 *to HU (Fig. 1D). All the double mutants showed some sensitivity to TBZ; however, Δ*gcn5 *Δ*elp3 *was more sensitive than the parent strains.

### Genome wide expression studies

Previous studies showed while Gcn5 is bound to a high proportion of genes in *S. pombe *growing on rich media, it is requested for the expression of only a few of its binding targets [27,28]. This together with the synthetic and additive phenotypes observed here with the different mutant combinations suggests that HAT enzymes redundantly affect common pathways at the level of transcriptional regulation. However, it is also formally possible that they independently affect regulation of separate pathways that overlap downstream at the level of cellular function. To distinguish between these possibilities, we performed genome-wide analysis of expression using microarray technology to identify target genes that show HAT-specific changes in gene expression. We examined the effects on transcription upon deletion of the GNAT family HATs Gcn5 and Elp3 and the MYST HAT Mst2 both singly and in combination. We chose a very statistically rigorous cutoff of 3.25 log2 fold change for genes that we considered differentially expressed. This allowed us to be extremely confident that all results were biologically relevant and eliminated most false positives.

**Figure 2:**
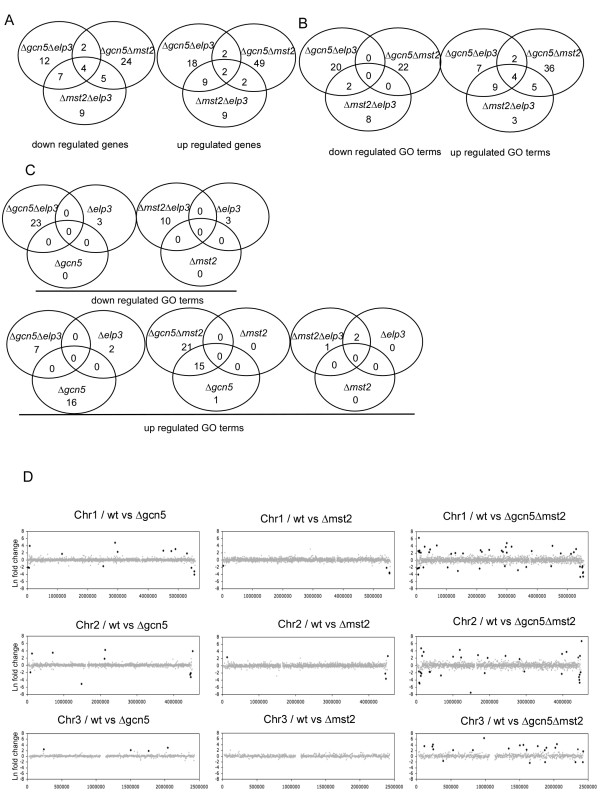
**Overlapping expression in different mutations**. A: Venn diagram of differentially down and up-regulated genes in double HAT mutants. (Additional File 5). B: Venn diagram of differentially down and up-regulated GO terms in the double HAT mutants using the affymetrix array. (Additional File 5) C: Venn diagram of differentially down and up-regulated GO terms of a double HAT mutant and the corresponding single HAT mutant. (Additional File 1). D: Localization of differentially regulated genes along chromosome 2 for the double mutant Δ*gcn5 *Δ*mst2 *(FY3361) and the corresponding single mutants, (Tables 1 and 2). Strains list: Δ*gcn5 *Δ*elp3 *(FY3847), Δ*gcn5 *Δ*mst2*(FY3361), Δ*mst2 *Δ*elp3 *(FY3850), Δ*gcn5 *(FY2943) and Δ*mst2 *(FY1890).

We found that there were very few genes whose expression was significantly changed by 3.25 log2 fold or more during asynchronous growth compared to wild-type cells in Δ*mst2*, Δ*gcn5 *or Δ*elp3 *cells (Table 1). Of the small number of genes with increased expression, the RecQ-type DNA helicase SPBCPT2R1.08c, located in the sub-telomere domain (*tlh2*^+^; [32,33]) was the only significantly up-regulated gene in all three mutant strains. Consistent with previously published data we found that there was an enrichment of genes involved in mating and meiosis in Δ*gcn5 *cells (Additional File 1). Interestingly, of the few genes with decreased expression in Δ*gcn5 *and Δ*mst2*, most are found within 150 kb of the ends of chromosome I or II, suggesting that cells require Gcn5 and Mst2 for expression of genes localized near the sub-telomeric region (Fig. 2D).

**Table 1:** Differentially regulated genes in single HAT mutants.

Down-regulated genes								
	Δgcn5			Δmst2			Δelp3	
Gene	Log change	p-value	Gene	Log change	p-value	Gene	Log change	p-value
his3	-5.12	7.16E-03	SPAC186.05c	-3.77	4.00E-07	SPAC186.05c	-4.31	1.11E-04
SPAC186.05c	-4.09	1.26E-07	SPBPB2B2.06c	-3.66	6.60E-07	SPAC186.06	-3.23	7.69E-03
gcn5	-3.47	1.08E-02	SPAC186.06	-3.52	1.94E-06	inv1	-2.57	4.30E-02
SPBPB2B2.06c	-3.26	3.11E-06	SPAC17G8.13c	-2.76	2.03E-02	str1	-2.50	5.79E-03
SPAC186.06	-3.23	5.82E-06	SPAC1039.02	-2.26	2.58E-03	SPBPB2B2.08	-2.34	3.98E-02
SPBPB2B2.01	-2.60	2.00E-05	SPBPB2B2.01	-2.23	1.12E-04	mei2	-2.27	2.18E-02
SPBPB2B2.05	-2.35	1.46E-02				ght4	-1.90	1.90E-02
SPAC977.14c	-2.29	2.54E-02				SPBC215.11c	-1.75	5.35E-05
SPBPB2B2.08	-2.22	2.01E-02						
SPAC1039.02	-2.17	3.46E-03						
SPAC977.05c	-2.10	2.09E-03						
SPBPB10D8.02c	-2.02	1.47E-03						
								
**Up-regulated genes**								
	**Δgcn5**			**Δmst2**			**Δelp3**	
**Gene**	**Log change**	**p-value**	**Gene**	**Log change**	**p-value**	**Gene**	**Log change**	**p-value**
spn6	2.05	4.71E-03	SPBCPT2R1.08c	2.08	3.59E-02	SPAPB1A11.01	1.88	1.01E-04
spk1	2.26	4.08E-03	SPBC359.06	2.29	2.33E-02	SPCC132.04c	2.01	6.01E-08
SPCC794.01c	2.34	3.09E-02				SPBC2G2.17c	2.02	2.69E-05
mam2	2.45	3.38E-03				SPBPB8B6.03	2.05	6.89E-04
mei2	2.57	5.66E-03				spo6	2.05	1.63E-04
SPCC1739.08c	2.94	2.22E-02				air2	2.08	6.21E-05
SPBC359.06	3.20	2.79E-03				SPAC750.04c	2.08	3.73E-06
mfm1	3.23	4.03E-03				spn5	2.17	9.14E-06
mfm3	3.27	3.68E-03				ctr4	2.22	1.49E-03
ght3	3.87	8.30E-03				SPAC2E1P3.02c	2.62	5.60E-08
SPBCPT2R1.08c	3.89	7.73E-04				SPAC750.07c	3.83	2.13E-03
matmc_1	4.18	8.90E-03				dak2	3.86	2.68E-02
mfm2	4.73	8.99E-03				SPBCPT2R1.08c	4.50	1.23E-03

This table lists the down and up-regulated genes of mutant HATs compared to wild-type cells using an Affymetrix microarray.

**Figure 3:**
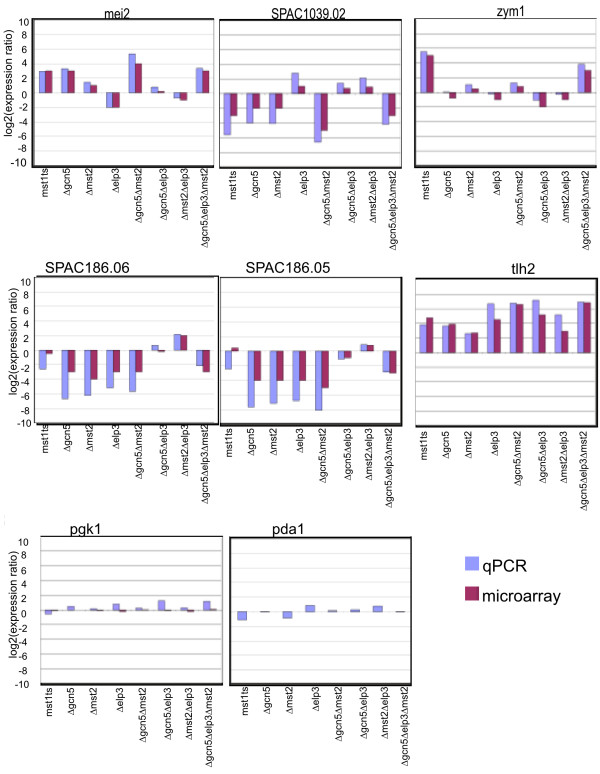
**Validation of microarray results by qPCR**. Genes that showed differential expression from microarray analysis were analyzed in different HAT mutants through qualitative PCR (qPCR). The log2 fold expression ratios from microarray and qPCR experiments for the mutants versus wild type were plotted. qPCR results are indicated by the blue bars, microarray by the purple bars. Strain list: Strain list: Δ*elp3 *(FY 3851), Δ*gcn5 *(FY2943), Δ*mst2 *(FY1890), Δ*gcn5 *Δ*elp3 *(FY3847), Δ*gcn5 *Δ*mst2*(FY3361), Δ*mst2 *Δ*elp3 *(FY3850), Δ*gcn5 *Δ*elp3 *Δ*mst2 *(3854).

The few genes that were differentially expressed in Δ*elp3 *and Δ*gcn5 *showed significant over-representation in several functional ontology groups. In agreement with prior reports [27] we observed that Δ*gcn5 *mutants de-repressed mating and meiosis genes. Specifically we found that gene ontology (GO) terms [34] related to mating and meiosis were found in the up-regulated genes, indicating that Gcn5 is required for repression of these genes. In Δ*elp3 *mutants we found that genes significantly enriched in GO classes associated with trans-membrane transport were down-regulated and those associated with ion transport were up-regulated (Fig 2, Additional File 1). Interestingly, *mei2*, which has been shown to be repressed by Gcn5 [27] was down-regulated in Δ*elp3 *(Fig. 3). This may indicate that Gcn5 and Elp3 target the same pathway but with opposite effects. The few genes that were differentially expressed in Δ*mst2 *mutants provided no over-represented GO classes.

### Global gene expression changes in double mutants

We next asked whether the transcription profiles of the double mutants were simply additive to the single mutants, or whether they affected new genes not in common to the single mutant strains. We assayed changes in gene expression in the double mutants. The number of differentially expressed genes in the double and triple mutants was significantly increased compared to the respective single mutants for each strain tested (Fig. 2, Additional File 1, Additional File 2), suggesting overlapping regulation rather than an additive effect.

Only the helicase *tlh2*^+ ^and a metabolic kinase, *dak1*^+ ^were significantly up-regulated in all mutant combinations. The double mutants Δ*gcn5 *Δ*elp3 *and Δ*mst2 *Δ*elp3 *had the most overlapping up-regulated genes (Fig. 2, Additional File 3). These genes included metal transporters and the sporulation specific gene *spo6*^+^. We found that Δ*gcn5 *Δ*mst2 *cells had a greater number of up-regulated genes related to the mating pathway than Δ*gcn5 *alone, suggesting that Gcn5 and Mst2 are functionally redundant in repressing sexual differentiation. This is also observed, although to a lesser extent, with the Δ*gcn5 *Δ*elp3 *mutant. However changes associated with Δ*mst2 *Δ*elp3 *were not significantly enriched in gene classes associated with mating or meiosis, except for *spo6*^+^, which is essential for progression of meiosis II and sporulation. When we examined down-regulated genes in the double mutants, we again found the most overlap between Δ*gcn5 *Δ*elp3 *and Δ*mst2 *Δ*elp3 *cells, however there were more differentially expressed genes in Δ*gcn5 *Δ*mst2*. A large fraction of these genes were found to be sub-telomeric (Fig. 2D) which suggests that Mst2 and Gcn5 but not Elp3 function preferentially in the sub-telomeric regions. This is consistent with previous studies suggesting that Mst2 works at the telomere [11].

### Functional redundancies between HATs

To determine whether the mutations affected distinct functional groups, we categorized the affected gene targets by gene ontology (GO) classification. As was apparent for the individual genes, the genes affected by the double mutants were more significantly enriched in a larger number of GO classes than for the single mutants. For GO classes enriched in down-regulated genes there were 23 terms for Δ*gcn5 *Δ*elp3*, 22 for Δ*gcn5 *Δ*mst2*, while Δ*mst2 *Δ*elp3 *had 10 enriched GO terms (Fig 2, Additional File 1). Curiously there is almost no overlap in enriched GO terms associated with genes down-regulated between double mutants, with the exception of cellular response to stress and response to stimulus shared between *mst2 *Δ*elp3 *and Δ*gcn5 *Δ*elp3*. Therefore we conclude that Gcn5, Mst2 and Elp3 are functionally redundant in activating transcription. For GO categories enriched with up-regulated genes there were 7 categories for Δ*gcn5 *Δ*elp3*, 36 for Δ*gcn5 *Δ*mst2*, and Δ*mst2 *Δ*elp3 *had 3 enriched GO terms (Fig. 2). There are no overlapping enriched up-regulated GO categories between double mutants. When we examined the number of genes and the GO terms they represent in the double mutants compared to the single mutants, we conclude that Gcn5, Mst2 and Elp3 are functionally redundant in repressing transcription because the number of terms enriched in the double mutants cannot be explained by adding the single mutants' terms together. There were more significantly enriched genes in the double mutants than in the single mutants.

### Validation of microarray results

We used quantitative PCR (qPCR) to confirm the microarray results. The ddCt method [35] was applied to measure relative fold differences between each strain and one wild type sample with *act1*^+ ^used as the loading control. For each strain, we chose genes that were predicted by the microarray to have significant changes in transcription. The fold changes of the other two wild type samples relative to the first wild type sample was small (2-fold at the most) across all the loci. The fold changes of the other two control genes relative *to act1*^+ ^were also small (2-fold at the most) across all the strains. We found high degrees of congruence in gene expression as measured by RT-PCR and microarray analysis. (Fig 3).

### Salt response in Δ*mst2 *and Δ*gcn5 *mutants

Since Δ*mst2 *and Δ*gcn5 *showed a synthetic phenotype in salt sensitivity, we performed expression profiling on Δ*mst2 *and Δ*gcn5*Δ*mst2 *double mutant under KCl treatment and compared this to our previous results on Δ*gcn5 *using the same platform [28]. The number of affected genes in Δ*mst2 *increases during KCl induced stress but fewer genes are affected in Δ*mst2 *compared to Δ*gcn5*, consistent with the observation that Δ*mst2 *alone has little effect on salt response. To find genes that require Gcn5 or Mst2 during KCl stress we compared the expression levels between Δ*gcn5 *and Δ*gcn5 *Δ*mst2*.

**Figure 4:**
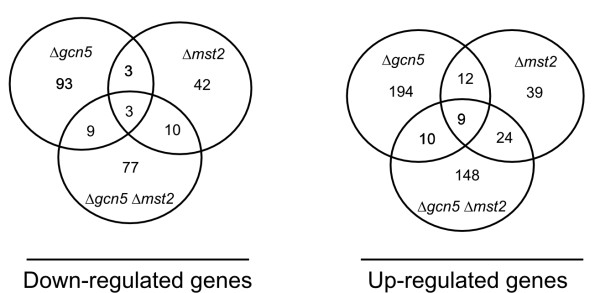
**Salt response is redundantly regulated by multiple HAT families**. Venn diagram of differentially down and up-regulated genes after salt stress in Δ*gcn5*, Δ*mst2 *and Δ*gcn5 *Δ*mst2 *HAT mutants as determined by the Eurogenetech array platform (Additional File 3). Strains Δ*gcn5 *(Hu799), Δ*mst2 *(1890) Δ*gcn5 *Δ*mst2*(Hu990).

We found 99 genes that were significantly down-regulated and 191 genes significantly up-regulated in the double mutant, compared to Δ*gcn5 *single mutant (Additional File 4). About 80% of these genes were not significantly regulated in either of the single mutants (Fig 4). This suggests that the two HAT enzymes overlap in the regulation of salt-responsive pathways, although there was no significant overlap in particular GO pathways.

### Histone acetylation levels in HAT mutants

Gcn5/SAGA is a known regulator of H3 acetylation, as is Elp3/Elongator [21,36]. The phenotypes associated with Δ*mst2 *suggest it is the functional orthologue of *Sc*Sas2, but if this is the case, there is no obvious NuA3/Sas3 homologue in *S. pombe *to overlap with Gcn5 activity, as Sas3 does in *S. cerevisiae*. Because more genes showed changes in expression in the double mutants than would be expected by a simple sum of those affected in the single mutants, we examined levels of histone H3 acetylation in the mutant strains to see whether there is evidence for overlapping specificity at the level of global histone acetylation.

**Figure 5:**
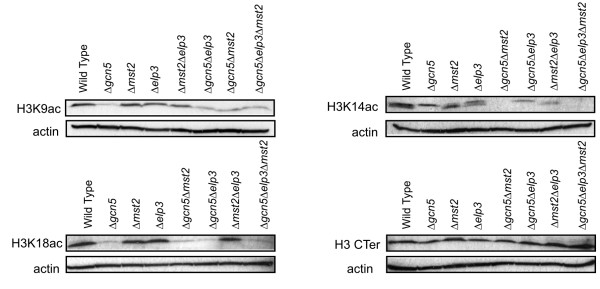
**Histone H3 acetylation is differentially affected by different HAT mutants**. Levels were determined by Western blots of whole cell lysates from the indicated strains with antibodies specific to H3K9ac, H3K14ac, H3K18ac, compared to levels of actin and total histone H3 (C-term). Strain list: wild-type (FY368), Δ*gcn5 *(Hu799), Δ*elp3*(FY3851), Δ*mst2 *(FY1890), Δ*gcn5 *Δ*mst2*(Hu990), Δ*gcn5 *Δ*elp3 *(FY3847), Δ*mst2 *Δ*elp3 *(FY3850), Δ*gcn5 *Δ*elp3 *Δ*mst2 *(3854)

As expected, we found H3 acetylation levels on H3K9, K14 and K18 were dramatically reduced in the Δ*gcn5 *mutant (Fig 5). In Δ*elp3 *and Δ*mst2 *as well as in the Δ*mst2 *Δ*elp3 *double mutant there is only a modest decrease in H3K9 acetylation levels compared to wild type. In contrast, all strains lacking *gcn5 *show significant loss of H3K9ac (Fig. 5A), suggesting that Gcn5 is the major contributor to H3K9 acetylation. Similar results were observed for H3K18ac.

For H3K14 acetylation we found significant reductions of acetylation levels for each single mutant compared to wild-type cells. Strikingly, in the double mutant Δ*gcn5 *Δ*mst2*, the signal is much more strongly reduced (Fig. 5B). None of the deletion mutants showed any significant reduction in the overall level of histone H3 (Fig. 5D). Therefore we conclude that Gcn5 (GNAT family) and Mst2 (MYST family) each contribute to acetylate H3K14, which has been shown to be important for the response to salt stress [37]. Defective acetylation of H3K14 could explain the hypersensitivity to salt stress observed in the Δ*gcn5 *Δ*mst2 *mutant.

### Genes differentially expressed by the essential HAT Mst1

**Figure 6:**
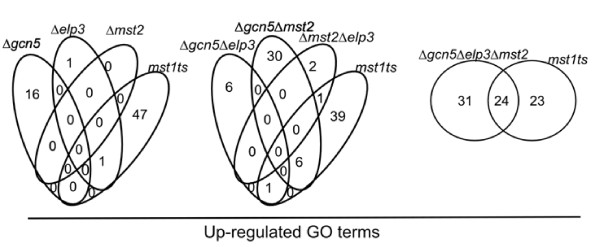
**Venn diagram of differentially down and up-regulated GO terms of *mst1*^*ts *^mutant compared to single, double, and triple HAT mutants**. (Additional File 1) Strain list: Strain list: wild-type (FY261), *mst1*^*ts *^(FY2535) Δ*elp3 (*FY 3851), Δ*gcn5 *(FY2943), Δ*mst2 *(FY1890), Δ*gcn5 *Δ*elp3 *(FY3847), Δ*gcn5 *Δ*mst2*(FY3361), Δ*mst2 *Δ*elp3 *(FY3850), Δ*gcn5 *Δ*elp3 *Δ*mst2 *(3854).

Finally, we examined the essential MYST family HAT Mst1 using the temperature sensitive allele *mst1*^*ts*^[38]. The *mst1 *mutant was grown at the semi-permissive temperature 30°C overnight and RNA was isolated for microarray analysis. There were many more differentially expressed genes in the *mst1 *mutant compared to any of the non-essential HATs studied here (Table 2). There were also many more up-regulated than down-regulated genes in the *mst1 *mutant, suggesting that it functions in repression of gene expression. Consistent with this there were no GO categories that were significantly enriched in the down-regulated genes, however there were many that were enriched in the up-regulated genes (Additional File 1). These GO groups include metal and salt transporters, and other metabolic processes. When we compared these GO categories with those observed for the other mutants, we found that there was some overlap with the double mutant Δ*gcn5 *Δ*mst2 *and the triple mutant Δ*gcn5 *Δ*elp3 *Δ*mst2*, suggesting general stress responses in these mutantsare de-regulated (Fig 6).

**Table 2:** Differentially expressed genes in *mst1*^*ts*^

Down-regulated genes								
	mst^1ts^							
Gene	Log2 change	p-value						
SPAC1039.02	-2.66	0.000635869						
SPBC29A3.05	-2.18	5.55E-05						
SPCC757.06	-2.08	1.87E-07						
								
**Up-regulated genes**								
	**mst1^ts^**			**mst1^ts^**			**mst1^*ts*^**	
**Gene**	**Log 2 change**	**p-value**	**Gene**	**Log 2 change**	**p-value**	**Gene**	**Log2 change**	**p-value**
SPBC1348.09	1.89	3.69E-11	SPAC977.17	2.40	1.80E-08	map2	3.52	2.50E-12
SPAC22F8.05	1.90	0.001560473	SPBC1348.14c	2.41	2.14E-15	SPAPJ695.01c	3.52	2.00E-09
SPAC3C7.13c	1.92	3.23E-10	SPAC5H10.02c	2.46	3.89E-11	SPBC1198.01	3.55	7.19E-16
SPBPB2B2.06c	1.93	0.000987078	SPCC663.08c	2.54	0.009807792	SPAC22A12.17c	3.57	1.96E-11
SPBPB2B2.07c	1.93	9.41E-10	SPBC725.10	2.57	9.52E-05	SPAPB1A11.03	3.59	1.50E-13
nta1	1.94	3.20E-06	SPBC83.19c	2.58	3.49E-12	SPAC1F8.04c	3.59	5.41E-07
SPCC191.01	1.94	5.61E-06	SPCC1393.12	2.58	4.35E-11	SPAC11D3.01c	3.61	1.72E-07
SPCC70.04c	1.95	2.09E-06	SPBPB2B2.08	2.60	0.007868315	SPBPB8B6.03	3.64	2.07E-12
meu7	1.96	2.81E-05	SPCC777.04	2.63	3.77E-05	SPBP4G3.03	3.65	2.74E-13
SPAC9.10	1.98	8.57E-09	SPAC26F1.05	2.64	1.00E-07	SPCC663.06c	3.66	3.28E-05
map1	1.98	0.000618909	pma2	2.64	6.19E-07	SPBPB21E7.04c	3.67	3.08E-06
SPBC8E4.05c	1.99	1.54E-08	SPAC32A11.02c	2.65	4.69E-09	frp1	3.69	3.37E-05
SPAC637.03	2.03	0.000132799	SPBC2G2.17c	2.66	2.11E-07	SPCC737.04	3.85	3.10E-07
SPBC4C3.08	2.04	6.23E-08	SPAC56F8.15	2.68	0.001276426	SPBC359.06	3.91	0.000499938
meu10	2.05	4.69E-07	SPAPJ691.02	2.72	1.52E-05	apc10	3.96	4.49E-12
SPCC1840.12	2.05	4.17E-06	SPBC1773.06c	2.76	0.000469242	SPAPB8E5.10	3.98	1.31E-12
SPBPB2B2.02	2.06	0.000305555	SPBC1685.05	2.86	1.62E-11	SPBC16E9.16c	4.00	6.07E-13
SPBC19C7.04c	2.09	0.001283597	SPAC4F10.17	2.88	6.57E-09	SPAC29A4.12c	4.06	1.45E-09
SPAC5H10.04	2.10	1.33E-06	SPCC16A11.15c	2.94	5.09E-11	SPAC869.08	4.08	1.01E-15
SPBC947.05c	2.11	5.62E-08	SPCC1739.08c	2.96	0.021512753	SPBC3H7.08c	4.09	9.33E-14
SPAC3H8.09c	2.11	1.77E-10	SPAC23C11.06c	2.99	1.15E-07	SPBCPT2R1.08c	4.16	0.000258571
ish1	2.11	9.95E-06	SPAC15E1.02c	3.00	8.68E-09	fbp1	4.32	3.57E-14
SPAC513.02	2.12	1.94E-06	SPBC1105.13c	3.02	1.10E-07	SPBC56F2.06	4.36	2.99E-07
SPAC4H3.03c	2.14	0.000486057	grt1	3.04	7.61E-08	mel1	4.43	2.62E-15
SPAPB15E9.02c	2.17	3.00E-08	SPCC338.18	3.04	7.26E-07	SPAC139.05	4.49	1.53E-09
SPCC794.01c	2.18	0.042343035	SPBPB2B2.18	3.05	1.46E-05	SPCPB16A4.06c	4.53	1.21E-11
SPAC1F8.02c	2.18	3.57E-07	SPCC1322.07c	3.10	9.51E-09	SPBC1289.14	4.61	8.12E-16
SPBC4.01	2.20	9.35E-05	SPCC757.03c	3.11	2.10E-09	ght3	4.72	0.001973142
SPBPB8B6.02c	2.23	1.43E-12	SPAC4H3.08	3.16	4.98E-12	zym1	4.85	1.83E-08
SPBC36.02c	2.23	0.000135759	SPBPB2B2.12c	3.16	0.00706426	SPBC24C6.09c	4.94	1.55E-13
SPCC1393.07c	2.23	4.88E-10	spk1	3.17	0.000216514	dak2	5.02	4.45E-12
SPAC513.06c	2.24	1.52E-09	mei2	3.25	0.000888516	SPAC186.02c	5.11	6.43E-15
SPCPB1C11.02	2.25	7.04E-07	SPAC977.05c	3.29	2.46E-05	eno102	5.11	3.01E-13
ste11	2.27	0.000335259	SPAC1F8.08	3.32	2.20E-09	SPAC3G9.11c	5.13	5.88E-11
wtf20	2.28	4.37E-06	SPAC3G6.07	3.33	1.53E-05	SPAC22G7.11c	5.22	4.34E-14
ppk33	2.28	0.000119009	SPACUNK4.17	3.33	3.64E-07	SPBC23G7.10c	5.25	4.94E-06
SPAC1F7.06	2.33	5.44E-12	SPAC23H3.15c	3.42	1.03E-05	isp3	5.36	1.87E-11
ssa1	2.34	0.000834021	caf5	3.44	4.99E-07	SPAC869.06c	6.61	2.79E-13
cta3	2.36	6.88E-11	str3	3.48	2.47E-05	SPAC869.09	6.68	2.91E-12
agl1	2.37	0.026304809						

This table lists the down and up-regulated genes of *mst1*^*ts *^cells compared to wild-type cells using an Affymetrix microarray.

## Discussion

Fission yeast shares several conserved histone acetyltransferases with other eukaryotes. In the GNAT family, the Elp3 (KAT9) and Gcn5 (KAT2) enzymes are highly conserved [7]. In the MYST family, the Kat5 HAT Esa1/Tip60/Mst1 protein is also highly conserved. However, the relationships between the other MYST family members are less clear-cut. In budding yeast, two additional MYST proteins *Sc*Sas2 and *Sc*Sas3 have been characterized. *Sc*Sas2 antagonizes *Sc*Sir2 in telomere silencing as a component of the SAS complex. *Sc*Sas3, a component of NuA3 [16], overlaps with Gcn5 such that a double mutant *Sc gcn5 sas3 *is lethal [26]. By contrast, there is only one additional MYST protein in *S. pombe*, Mst2, which resembles both *S. cerevisiae *proteins in primary sequence. Like *Sc*Sas2, *Sp*Mst2 antagonizes *Sp*Sir2 in telomere silencing [11], while work from this study suggests that like *Sc*Sas3, *Sp*Mst2 acetylates histone H3K14. This works shows that *Sp*Mst2 functions similarly to both *Sc*Sas2 and *Sc*Sas3. To dissect out the relationship between these potentially overlapping HAT enzymes Mst2, Elp3 and Gcn5, we compared their phenotypic and transcriptional interactions in *S. pombe*.

First, we performed the initial characterization of the catalytic HAT, Elp3, which is the likely catalytic subunit of the Elongator complex in *S. pombe*. The strain *elp3 *is viable, although it suffers defects in overall cell growth. It shows a delay in entry into the cell cycle, and premature cell cycle exit (Fig. 1B). It should be noted that some of the phenotypes seen in Elp3 defective strains could result from defects in other functions that have been reported for the Elongator complex in addition to its role as a HAT [39,40]. We next investigated whether Δ*elp3 *shows genetic interactions with mutations in other non-essential HAT genes encoded by *gcn5*^+ ^and *mst2*^+^. Double and triple mutants are all viable, although they show increasingly severe growth defects as more HAT genes are mutated. The viability of *Sp*Δ*mst2 *Δ*gcn5 *contrasts with the lethality of *Sc*Δ*sas3 *Δ*gcn5 *and suggests that Mst2 is not a simple equivalent of *Sc*Sas3. Alternatively, there may be another enzyme in fission yeast, which provides an additional degree of redundancy. We suggest that Mst2 fulfills many of the functions associated with both *Sc*Sas2 and *Sc*Sas3 (see below).

Phenotypic analysis suggests distinct effects of each HAT. For example, as shown previously Δ*gcn5 *mutants are de-repressed for mating and meiosis functions, and are sensitive to certain salt-stress conditions [27,28]. Δ*mst2 *mutants show modest sensitivity to DNA damaging agents such as HU and MMS. Δ*gcn5 *Δ*mst2 *double mutants show significantly increased salt sensitivity, and a modest increase in DNA damage sensitivity relative to single mutants. Thus, we conclude that in response to salt stress, Gcn5 and Mst2 have a substantial functional overlap. In contrast, Δ*gcn5 *Δ*elp3 *and Δ*mst2 *Δ*elp3 *showed less evidence for synthetic phenotypes, with only a modest increase in TBZ sensitivity in Δ*gcn5 *Δ*elp3 *relative to the parents (Fig. 1C &1D). In fact, Elp3 appears to be antagonistic to Mst2, because Δ*elp3 *suppresses Δ*mst2 *mutant sensitivity to high salt concentrations and HU. We speculate this could be due to slowing down the growth of Δ*mst2 *cells, allowing time for the cells to repair any damage that may have occurred. Together, these data suggest that Gcn5 affects multiple pathways, some overlapping with Elp3, and others overlapping with Mst2. However, since the double mutants and even the triple mutants are viable, either additional HAT enzymes play a role, or the acetylation modifications controlled by these enzymes are not strictly essential for viability.

To determine whether the functional redundancy between HATs is at the level of gene expression, we performed transcriptional profiling using DNA microarrays of Δ*elp3*, Δ*gcn5*, and Δ*mst2*. Similar to previous results with Δ*gcn5 *[27,28] we found that few genes were affected by deletion of any single HAT gene under favorable growth conditions. Although HATs are presumed to be gene activators that facilitate transcriptional initiation or elongation [5], we found that gene expression was as likely to be increased as reduced in our mutant strains. Up-regulated genes could represent indirect targets, although many Gcn5-repressed genes are known to be bound by Gcn5 [37]. This suggests that Gcn5 may play a direct role in gene repression, which has been described previously [27]. It is thus possible that the other HATs studied here also play direct roles in transcriptional repression.

When we examined the double mutants, we found that their gene expression profiles were not simply the combination of the profiles from the two single mutants, but affected additional genes. This supports the hypothesis that the HATs play partly redundant roles in gene regulation, with multiple enzymes contributing to expression of common targets. Importantly, although they may affect common genes, the regulatory mechanisms may be distinct. In budding yeast, although *Sc*Gcn5 and *Sc*Elp3 affect transcription of the *Sc*Hsp70 genes Sc*SSA3 *and Sc*SSA4*, this occurs by different mechanisms because Gcn5 is required for transcription factor binding while Elp3 is required for proper Pol II elongation [41]. However, we have shown that in fission yeast *Sp*Gcn5 also has a role in transcriptional elongation [37] which could suggest a more direct mechanistic overlap for *Sp*Gcn5 and *Sp*Elp3 in this species.

Only one gene was significantly increased in all strains: the RecQ-type DNA helicase SPBCPT2R1.08c, located in the sub-telomere domain (Tlh2; [32,33]). We showed previously that Δ*mst2 *increases silencing at the telomere and in telomere associated regions, consistent with a role for Mst2 in antagonizing Sir2. Loss of Mst2 results in a loss of overall H4 acetylation [11] and H3K9 acetylation in Δ*mst2 *in a very limited region adjacent to the telomere 18 kb from the end. Interestingly, this region is telomere-distal to the *tlh2 *helicase gene located 13 kb from the end of the telomere chromosome II, which is highly up-regulated in the mutant. In contrast, genes at 27.5, 29 and 31 kb away from the telomere are down-regulated in Δ*mst2 *(SPAC186.06, SPAC186.05 and SPBCPT2R1.02 respectively), while at 47 kb telomere distal (SPAC869.01), there is no change in expression levels. This suggests a gradient effect on transcription of genes in these regions.

The few genes that are down-regulated in Δ*gcn5 *are localized near the ends of chromosome I and II which suggest that the repression might be due to spreading of heterochromatin by loss of H3K9ac. The combined effect of the double mutant may indicate a combination of effects associated with loss of both histone H3K9 and H3K14 acetylation. Clearly, there are specific regions at the telomeres that are regulated by specific HATs but more work needs to be done to fully understand the boundaries of the telomeres and the regulation between the highly heterochromatic region and the rest of the chromosome.

We used gene ontology (GO) classification to define the functional roles of the few genes that were differentially expressed in single mutants. We found that those in Δ*elp3 *and Δ*gcn5*, but not Δ*mst2 *associated with a few distinct classes. Consistent with previous results [27], Δ*gcn5 *mutants showed increased expression of specific sexual differentiation genes as well as enrichment for GO terms [34] related to mating and meiosis.

Gcn5 represses transcription of *ste11*^+ ^[27], which is an important regulator of the mating pathway in response to nutrient limitation. Although we find very similar functional groups that are positively regulated in the absence of Δ*gcn5*, we did not see evidence for induction of *ste11 *above our threshold level (Table 1).

Consistent with prior work, our study also found that *mei2*^+ ^was up-regulated in Δ*gcn5 *[27]; this was also seen for *mst1*^*ts *^and to a lesser extent Δ*mst2 *(Fig 3), indicating multiple inputs into this essential regulator of sexual development. The Δ*gcn5 *Δ*mst2 *mutant increased *mei2*^+ ^and *ste11+ *mRNA levels above those observed in the single mutants, suggesting these two HATs function redundantly in repression. It is possible that they might also have non-histone targets in common since the repression of *mei2*^+ ^expression by Gcn5 was suggested to be mediated by a histone independent mechanism [27].

In contrast, deletion of Δ*elp3 *results in loss of *mei2*^+ ^transcription and in the Δ*gcn5 *Δ*elp3 *and Δ*mst2 *Δ*elp3 *double mutants there is also a reduction of *mei2*^+ ^transcription. Even the triple mutant shows lower levels of *mei2*^+ ^than the Δ*gcn5 *Δ*mst2 *double mutant, suggesting that Δ*elp3 *has an opposite effect on *mei2*^+ ^transcription than Δ*gcn5*. Interestingly transcription of SPAC1039.02, a putative phosphoesterase, shows a similar, but opposite, regulation pattern as it is down-regulated in Δ*gcn5*, *mst1*^*ts *^and Δ*mst2 *while being up-regulated in Δ*elp3*. Thus, for some targets, Gcn5 and Mst2 appear to overlap, while Elp3 appears to be antagonistic. This finding suggests that the common model linking HAT enzymes with gene activation is to simplistic. Dissecting the contribution of each HAT to gene expression or other effects will require future molecular studies mapping the histone acetylation and physical binding of HATs at different gene regions or their association with the transcription machinery.

To determine the molecular basis for the overlap of HAT targets, we examined histone H3 acetylation. Three acetylation sites on histone H3 are associated with gene expression: K9, K14, and K18 [42]. H3K9 is acetylated in euchromatin, but methylated in heterochromatin; consistent with other species, our data indicate that Gcn5 is the primary contributor to H3K9Ac because acetylation of this residue is strongly reduced in Δ*gcn5 *but relatively unperturbed by the other HAT mutations.

H3K14 acetylation is also associated with gene expression [42]. Mutations that change the H3K14 residue display hypersensitivity to KCl and CaCl_2 _induced stress in Δ*gcn5 *cells [37]. Little or no H3K14 acetylation is observed in the Δ*gcn5 *Δ*mst2 *double mutant. Consistent with this, we observed that Δ*gcn5 *Δ*mst2 *is significantly more salt-sensitive than either single mutation. We conclude that these two HATs both contribute to H3K14 acetylation in response to stress. Importantly, this acetylation is not essential for viability in *S. pombe*. Data from budding yeast also indicates an overlap between *Sc*Sas3 and *Sc*Gcn5 in H3K14 acetylation [26]. Interestingly, the budding yeast data also suggest that Δ*sas3 *is not lethal in combination with disruption of other SAGA subunits that are essential for Gcn5 activity against histones, and deletion of histone N-terminal H3 tails entirely is not lethal in budding yeast [26]. Thus, the essential redundant function of these enzymes may not be in histone modification. It is now well-established that HAT proteins acetylate substrates other than histones [43]. Perhaps it is one such substrate that relies on Gcn5 or Sas3 in budding yeast, but on a different enzyme in fission yeast. These results indicate that Mst2 has functions in common with both budding yeast enzymes.

Finally, we found that as expected there was little overlap between expression profiles in the non-essential HAT mutant strains, and cells with a temperature sensitive mutation of the essential HAT *mst1*^+^. Mst1 is known to be required for DNA damage response and chromosome segregation [38]. However, we did not see a significant increase in GO terms related to these functions, which is consistent with data suggesting its effects are not mediated through the transcriptional program [44]. There was a significant overlap of regulated genes between Mst1 and the triple mutant Δ*gcn5 *Δ*mst2 *Δ*elp3*, as half of the genes regulated in the triple mutant were also regulated in *mst1*^*ts*^. When we compared the regulated GO terms from *mst1*^*ts *^and Δ*gcn5 *Δ*elp3 *Δ*mst2 *we found that these mutants shared terms related to metal and ion homeostasis, metal transports as well as regulation of meiosis. Since both strains show severe growth defects under the conditions employed, we suggest that these overlaps may represent the effects of general cellular stress rather than a particular transcriptional program.

## Conclusions

We isolated and characterized the putative Elongator HAT Elp3, showing Δ*elp3 *cells are delayed in the cell cycle relative to wild-type cells, and then compared it to other HAT mutants. Our study suggests, through analysis of single, double and triple mutants of MYST and GNAT family HATs, that histone acetyltransferases play partially redundant role in gene regulation, and that their role can be positive, or negative for the transcription of genes. We found evidence for significant redundancy between Mst2 and Gcn5 in regulation of stress response, and response to DNA damage. We found that different HAT families can share specificity for a common lysine residue in histone H3, thereby contributing to the modulation of gene expression. For example, we found that Gcn5 and Mst2 both acetylate histone H3 lysine 14. This work highlights the functional redundancies of different HAT families on transcription, salt response, and DNA damage repair. Future work will be required to investigate the direct contributions of these HATs to individual genes, as opposed to selected regulation of other transcriptional master regulators.

## Methods

### Strains, media, and manipulations

Strains used in this study are listed in Table 3. Strains were grown and maintained on yeast extract plus supplements (YES) or Edinburgh minimal media (EMM) with appropriate supplements, using standard techniques [45]. Matings were performed on synthetic sporulation agar (SPAS) plates for 2-3 days at 25°C. Double mutants were constructed by standard tetrad analysis or random spore analysis. G418 plates were YES supplemented with 100 μg/ml G418 (Sigma).

**Table 3:** Strains used in this study

Strain	Genotype	
FY261	h+ can1-1 leu1-32 ade6-M216 ura4-D18	SLF stock
FY368	h-, leu1-32, ura4-D18, ade6-M210	AW stock
FY1104	h- Δrad3::ura4+ ura4 leu1-32 ade6-M210 (?)	SLF stock
FY1584	h+ Δswi6::his1+ ade6-M210 leu1-32 ura4-DS/E his1-102	R. Allshire
FY1890	h+ Δmst2::ura4+ ura4-D18 ade6-M210 leu1-32	Gomez EB 2005
FY2535	h+ Δmst1::kanMX6 leu1::nmt-mst1L-S-leu1+ ade6-M210 ura4-D18	Gomez EB 2008
FY2943	h- Δgcn5::kanMX ura4-D18 leu1-32 ade6-M21? his3-D1	SLF stock
FY3361	h+ Δgcn5::Kanmx6 Δmst2::ura4+ Ura4D18 ade6 m21? leu1-32 his3D1	this work
FY3847	h+ Δgcn5::KanMX4 Δelp3::KanMX6 leu1-32 ura4-D18 ade6-M210	this work
FY3850	h+ Δelp3::KanMX6 Δmst2::URA4+ leu1-32 ura4-D18 ade6-M216	this work
FY3851	h+ Δelp3::KanMX6 leu1-32 ura4-D18 ade6-M21?	this work
FY3854	h+ Δgcn5::KanMX4 mst2::URA4+ elp3::KanMX6 leu1-32 ura4-D18 ade6-M210	this work
Hu799	h- Δgcn5::KanMX4, leu1-32, ura4-D18, ade6-M210	Johnsson A 2006
Hu990	h- Δgcn5::KanMX4, mst2::ura4+, leu1-32, ura4-D18, ade6-M210	this work

This table lists the genotypes and origins for the strains used in this study.

### Deletion of *elp3*^+^

The *elp3 *deletion strain was created from a diploid strain purchased from Bioneer http://www.bioneer.com. The strain (*h+/h+, elp3+/elp3::KanMX6, leu1-32/leu1-32, ura4-D18/ura4-D18, ade6-M210/ade6-M216*) was haplodized by TBZ treatment to induce mitotic instability and chromosome loss. The cells were plated on YE media without adenine to select for red and pink colonies followed by selection for G418 resistant clones. The isolates were checked with PCR to assure the deletion and KanMX6 cassette was in the right place then back-crossed into a *wt *strain (*h*^-^, *leu1-32, ura4-D18, ade6-M216*).

### Growth Assays

Wild type and Δ*elp3 *cells were grown to stationary phase >1 × 10^8 ^cells/ml and then diluted in YES media to four different concentration 1 × 10^4^, 1 × 10^5^, 4 × 10^5 ^and 1 × 10^6 ^cells/ml respectively and grown at 30°C. Cells were counted regularly for 48 hours generating growth curves to cover the beginning and end of the exponential growth.

### Damage assays

Cells were grown to mid-log phase, serially diluted five-fold and spotted onto YES plates or YES plates were irradiated with the indicated doses of UV irradiation using a Stratalinker (Stratagene) or YES plates containing 10 or 12 μg/ml thiabendazole (TBZ), 5 mM hydroxyurea (HU), 7 μM camptothecin (CPT), 0.005% methyl methanesulfonate (MMS) and incubated for 2-3 days at 32°C. For the salt sensitivity assays cells were plated out in five-fold dilution series on rich media (YES) plates with or without KCl (1 M or 1.5 M) or CaCl2 (0.1 M or 0.25 M) at either 30°C or 36°C for 2 to 4 days.

### RNA prep and Microarray protocols for Affymetrix and Eurogenetech

RNA was isolated from three independent isolates growing at 30-32 degrees Celsius using phenol/chloroform extraction [45], phase lock gels (Eppendorf) and resuspended into TE after ethanol precipitation. Biotinylated cRNA was prepared for Affymetrix array analysis from 5 ug Total RNA using the standard Affymetrix one-cycle target labeling protocol. Samples were assayed for gene expression using Affymetrix GeneChip Yeast Genome 2.0 arrays consisting of probe sets representing 5,031 genes in *S. pombe *based on Sanger (June 2004). Data was analyzed using the R statistical environment. Affymetrix CEL files were processed and normalized using RMA [46]. The linear modeling package Limma [47] was used to calculate p-values and derive gene expression coefficients and to identify differentially expressed genes. The data discussed in this publication have been deposited in NCBI's Gene Expression Omnibus [48] and are accessible through GEO Series accession numbers GSE17259 and GSE17262 http://www.ncbi.nlm.nih.gov/geo/query/acc.cgi?token=dzmlrwoyekgmypk&acc=GSE17298. For microarrays using the Eurogenetech platform (salt sensitivity analysis), sample collection, processing and normalization was performed as in [28]. Genes were considered differentially expressed if the mutant/wt ratio was greater than 3.25 log 2 fold, with a p-value of 0.05 or less. Additional File 5 lists genes differentially expressed with a mutant/wt ratio of 1.7 log 2 fold, with a p-value of 0.05 or less.

### qPCR

Primers for the entire *S. pombe *genome were created by Chris Seidel using uPrimer. Total RNA in the amount of 2-4 ug was treated with DNase (Turbo DNase Treatment Ambion #AM1907) and 200-600 ng of the treated RNA was converted to cDNA by RT-PCR with the use of two primers. The concentration and sequence for the primers are 2 ng/uL final for the random primer pdN-15 and 20 ng/uL final for the anchored oligo-dT OdT-19N. A Corbett robot was used to load PCR reactions in 384-well plate format for analysis on an ABI-7900. Standard curves for the 9 primer pairs were made with 5 ten-fold dilutions of gDNA starting with 1000 ng/15 uL qPCR reaction and ending with 0.1 ng/15 uL qPCR reaction. The qPCR experiment consisted of measuring 9 loci in 24 samples three times each. 8 ng of the cDNA was used in each qPCR reaction without purification of the RT reaction. The final primer concentration was 500 nM each.

### Venn diagram program, GO term analysis

Venn diagrams were made using VENNY (Oliveros, J.C. (2007) VENNY. An interactive tool for comparing lists with Venn Diagrams http://bioinfogp.cnb.csic.es/tools/venny/index.html). Gene ontology classifications were classified at http://amigo.geneontology.org with the database filter set as GeneDB *S. pombe *with a threshold of maximum p value of 0.05 and minimum number of gene products of 2. We selected for publication only the biological process classifications.

### Histone acetylation

Cell lysates was obtained as described by [49] with the following modifications; the volume of the cell cultures were increased 2.5 times to get enough material for 10 or more gels. Briefly cells were inoculated in YES and grown overnight (o/n) to 1 × 10^7 ^cells/ml in a total volume of 25 ml and harvested in room temperature (RT) by centrifugation, washed once in H_2_O then resuspended in 0.75 ml H_2_O and an equal volume of 0.6 M NaOH, incubated 10 minutes at RT. Cells were removed from the NaOH by centrifugation and dissolved in 175 μl of modified SDS-sample buffer (60 mMTris-HCl, pH 6.8, 4% β-mercaptethanol, 4% SDS, 0.01% BPB, and 5% glycerol). Approximately 15 μl were used to run on precast gradient gels (Bio-Rad, 161-1105). Transfer was performed using the iBlot™ Dry Blotting system (Invitrogen). After transfer the membranes were blocked in in PBS containing 3% milk for 30 min at RT followed by o/n incubation at 4°C with primary antibody (anti-histones); or blocked o/n at 4°C followed by incubation with anti-actin antibody, at a 1:12 000 dilution (ms ab8224, Abcam) 1 hour at RT. For detection of histones antibodies the following dilutions were used: H3 Cter 1:200 (ab1791, Abcam); H3K9Ac 1:500 (07-352, Upstate); H3K14Ac 1:200 (07-353, Upstate); H3K18 Ac 1:200 (07-354, Upstate). For detection IgG horseradish peroxidase (GE Healthcare) against mouse and rabbit respectively was used along with ECL Plus (GE Healthcare) and exposed to film for 20 seconds to 3 minutes.

## Authors' contributions

RLN performed, designed and analyzed experiments and wrote the manuscript. AJ performed, designed and analyzed experiments and wrote the manuscript. BF performed and analyzed experiments. MG performed and analyzed experiments. YXF performed and analyzed experiments. CS designed and analyzed experiments and provided reagents. APW performed, designed and analyzed experiments, and provided reagents. SLF designed and analyzed experiments, wrote the manuscript and provided reagents.

## Supplementary Material

Additional file 1 **GO terms differentially regulated in HAT mutants**. This table lists the down and up-regulated gene ontology (GO) terms of mutant HATs. Click here for file

Additional file 2 **Differentially regulated genes (3.25 fold) in the triple HAT mutant**. This table lists the down and up-regulated genes of the triple HAT mutant compared to wild-type using an Affymetrix microarray. Click here for file

Additional file 3 **Differentially expressed genes (3.25 fold) in double HAT mutants**. This table lists the differentially changed genes in the double HAT mutants compared to wild-type using an Affymetrix microarray. Click here for file

Additional file 4 **Genes with 2 fold change in gene expression after exposure to salt**. This table lists the down and up-regulated genes after addition of salt using of mutant HATs using an Eurogentech microarray. Click here for file

Additional file 5 **Differentially expressed gene (1.7 fold) in HAT mutants**. This table lists the differentially changed genes in the HAT mutants compared to wild-type using an Affymetrix microarray. Click here for file

## References

[B1] Jenuwein T Allis CD Translating the histone code Science 2001 293 5532 1074 1080 10.1126/science.1063127 11498575

[B2] Kouzarides T Chromatin modifications and their function Cell 2007 128 4 693 705 10.1016/j.cell.2007.02.005 17320507

[B3] Lee KK Workman JL Histone acetyltransferase complexes: one size doesn't fit all Nat Rev Mol Cell Biol 2007 8 4 284 295 10.1038/nrm2145 17380162

[B4] Shogren-Knaak M Ishii H Sun JM Pazin MJ Davie JR Peterson CL Histone H4-K16 acetylation controls chromatin structure and protein interactions Science 2006 311 5762 844 847 10.1126/science.1124000 16469925

[B5] Berger SL Histone modifications in transcriptional regulation Curr Opin Genet Dev 2002 12 2 142 148 10.1016/S0959-437X(02)00279-4 11893486

[B6] Pillus L MYSTs mark chromatin for chromosomal functions Curr Opin Cell Biol 2008 20 3 326 333 10.1016/j.ceb.2008.04.009 18511253 PMC2717219

[B7] Allis CD Berger SL Cote J Dent S Jenuwien T Kouzarides T Pillus L Reinberg D Shi Y Shiekhattar R New nomenclature for chromatin-modifying enzymes Cell 2007 131 4 633 636 10.1016/j.cell.2007.10.039 18022353

[B8] Shevchenko A Roguev A Schaft D Buchanan L Habermann B Sakalar C Thomas H Krogan NJ Stewart AF Chromatin Central: towards the comparative proteome by accurate mapping of the yeast proteomic environment Genome Biol 2008 9 11 R167 10.1186/gb-2008-9-11-r167 19040720 PMC2614481

[B9] Sapountzi V Logan IR Robson CN Cellular functions of TIP60 Int J Biochem Cell Biol 2006 38 9 1496 1509 10.1016/j.biocel.2006.03.003 16698308

[B10] Clarke AS Lowell JE Jacobson SJ Pillus L Esa1p is an essential histone acetyltransferase required for cell cycle progression Mol Cell Biol 1999 19 4 2515 2526 10082517 10.1128/mcb.19.4.2515 PMC84044

[B11] Gomez EB Espinosa JM Forsburg SL Schizosaccharomyces pombe mst2+ encodes a MYST family histone acetyltransferase that negatively regulates telomere silencing Mol Cell Biol 2005 25 20 8887 8903 10.1128/MCB.25.20.8887-8903.2005 16199868 PMC1265769

[B12] Ehrenhofer-Murray AE Rivier DH Rine J The role of Sas2, an acetyltransferase homologue of Saccharomyces cerevisiae, in silencing and ORC function Genetics 1997 145 4 923 934 9093847 10.1093/genetics/145.4.923 PMC1207897

[B13] Reifsnyder C Lowell J Clarke A Pillus L Yeast SAS silencing genes and human genes associated with AML and HIV-1 Tat interactions are homologous with acetyltransferases Nat Genet 1996 14 1 42 49 10.1038/ng0996-42 8782818

[B14] Carrozza MJ Utley RT Workman JL Cote J The diverse functions of histone acetyltransferase complexes Trends Genet 2003 19 6 321 329 10.1016/S0168-9525(03)00115-X 12801725

[B15] Suka N Luo K Grunstein M Sir2p and Sas2p opposingly regulate acetylation of yeast histone H4 lysine16 and spreading of heterochromatin Nat Genet 2002 32 3 378 383 10.1038/ng1017 12379856

[B16] John S Howe L Tafrov ST Grant PA Sternglanz R Workman JL The something about silencing protein, Sas3, is the catalytic subunit of NuA3, a yTAF(II)30-containing HAT complex that interacts with the Spt16 subunit of the yeast CP (Cdc68/Pob3)-FACT complex Genes Dev 2000 14 10 1196 1208 10817755 PMC316621

[B17] Eberharter A John S Grant PA Utley RT Workman JL Identification and analysis of yeast nucleosomal histone acetyltransferase complexes Methods 1998 15 4 315 321 10.1006/meth.1998.0635 9740719

[B18] Sterner DE Berger SL Acetylation of histones and transcription-related factors Microbiol Mol Biol Rev 2000 64 2 435 459 10.1128/MMBR.64.2.435-459.2000 10839822 PMC98999

[B19] Baker SP Grant PA The SAGA continues: expanding the cellular role of a transcriptional co-activator complex Oncogene 2007 26 37 5329 5340 10.1038/sj.onc.1210603 17694076 PMC2746020

[B20] Vogelauer M Wu J Suka N Grunstein M Global histone acetylation and deacetylation in yeast Nature 2000 408 6811 495 498 10.1038/35044127 11100734

[B21] Winkler GS Kristjuhan A Erdjument-Bromage H Tempst P Svejstrup JQ Elongator is a histone H3 and H4 acetyltransferase important for normal histone acetylation levels in vivo Proc Natl Acad Sci USA 2002 99 6 3517 3522 10.1073/pnas.022042899 11904415 PMC122555

[B22] Li F Lu J Han Q Zhang G Huang B The Elp3 subunit of human Elongator complex is functionally similar to its counterpart in yeast Mol Genet Genomics 2005 273 3 264 272 10.1007/s00438-005-1120-2 15902492

[B23] Wittschieben BO Fellows J Du W Stillman DJ Svejstrup JQ Overlapping roles for the histone acetyltransferase activities of SAGA and elongator in vivo EMBO J 2000 19 12 3060 3068 10.1093/emboj/19.12.3060 10856249 PMC203375

[B24] Kristjuhan A Walker J Suka N Grunstein M Roberts D Cairns BR Svejstrup JQ Transcriptional inhibition of genes with severe histone H3 hypoacetylation in the coding region Mol Cell 2002 10 4 925 933 10.1016/S1097-2765(02)00647-0 12419235 PMC9035295

[B25] Kristjuhan A Wittschieben BO Walker J Roberts D Cairns BR Svejstrup JQ Spreading of Sir3 protein in cells with severe histone H3 hypoacetylation Proc Natl Acad Sci USA 2003 100 13 7551 7556 10.1073/pnas.1332299100 12796514 PMC164624

[B26] Howe L Auston D Grant P John S Cook RG Workman JL Pillus L Histone H3 specific acetyltransferases are essential for cell cycle progression Genes Dev 2001 15 23 3144 3154 10.1101/gad.931401 11731478 PMC312843

[B27] Helmlinger D Marguerat S Villen J Gygi SP Bahler J Winston F The S. pombe SAGA complex controls the switch from proliferation to sexual differentiation through the opposing roles of its subunits Gcn5 and Spt8 Genes Dev 2008 22 22 3184 3195 10.1101/gad.1719908 19056896 PMC2593614

[B28] Johnsson A Xue-Franzen Y Lundin M Wright AP Stress-specific role of fission yeast Gcn5 histone acetyltransferase in programming a subset of stress response genes Eukaryot Cell 2006 5 8 1337 1346 10.1128/EC.00101-06 16896217 PMC1539148

[B29] Robert F Pokholok DK Hannett NM Rinaldi NJ Chandy M Rolfe A Workman JL Gifford DK Young RA Global position and recruitment of HATs and HDACs in the yeast genome Mol Cell 2004 16 2 199 209 10.1016/j.molcel.2004.09.021 15494307 PMC3004369

[B30] Wittschieben BO Otero G de Bizemont T Fellows J Erdjument-Bromage H Ohba R Li Y Allis CD Tempst P Svejstrup JQ A novel histone acetyltransferase is an integral subunit of elongating RNA polymerase II holoenzyme Mol Cell 1999 4 1 123 128 10.1016/S1097-2765(00)80194-X 10445034

[B31] Tamburini BA Tyler JK Localized histone acetylation and deacetylation triggered by the homologous recombination pathway of double-strand DNA repair Mol Cell Biol 2005 25 12 4903 4913 10.1128/MCB.25.12.4903-4913.2005 15923609 PMC1140608

[B32] Hansen KR Ibarra PT Thon G Evolutionary-conserved telomere-linked helicase genes of fission yeast are repressed by silencing factors, RNAi components and the telomere-binding protein Taz1 Nucleic Acids Res 2006 34 1 78 88 10.1093/nar/gkj415 16407326 PMC1326240

[B33] Mandell JG Goodrich KJ Bahler J Cech TR Expression of a RecQ helicase homolog affects progression through crisis in fission yeast lacking telomerase J Biol Chem 2005 280 7 5249 5257 10.1074/jbc.M412756200 15591066

[B34] Aslett M Wood V Gene Ontology annotation status of the fission yeast genome: preliminary coverage approaches 100% Yeast 2006 23 13 913 919 10.1002/yea.1420 17072883

[B35] Schmittgen TD Livak KJ Analyzing real-time PCR data by the comparative C(T) method Nat Protoc 2008 3 6 1101 1108 10.1038/nprot.2008.73 18546601

[B36] Yamada T Mizuno K Hirota K Kon N Wahls WP Hartsuiker E Murofushi H Shibata T Ohta K Roles of histone acetylation and chromatin remodeling factor in a meiotic recombination hotspot EMBO J 2004 23 8 1792 1803 10.1038/sj.emboj.7600138 14988732 PMC394230

[B37] Johnsson A Durand-Dubief M Xue-Franzén y Rönnerblad M Ekwall K Wright A HAT-HDAC interplay modulates global histone H3K14 acetylation in gene coding regions during stress Embo Reports 2009 in press 19633696 10.1038/embor.2009.127 PMC2750064

[B38] Gomez EB Nugent RL Laria S Forsburg SL Schizosaccharomyces pombe histone acetyltransferase Mst1 (KAT5) is an essential protein required for damage response and chromosome segregation Genetics 2008 179 2 757 771 10.1534/genetics.107.085779 18505873 PMC2429872

[B39] Huang B Johansson MJ Bystrom AS An early step in wobble uridine tRNA modification requires the Elongator complex RNA 2005 11 4 424 436 10.1261/rna.7247705 15769872 PMC1370732

[B40] Svejstrup JQ Elongator complex: how many roles does it play? Curr Opin Cell Biol 2007 19 3 331 336 10.1016/j.ceb.2007.04.005 17466506

[B41] Han Q Lu J Duan J Su D Hou X Li F Wang X Huang B Gcn5- and Elp3-induced histone H3 acetylation regulates hsp70 gene transcription in yeast Biochem J 2008 409 3 779 788 10.1042/BJ20070578 17910533

[B42] Pokholok DK Harbison CT Levine S Cole M Hannett NM Lee TI Bell GW Walker K Rolfe PA Herbolsheimer E Genome-wide map of nucleosome acetylation and methylation in yeast Cell 2005 122 4 517 527 10.1016/j.cell.2005.06.026 16122420

[B43] Glozak MA Sengupta N Zhang X Seto E Acetylation and deacetylation of non-histone proteins Gene 2005 363 15 23 10.1016/j.gene.2005.09.010 16289629

[B44] van Attikum H Gasser SM The histone code at DNA breaks: a guide to repair? Nat Rev Mol Cell Biol 2005 6 10 757 765 10.1038/nrm1737 16167054

[B45] Forsburg SL Rhind N Basic methods for fission yeast Yeast 2006 23 3 173 183 10.1002/yea.1347 16498704 PMC5074380

[B46] Irizarry RA Hobbs B Collin F Beazer-Barclay YD Antonellis KJ Scherf U Speed TP Exploration, normalization, and summaries of high density oligonucleotide array probe level data Biostatistics 2003 4 2 249 264 10.1093/biostatistics/4.2.249 12925520

[B47] Smyth GK Linear models and empirical bayes methods for assessing differential expression in microarray experiments Stat Appl Genet Mol Biol 2004 3 Article3 16646809 10.2202/1544-6115.1027

[B48] Edgar R Domrachev M Lash AE Gene Expression Omnibus: NCBI gene expression and hybridization array data repository Nucleic Acids Res 2002 30 1 207 210 10.1093/nar/30.1.207 11752295 PMC99122

[B49] Matsuo Y Asakawa K Toda T Katayama S A rapid method for protein extraction from fission yeast Biosci Biotechnol Biochem 2006 70 8 1992 1994 10.1271/bbb.60087 16926515

