# Supplementary material for: Expression profiling of S. pombe acetyltransferase mutants identifies redundant pathways of gene regulation
Source: BMC Genomics. 2010 Jan 22;11:59. doi: 10.1186/1471-2164-11-59 (PMC2823694; doi:10.1186/1471-2164-11-59)
Supplement: Additional file 1 — GO terms differentially regulated in HAT mutants. This table lists the down and up-regulated gene ontology (GO) terms of mutant HATs. [file 1471-2164-11-59-S1.PDF]

Additional File 1: GO terms differentially regulated in HAT mutants

Down-regulated GO Terms

GO annotation	Number of genes	Frequency (%)
Δelp3		
substrate-specific transmembrane transporter activity	2	28.6
substrate-specific transporter activity	2	28.6
transmembrane transporter activity	2	28.6
Δgcn5 Δelp3		
iron assimilation	2	9.1
cellular di-, tri-valent inorganic cation homeostasis	3	13.6
di-, tri-valent inorganic cation homeostasis	3	13.6
iron ion transport	2	9.1
iron ion homeostasis	2	9.1
cellular iron ion homeostasis	2	9.1
di-, tri-valent inorganic cation transport	2	9.1
cellular cation homeostasis	3	13.6
cellular ion homeostasis	3	13.6
cellular chemical homeostasis	3	13.6
cation homeostasis	3	13.6
transition metal ion transport	2	9.1
ion homeostasis	3	13.6
cellular homeostasis	3	13.6
chemical homeostasis	3	13.6
ion transport	3	13.6
metal ion transport	2	9.1
cellular response to stress	6	27.3
cellular response to stimulus	6	27.3
response to oxidative stress	2	9.1
response to chemical stimulus	3	13.6
homeostatic process	3	13.6
response to stress	6	27.3
Δgcn5 Δmst2		
histone acetylation	2	5.7
amine transport	2	5.7
amino acid catabolic process	2	5.7
amino acid metabolic process	4	11.4
nitrogen compound catabolic process	2	5.7
amine catabolic process	2	5.7
positive regulation of transcription, DNA-dependent	2	5.7
positive regulation of RNA metabolic process	2	5.7
positive regulation of transcription	2	5.7
positive regulation of nucleobase, nucleoside, nucleotide and nucleic acid metabolic process	2	5.7
cellular amino acid and derivative metabolic process	4	11.4

positive regulation of biosynthetic process	2	5.7
positive regulation of macromolecule biosynthetic process	2	5.7
positive regulation of gene expression	2	5.7
protein amino acid acetylation	2	5.7
cellular amine metabolic process	4	11.4
positive regulation of cellular metabolic process	2	5.7
positive regulation of metabolic process	2	5.7
cellular nitrogen compound metabolic process	4	11.4
positive regulation of macromolecule metabolic process	2	5.7
nitrogen compound metabolic process	4	11.4
protein amino acid acylation	2	5.7
Δmst2 Δelp3		
galactose metabolic process	2	8.7
monosaccharide transport	2	8.7
disaccharide metabolic process	2	8.7
carbohydrate transport	2	8.7
cellular carbohydrate metabolic process	4	17.4
carbohydrate metabolic process	4	17.4
hexose metabolic process	2	8.7
monosaccharide metabolic process	2	8.7
cellular response to stress	6	26.1
cellular response to stimulus	6	26.1
Δgcn5 Δelp3 Δmst2		
histone acetylation	2	6.5
positive regulation of transcription, DNA-dependent	2	6.5
positive regulation of RNA metabolic process	2	6.5
positive regulation of transcription	2	6.5
positive regulation of nucleobase, nucleoside, nucleotide and nucleic acid metabolic process	2	6.5
positive regulation of biosynthetic process	2	6.5
positive regulation of macromolecule biosynthetic process	2	6.5
positive regulation of gene expression	2	6.5
protein amino acid acetylation	2	6.5
positive regulation of cellular metabolic process	2	6.5
positive regulation of metabolic process	2	6.5
positive regulation of macromolecule metabolic process	2	6.5
di-, tri-valent inorganic cation homeostasis	2	6.5
protein amino acid acylation	2	6.5

Up-regulated GO Terms

GO annotation[1]	Number of genes	Frequency[2] (%)
Δelp3		
GO:0006812 cation transport	2	15.4
GO:0006811 ion transport	2	15.4
Δgcn5		

GO:0032005 signal transduction during conjugation with cellular fusion	4	30.8
GO:0031137 regulation of conjugation with cellular fusion	4	30.8
GO:0046999 regulation of conjugation	4	30.8
GO:0043900 regulation of multi-organism process	4	30.8
GO:0019953 sexual reproduction	4	30.8
GO:0000746 conjugation	4	30.8
GO:0000747 conjugation with cellular fusion	4	30.8
GO:0051704 multi-organism process	4	30.8
GO:0000003 reproduction	5	38.5
GO:0000749 response to pheromone during conjugation with cellular fusion	2	15.4
GO:0019236 response to pheromone	2	15.4
GO:0007165 signal transduction	4	30.8
GO:0007154 cell communication	4	30.8
GO:0065007 biological regulation	6	46.2
GO:0050794 regulation of cellular process	5	38.5
GO:0050789 regulation of biological process	5	38.5
Δgcn5 Δelp3		
M phase of meiotic cell cycle	4	15.4
meiosis	4	15.4
meiotic cell cycle	4	15.4
amine transport	2	7.7
M phase	5	19.2
cell cycle phase	5	19.2
meiotic chromosome segregation	2	7.7
Δgcn5 Δmst2		
conjugation	13	24.1
conjugation with cellular fusion	13	24.1
sexual reproduction	13	24.1
multi-organism process	13	24.1
reproduction	15	27.8
signal transduction during conjugation with cellular fusion	5	9.3
regulation of conjugation with cellular fusion	7	13
regulation of conjugation	7	13
regulation of multi-organism process	7	13
cellular response to stress	17	31.5
cellular response to stimulus	17	31.5
monosaccharide transport	3	5.6
response to stimulus	19	35.2
response to stress	17	31.5
carbohydrate transport	3	5.6
positive regulation of meiosis	2	3.7
regulation of meiosis	3	5.6
regulation of meiotic cell cycle	3	5.6
response to pheromone during conjugation with cellular fusion	3	5.6
cell communication	9	16.7
response to pheromone	3	5.6
signal transduction	8	14.8

G-protein coupled receptor protein signaling pathway	2	3.7
MAPKKK cascade	2	3.7
protein kinase cascade	2	3.7
positive regulation of biological process	4	7.4
cell surface receptor linked signal transduction	2	3.7
regulation of cell cycle process	3	5.6
regulation of biological process	13	24.1
protein amino acid phosphorylation	4	7.4
positive regulation of cellular process	3	5.6
cellular response to nitrogen starvation	2	3.7
cellular response to nitrogen levels	2	3.7
alcohol catabolic process	2	3.7
cellular carbohydrate metabolic process	4	7.4
regulation of cellular process	12	22.2
Δmst2 Δelp3		
DNA recombination	2	11.8
cation transport	2	11.8
ion transport	2	11.8
Δgcn5 Δelp3 Δmst2		
iron assimilation	5	7.8
cellular di-, tri-valent inorganic cation homeostasis	7	10.9
di-, tri-valent inorganic cation homeostasis	7	10.9
iron assimilation by reduction and transport	3	4.7
signal transduction during conjugation with cellular fusion	5	7.8
iron ion homeostasis	5	7.8
cellular iron ion homeostasis	5	7.8
di-, tri-valent inorganic cation transport	5	7.8
iron ion transport	4	6.2
metal ion transport	6	9.4
transition metal ion transport	5	7.8
reproduction	11	17.2
cellular cation homeostasis	7	10.9
cellular ion homeostasis	7	10.9
cellular chemical homeostasis	7	10.9
cation homeostasis	7	10.9
ion homeostasis	7	10.9
cellular homeostasis	7	10.9
copper ion import	2	3.1
chemical homeostasis	7	10.9
conjugation	7	10.9
conjugation with cellular fusion	7	10.9
sexual reproduction	7	10.9
multi-organism process	7	10.9
regulation of conjugation with cellular fusion	5	7.8
regulation of conjugation	5	7.8
regulation of multi-organism process	5	7.8
homeostatic process	8	12.5

cation transport	6	9.4
siderophore transport	2	3.1
siderophore-iron transport	2	3.1
pheromone-dependent signal transduction during conjugation with cellular fusion	2	3.1
transmembrane transport	4	6.2
response to pheromone during conjugation with cellular fusion	3	4.7
ion transport	6	9.4
transmembrane ion transport	3	4.7
iron assimilation by chelation and transport	2	3.1
regulation of biological quality	9	14.1
copper ion transport	2	3.1
response to pheromone	3	4.7
G-protein coupled receptor protein signaling pathway	2	3.1
biological regulation	19	29.7
response to stimulus	16	25
cell development	3	4.7
meiotic gene conversion	2	3.1
cell surface receptor linked signal transduction	2	3.1
cellular response to stress	12	18.8
cellular response to stimulus	12	18.8
sex determination	2	3.1
mating type determination	2	3.1
reproductive developmental process	2	3.1
signal transduction	7	10.9
M phase of meiotic cell cycle	5	7.8
meiosis	5	7.8
meiotic cell cycle	5	7.8
mst1ts		
cellular response to stress	50	42.2
cellular response to stimulus	50	42.4
response to stress	50	42.4
response to stimulus	52	44.1
cellular carbohydrate catabolic process	7	5.9
copper ion import	2	1.7
alcohol catabolic process	5	4.2
cellular carbohydrate metabolic process	10	8.5
iron assimilation	3	2.5
glucose catabolic process	4	3.4
hexose catabolic process	4	3.4
carbohydrate metabolic process	10	8.5
NADPH regeneration	3	2.5
pentose-phosphate shunt	3	2.5
NADP metabolic process	3	2.5
glucose 6-phosphate utilization	2	1.7
siderophore transport	2	1.7
siderophore-iron transport	2	1.7
cellular di-, tri-valent inorganic cation homeostasis	5	4.2
monosaccharide catabolic process	4	3.4

di-, tri-valent inorganic cation homeostasis	5	4.2
glucose metabolic process	4	3.4
glucose 6-phosphate metabolic process	2	1.7
pentose-phosphate shunt, oxidative branch	2	1.7
iron assimilation by chelation and transport	2	1.7
copper ion transport	2	1.7
nicotinamide metabolic process	3	2.5
cellular iron ion homeostasis	3	2.5
iron ion homeostasis	3	2.5
di-, tri-valent inorganic cation transport	3	2.5
polyamine transport	2	1.7
hexose metabolic process	4	3.4
cation homeostasis	6	5.1
ion homeostasis	6	5.1
pyridine nucleotide metabolic process	3	2.5
metal ion transport	4	3.4
chemical homeostasis	6	5.1
disaccharide metabolic process	2	1.7
monosaccharide metabolic process	4	3.4
iron ion transport	2	1.7
transition metal ion transport	3	2.5
amine transport	3	2.5
cellular alcohol metabolic process	6	5.1
cellular cation homeostasis	5	4.2
cellular ion homeostasis	5	4.2
cellular chemical homeostasis	5	4.2
cation transport	5	4.2

This table lists the down and up-regulated gene ontology (GO) terms of mutant HATs.
