# Supplementary material for: Expression profiling of S. pombe acetyltransferase mutants identifies redundant pathways of gene regulation
Source: BMC Genomics. 2010 Jan 22;11:59. doi: 10.1186/1471-2164-11-59 (PMC2823694; doi:10.1186/1471-2164-11-59)
Supplement: Additional file 2 — Differentially regulated genes (3.25 fold) in the triple HAT mutant. This table lists the down and up-regulated genes of the triple HAT mutant compared to wild-type using an Affymetrix microarray. [file 1471-2164-11-59-S2.PDF]

Additional File 2: Differentially regulated genes (3.25 fold) in the triple HAT mutant

Down-regulated genes

	Δgcn5Δmst2Δelp3			Δgcn5Δmst2Δelp3	
Gene	Log2 Change	p-value	Gene	Log2 Change	p-value
gcn5	-5.66	0.004193963	SPAC869.05c	-2.21	0.01125063
elp3	-5.64	3.29E-13	SPAC1F12.03c	-2.17	0.000970933
SPBPB10D8.01	-4.74	5.16E-06	SPAC18G6.12c	-2.15	0.000137484
SPBPB10D8.02c	-4.74	7.09E-06	SPBC947.04	-2.11	0.001731022
mst2	-4.41	0.01017041	SPCC622.01c	-2.09	1.73E-06
SPAC57A10.06	-3.73	0.002283381	SPCC1902.02	-2.08	0.000234049
SPBPB2B2.06c	-3.65	5.26E-05	SPAC589.09	-2.08	6.64E-05
SPBC26H8.11c	-3.64	1.20E-08	SPBC25B2.08	-2.05	3.28E-05
SPAC1039.02	-3.40	0.001562479	SPCC1682.09c	-2.04	3.76E-05
SPAC2E1P3.05c	-3.17	1.71E-10	SPAC1399.04c	-2.03	0.005854158
SPBPB2B2.01	-2.88	0.000283339	Msa1	-2.02	3.86E-05
SPAC186.06	-2.78	0.001161544	SPCC1223.13	-1.93	0.005773073
SPAC977.05c	-2.60	0.00556226	SPBP26C9.03c	-1.89	0.031834321
SPAC186.05c	-2.53	0.001728652	isp4	-1.83	2.38E-05
SPAC29B12.10c	-2.53	0.000532633	erg28	-1.81	1.10E-07
SPBC16A3.16	-2.39	0.002041561	SPBC21D10.07	-1.80	3.03E-07
SPAC186.03	-2.36	3.93E-05	hhf1	-1.76	1.68E-05

up-regulated genes

Gene	Δgcn5Δmst2Δelp3 Log2 Change	p-value	Gene	Δgcn5Δmst2Δelp3 Log2 Change	p-value
crp79	1.83	3.64E-06	Mam2	2.39	0.031460047
rec24	1.83	4.67E-06	SPBC1685.13	2.39	0.009443801
SPAC6C3.07	1.79	2.44E-06	ssa1	2.41	0.009228639
meu17	1.78	2.72E-07	SPBC2G2.17c	2.42	5.92E-05
tht1	1.76	9.05E-06	SPAC922.03	2.52	1.72E-10
SPCC4G3.03	1.75	4.36E-05	SPAC1952.04c	2.53	0.00012367
caf5	1.89	0.008182728	Zym1	2.53	0.002346264
SPAC4F10.08	1.91	4.96E-07	meu14	2.67	9.57E-08
SPAC1F12.10c	1.92	0.029187088	ste6	2.67	0.002090987
SPBC1289.16c	1.92	0.009158305	SPAC1F8.08	2.67	6.91E-06
SPBC725.10	1.95	0.015549669	SPBC19C7.04c	2.68	0.002745215
SPBC1685.14c	1.96	6.09E-05	wtf20	2.72	3.34E-05
SPBC354.08c	1.97	8.65E-05	spk1	2.76	0.010948439
SPAC27D7.09c	1.98	0.007841458	mei2	2.82	0.025390591
SPCC1020.09	1.99	0.002301415	SPBC359.06	2.98	0.034921984
SPAC29A4.12c	2.02	0.000921543	fio1	3.06	0.001454197
SPCPB16A4.06c	2.02	0.000150191	SPBC947.05c	3.29	1.29E-08
SPCC74.02c	2.02	5.55E-07	mfm1	3.36	0.026192367
ste11	2.03	0.012048872	dak2	3.41	4.14E-07
rho5	2.03	0.000814839	SPBC56F2.06	3.51	0.000275571
cut2	2.04	7.84E-05	SPCC737.04	3.55	7.02E-05
spo6	2.04	2.92E-06	spn6	3.56	0.000934784
SPCC70.04c	2.06	7.02E-05	SPCC1739.08c	3.56	0.046079337
SPBC1685.05	2.07	5.19E-07	cta3	3.64	1.71E-11
SPAC14C4.01c	2.07	6.99E-05	SPBC23G7.10c	4.12	0.002206808
fip1	2.17	0.007511319	ght3	4.18	0.03579791
mcp3	2.18	3.18E-06	frp1	4.31	0.000260879
SPCC338.18	2.19	0.001391778	map2	4.47	1.54E-11
mde2	2.20	0.000144052	SPAC3G9.11c	4.62	6.82E-08
ppk33	2.22	0.003466142	matmc_1	5.33	0.016405454
SPCC1840.12	2.25	8.28E-05	mfm2	6.13	0.015267126
SPCC330.04c	2.25	2.32E-08	SPAC1F8.02c	6.36	4.47E-12
SPCC777.04	2.26	0.003985904	SPBCPT2R1.08c	6.37	0.000188046
SPAC4H3.03c	2.32	0.004334504	str3	7.18	1.76E-07
dmc1	2.33	4.79E-05			

map1	2.34	0.00281451
SPAC6B12.03c	2.37	0.003999216

This table lists the down and up-regulated genes of the triple HAT mutant compared to wild-type using an Affymetrix microarray.
