# Supplementary material for: Expression profiling of S. pombe acetyltransferase mutants identifies redundant pathways of gene regulation
Source: BMC Genomics. 2010 Jan 22;11:59. doi: 10.1186/1471-2164-11-59 (PMC2823694; doi:10.1186/1471-2164-11-59)
Supplement: Additional file 3 — Differentially expressed genes (3.25 fold) in double HAT mutants. This table lists the differentially changed genes in the double HAT mutants compared to wild-type using an Affymetrix microarray. [file 1471-2164-11-59-S3.PDF]

Additional File 3: Differentially expressed genes (3.25 fold) in double HAT mutants

Down-regulated

	Δgcn5Δelp3			Δgcn5Δmst2			Δmst2Δelp3	
Gene	Log2 Change	p-value	Gene	Log2 Change	p-value	Gene	Log2 Change	p-value
elp3	-5.81	3.53E-15	his3	-7.51	3.17E-04	elp3	-5.45	1.53E-15
gcn5	-5.62	6.60E-04	gcn5	-5.32	3.85E-04	SPBPB2B2.09c	-5.12	1.07E-13
SPAC57A10.06	-3.46	5.93E-04	SPBPB10D8.02c	-5.00	2.88E-08	SPBPB2B2.06c	-4.69	1.86E-08
SPCC794.04c	-3.34	1.09E-04	SPBPB10D8.01	-4.81	3.53E-08	mst2	-4.37	8.06E-04
SPBPB2B2.08	-2.75	1.11E-02	SPBPB2B2.06c	-4.77	1.43E-08	SPBPB2B2.05	-3.73	4.40E-04
SPBC215.11c	-2.67	5.73E-05	SPAC1039.02	-4.73	8.49E-07	SPBPB10D8.02c	-3.70	1.98E-06
isp5	-2.41	1.82E-02	SPAC186.05c	-4.59	2.32E-08	SPBPB2B2.10c	-3.62	2.63E-06
SPAC869.05c	-2.32	1.45E-03	mst2	-4.41	7.41E-04	SPBPB2B2.08	-3.40	1.02E-03
gst2	-2.09	3.99E-02	SPBPB2B2.05	-4.06	1.93E-04	SPBPB2B2.13	-3.36	2.77E-04
SPAC18G6.12c	-2.08	1.37E-05	SPAC977.14c	-4.05	4.22E-04	SPCC794.04c	-3.24	4.43E-05
SPCC569.03	-2.06	8.21E-06	SPAC186.03	-3.63	7.22E-10	SPBPB2B2.01	-3.02	3.01E-06
SPAC23H3.15c	-2.05	4.57E-03	SPAC186.06	-3.41	2.97E-06	SPBC660.05	-2.94	1.64E-03
srx1	-2.04	4.26E-04	SPBPB2B2.01	-3.30	9.24E-07	SPAC22F8.05	-2.58	8.09E-05
SPCC663.09c	-2.00	1.04E-04	SPAC57A10.06	-3.11	5.55E-04	SPAC57A10.06	-2.58	2.76E-03
zym1	-1.99	2.51E-03	SPBC359.04c	-2.98	1.81E-09	str1	-2.44	5.05E-05
fip1	-1.98	2.69E-03	SPBPB21E7.10	-2.89	1.02E-05	SPAC869.05c	-2.41	3.81E-04
SPAC29B12.10c	-1.95	6.76E-04	sou1	-2.87	5.39E-09	ght5	-2.40	2.87E-08
SPBC27.05	-1.93	1.65E-05	SPBC947.04	-2.80	1.92E-06	SPAC23H3.15c	-2.32	6.68E-04
SPAC1F12.03c	-1.92	3.52E-04	SPBPB2B2.08	-2.59	8.09E-03	SPCC569.03	-2.22	6.85E-07
SPBC16A3.16	-1.90	1.98E-03	SPAC977.05c	-2.38	7.28E-04	inv1	-2.13	1.48E-02
str1	-1.89	1.79E-03	SPBC26H8.11c	-2.35	4.53E-08	SPBPB10D8.01	-2.10	8.44E-04
SPBPB2B2.01	-1.88	1.61E-03	obr1	-2.35	9.26E-04	SPBC16A3.16	-1.97	5.65E-04
SPCC1902.02	-1.80	8.76E-05	SPCC663.09c	-2.34	4.39E-06	ght4	-1.89	8.80E-03
SPAC17A5.09c	-1.79	2.52E-08	SPBC1683.02	-2.31	2.23E-06	SPCC622.05	-1.85	1.07E-04
isp4	-1.76	2.08E-06	SPAC977.15	-2.26	3.04E-02	isp4	-1.74	5.36E-07
			fta5	-2.17	1.44E-06			
			SPBC3H7.07c	-2.12	3.86E-07			

SPAC23C4.06c	-2.10	3.77E-05
SPCC1223.13	-2.09	1.45E-04
SPCC569.05c	-2.07	7.09E-04
SPAC24B11.14	-2.07	2.73E-03
SPAC869.05c	-2.05	1.65E-03
SPBPB2B2.18	-1.95	1.44E-03
SPAC8C9.05	-1.94	5.72E-07
SPBC359.03c	-1.91	1.26E-11

Up-regulated genes

	Δgcn5Δelp3			Δgcn5Δmst2			Δmst2Δelp3	
Gene	Log2 Change	p-value	Gene	Log2 Change	p-value	Gene	Log2 Change	p-value
pma2	1.86	1.69 E-04	SPAC167.06c	1.80	1.42E-04	ctr5	1.87	4.14E-08
SPCC74.02c	1.86	6.50 E-08	ste4	1.97	9.48E-06	SPCC132.04c	1.86	9.18E-06
SPBC1861.06c	1.85	6.74 E-06	SPBC16E9.16c	2.04	3.18E-08	SPCC830.04c	1.85	1.78E-06
SPAC4F10.08	1.76	5.76 E-08	SPAC5H10.01	2.06	2.73E-02	mug97	1.84	9.60E-09
mpf1	1.89	4.35 E-06	SPCP31B10.06	2.06	1.01E-06	SPCC330.03c	1.81	1.20E-06
SPCC830.04c	1.91	4.97 E-06	SPAC31G5.07	2.07	6.02E-09	SPAC6C3.03c	1.92	1.73E-06
mei4	2.00	7.97 E-06	meu10	2.09	3.59E-07	SPAPB1A11.01	1.97	2.41E-07
SPBC1348.01	2.02	5.55 E-03	SPCC1393.12	2.09	1.29E-09	SPAC56F8.12	1.98	3.35E-08
SPBC354.08c	2.03	3.56 E-06	SPBC725.10	2.12	6.57E-04	mde2	2.00	7.89E-06
mam2	2.15	1.63 E-02	isp3	2.18	1.15E-05	SPBC2G2.17c	2.02	8.49E-06
SPAC977.07c	2.16	1.03 E-07	SPBC1289.16c	2.18	1.83E-04	spo6	2.03	2.28E-08

SPAC186.01	2.18	1.12E-06	SPCC777.04	2.19	2.67E-04	SPAPB24D3.07c	2.07	4.94E-03
mug97	2.20	3.50E-09	SPAC14C4.01c	2.19	4.47E-07	SPCC737.04	2.15	3.53E-04
ctr4	2.20	6.91E-07	tht1	2.23	2.19E-09	alr2	2.16	1.83E-09
SPAC750.07c	2.27	6.97E-05	SPAC13F5.03c	2.24	2.84E-05	SPBC1861.06c	2.19	1.62E-07
mde2	2.27	6.61E-06	SPCC70.04c	2.32	1.90E-07	SPAC2E1P3.02c	2.32	1.45E-08
SPAC750.04c	2.35	3.46E-07	SPACUNK4.17	2.32	3.65E-05	SPBC354.08c	2.71	1.31E-08
SPBPB8B6.03	2.35	1.43E-08	SPBC146.02	2.33	5.56E-08	SPAC977.15	2.79	9.58E-03
spo6	2.46	6.92E-09	SPCC794.04c	2.42	8.62E-04	ctr4	2.88	2.52E-09
SPCC777.04	2.48	2.27E-04	dak2	2.56	1.74E-07	SPBCPT2R1.08c	2.92	7.13E-03
spn5	2.58	4.26E-05	SPAC4H3.03c	2.57	7.41E-05	dak2	4.40	3.91E-11
mcp3	2.62	8.20E-09	SPBPB2B2.12c	2.63	2.11E-02	ura4	4.44	1.44E-02
SPAC1952.04c	2.66	4.36E-06	ght4	2.63	6.85E-04			
spn6	2.74	1.17E-03	SPCC338.18	2.63	4.83E-06			
SPAC2E1P3.02c	2.76	5.64E-09	SPBC21D10.06c	2.64	1.15E-12			
SPBPB8B6.02c	2.78	2.18E-13	SPAC1F8.08	2.65	6.91E-08			
grt1	2.81	1.09E-06	map3	2.65	5.28E-12			
alr2	3.05	4.16E-11	rho5	2.70	5.84E-07			
dak2	3.64	4.94E-09	wtf20	2.77	3.12E-07			
SPAC977.05c	4.56	1.72E-06	ran1	2.79	2.48E-06			
SPBCPT2R1.08c	5.16	8.64E-05	SPAC6B12.03c	2.86	2.44E-05			
			ste7	2.99	3.26E-05			

SPCC737.04	2.99	8.94E-06
map1	3.11	4.16E-06
ste11	3.19	7.81E-06
ght1	3.38	1.44E-02
ura4	3.53	4.12E-02
ste6	3.56	2.49E-06
agl1	3.59	1.78E-03
fbp1	3.67	5.79E-13
SPBC56F2.06	3.68	2.97E-06
SPAC3G9.11c	3.74	8.98E-09
shk2	3.80	2.81E-11
spn6	3.84	1.08E-05
ppk33	3.93	1.21E-07
mei2	4.01	1.14E-04
SPCC794.01c	4.08	6.89E-04
rgs1	4.08	5.34E-07
SPBC4.01	4.20	1.88E-08
SPCC1739.08c	4.27	1.92E-03
SPBC359.06	4.72	7.39E-05
spk1	4.76	1.85E-06
ght3	6.19	1.63E-04
map2	6.36	9.50E-17
SPBCPT2R1.08c	6.70	1.71E-06

Additional File 3 lists the differentially changed genes in the double HAT mutants compared to wild-type cells using an Affymetrix microarray.
