# Supplementary material for: Expression profiling of S. pombe acetyltransferase mutants identifies redundant pathways of gene regulation
Source: BMC Genomics. 2010 Jan 22;11:59. doi: 10.1186/1471-2164-11-59 (PMC2823694; doi:10.1186/1471-2164-11-59)
Supplement: Additional file 4 — Genes with 2 fold change in gene expression after exposure to salt. This table lists the down and up-regulated genes after addition of salt using of mutant HATs using an Eurogentech microarray. [file 1471-2164-11-59-S4.PDF]

Additional File 4: Genes with 2 fold change in gene expression after exposure to salt.

Down-regulated

2 fold down gcn5 KCl	annotation	2 fold down mst2 KCl	annotation
SPAC977.05c	conserved fungal family	SPBPB2B2.19c	S. pombe specific 5Tm protein family
SPBPB2B2.15	conserved fungal family	SPAC750.05c	S. pombe specific 5Tm protein family
SPBC1348.06c	conserved fungal family	SPAC977.01	S. pombe specific 5Tm protein family
SPAC5H10.06c	alcohol dehydrogenase Adh4	SPBC1348.02	S. pombe specific 5Tm protein family
SPAC22A12.17c	short chain dehydrogenase (predicted)	SPBP4G3.02	acid phosphatase Pho1
SPAC3C7.14c	ubiquitinated histone-like protein Uhp1	SPCC1020.10	serine/threonine protein kinase Oca2 (predicted)
SPBC8D2.16c	DUF171 family protein	SPAC212.04c	S. pombe specific DUF999 family protein 1
SPBC30D10.14	dienelactone hydrolase family	SPAC2H10.01	transcription factor, zf-fungal binuclear cluster type (predicted)
SPCC965.07c	glutathione S-transferase Gst2	SPAC57A7.11	WD repeat protein Mip1
SPBC2A9.02	NAD dependent epimerase/dehydratase family protein	SPAC1786.02	phospholipase (predicted)
SPCPJ732.01	retromer complex subunit Vps5	SPCC584.16c	sequence orphan
SPBC24C6.09c	phosphoketolase family protein (predicted)	SPBC11C11.01	RNA-binding protein
SPBC1271.08c	sequence orphan	SPAC1B3.16c	vitamin H transporter Vth1
SPAC977.14c	aldo/keto reductase, unknown biological role	SPCC1840.07c	phosphoprotein phosphatase (predicted)
SPCC16A11.15c	sequence orphan	SPBC651.12c	sequence orphan
SPAC9E9.11	pyridoxal reductase Plr1	SPBC660.08	sequence orphan
SPAC5H10.03	phosphoglycerate mutase family	SPAC750.01	pseudogene
SPAC22H10.13	metallothionein Zym1	SPCC1235.01	sequence orphan
SPAC17G8.04c	ARP2/3 actin-organizing complex subunit Arc5	SPBPB2B2.04	pseudo-very degraded transporter
SPAC1039.02	phosphoprotein phosphatase (predicted)	SPCC1919.13c	conserved eukaryotic protein
SPAC4G9.12	gluconokinase	SPCC132.04c	NAD-dependent glutamate dehydrogenase (predicted)
SPCC18B5.02c	cinnamoyl-CoA reductase pseudogene	SPAC977.06	S. pombe specific DUF999 family protein 3
SPAC3C7.11c	calnexin Cnx1	SPCC965.12	dipeptidyl peptidase (predicted)
SPAC750.01	pseudogene	SPAC2G11.02	ribosome biogenesis protein Urb2 (predicted)
SPCC1494.03	armadillo repeat containing, Zfs1 target number 1	SPBC19C7.04c	conserved fungal protein
SPAC27E2.04c	dubious	SPCP1E11.03	arrestin
SPCC569.05c	spermidine family transporter (predicted)	SPBC409.08	spermine family transporter (predicted)
SPAC637.03	conserved fungal protein	SPAC27D7.04	4-alpha-hydroxytetrahydrobiopterin dehydratase (predicted)
SPBC16D10.08c	heat shock protein Hsp104 (predicted)	SPBC1289.14	adducin
SPAC13C5.04	amidotransferase (predicted)	SPAPB8E5.07c	ribosome biogenesis protein Rrp12
SPBC21B10.08c	sequence orphan	SPAPB1A10.14	F-box protein
SPAC13C5.06c	sequence orphan	SPAC17A2.08c	sequence orphan
SPBC23G7.10c	NADH-dependent flavin oxidoreductase (predicted)	SPAC977.14c	aldo/keto reductase, unknown biological role
SPAC16A10.01	DUF1212 family protein	SPAC750.02c	membrane transporter
SPBC215.11c	aldo/keto reductase, unknown biological role	SPBCPT2R1.01c	S. pombe specific DUF999 protein family 9
SPAC7D4.09c	steroid dehydrogenase (predicted)	SPAC1B3.06c	UbiE family methyltransferase (predicted)
SPAC513.02	phosphoglycerate mutase family	SPCC24B10.19c	sequence orphan
SPBC4C3.03	homoserine kinase (predicted)	SPBC21C3.08c	ornithine aminotransferase
SPAC27D7.09c	But2 family protein	SPAC1A6.08c	sequence orphan
SPBC12C2.14c	dubious	SPAC1556.01c	DNA repair protein Rad50
SPAPB1A10.05	sequence orphan	SPBC146.07	U2AF large subunit (U2AF-59)
SPCC830.07c	DNAJ domain protein Psi1	SPAPB1E7.10	DNA-directed RNA polymerase III complex subunit Rpc17
SPCC736.05	wtf element Wtf7	SPAC18B11.05	pig-V (predicted)
SPAC1348.05	membrane transporter	SPCC1919.15	ubiquitin-protein ligase E3 Brl1
SPBC21C3.08c	ornithine aminotransferase	SPAC6G10.12c	transcription factor Ace2
SPAC2E1P3.05c	fungal cellulose binding domain protein	SPBC26H8.04c	DEP domain protein
SPAC11D3.13	ThiJ domain protein	SPAC27D7.03c	RNA-binding protein involved in meiosis Mei2
SPAC4G9.19	DNAJ domain protein DNAJB family	SPBC1685.11	RecA family ATPase Rlp1
SPAC30D11.02c	sequence orphan	SPCC550.12	actin-like protein Arp6
SPCC338.12	subtilisin cleaved region related protein (predicted)	SPCC1450.03	ribonucleoprotein (RNP) complex (predicted)
SPCC1827.03c	acetyl-CoA ligase (predicted)	SPAC57A7.06	U3 snoRNP protein Utp14 (predicted)
SPAC2F3.05c	xylose and arabinose reductase (predicted)	SPAC750.07c	S. pombe specific GPI anchored protein family 1
SPBC337.09	Erg28 protein	SPBC16A3.01	septin Spn3

SPBC651.12c	sequence orphan
SPBC106.02c	sulfiredoxin
SPBC428.18	replication licensing factor Cdt1
SPCC825.04c	N-acetyltransferase (predicted)
SPAC343.12	conserved fungal protein
SPBC1921.03c	mRNA export receptor Mex67
SPBC428.04	sequence orphan
SPBC83.18c	C2 domain protein Fic1
SPCC663.08c	short chain dehydrogenase (predicted)
SPBC21H7.06c	inositol metabolism protein Opi10 (predicted)
SPCC70.10	sequence orphan
SPAC823.15	minor serine/threonine protein phosphatase Ppa1
SPBC3B9.18c	V-type ATPase subunit F (predicted)
SPAC8E11.10	sorbose reductase (predicted)
SPBC1289.14	adducin
SPCC1223.03c	glycerol-3-phosphate dehydrogenase Gut2
SPAC2H10.01	transcription factor, zf-fungal binuclear cluster type (predicted)
SPAC6B12.07c	ubiquitin-protein ligase E3 (predicted)
SPAC922.04	sequence orphan
SPCC24B10.02c	NAD/NADH kinase (predicted)
SPAC24C9.08	vacuolar carboxypeptidase (predicted)
SPAC32A11.01	conserved fungal protein
SPBC3B9.06c	autophagy associated protein Apg3 (predicted)
SPBC3B9.01	Hsp70 nucleotide exchange factor (predicted)
SPAC57A7.05	conserved protein (fungal and plant)
SPBC29A10.11c	guanyl-nucleotide exchange factor Vps902 (predicted)
SPAC31A2.04c	20S proteasome component beta 4 (predicted)
SPAC977.11	CRCB domain protein
SPAC29A4.20	elongator complex, histone acetyltransferase subunit Elp3 (predicted)
SPCC13B11.04c	glutathione-dependent formaldehyde dehydrogenase (predicted)
SPCC663.06c	short chain dehydrogenase (predicted)
SPBC1348.04	methyltransferase (predicted)
SPAC1782.04	mitochondrial mRNA processing protein Cox24 (predicted)
SPAC890.07c	type I protein arginine N-methyltransferase Rmt1
SPAC19D5.01	tyrosine phosphatase Pyp2
SPCC70.09c	conserved fungal protein
SPAC22H12.01c	sequence orphan
SPAC1F8.06	Sim4 and Mal2 associated (4 and 2 associated) protein 5
SPBC4F6.17c	mitochondrial heatshock protein Hsp78 (predicted)
SPBC20F10.10	cyclin pho85 family
SPBC725.16	MBF transcription factor complex subunit Res1
SPAC2C4.17c	MS ion channel protein 2
SPBC32F12.09	CDK inhibitor Rum1
SPAC30D11.01c	alpha-glucosidase (predicted)
SPBC119.03	human COMT homolog 1
SPBC215.01	GTPase activating protein (predicted)
SPBC3H7.09	palmitoyltransferase (predicted)
SPBC3H7.02	sulfate transporter (predicted)
SPAC5H10.10	NADPH dehydrogenase (predicted)
SPBC1734.11	DNAJ domain protein Mas5 (predicted)
SPAC5D6.08c	meiosis II protein Mes1
SPBC13E7.09	verprolin
SPAC1687.22c	RNA-binding protein Puf3 (predicted)
SPAC977.02	S. pombe specific 5Tm protein family
SPAC19A8.02	pleckstrin homology domain protein

SPBC1718.02	linear element associated protein Hop1
SPAC31G5.04	homoisocitrate dehydrogenase
SPCC736.03c	mitochondrial phenylalanyl-tRNA synthetase
SPAC10F6.15	S. pombe specific UPF0300 family protein 1
SPCC290.02	DNA-directed RNA polymerase III complex subunit Rpc34

**2 fold downΔ gcn5 Δmst2 KCl**	**annotation**
SPAC750.05c	S. pombe specific 5Tm protein family
SPAC977.01	S. pombe specific 5Tm protein family
SPBC26H8.11c	conserved fungal protein
SPBPB2B2.19c	S. pombe specific 5Tm protein family

**2 fold downΔ gcn5 Δmst2 KCl**	**annotation**
SPCC569.05c	spermidine family transporter (predicted)
SPAC11D3.02c	ELLA family acetyltransferase (predicted)
SPBC12D12.07c	mitochondrial thioredoxin Trx2
SPBC17D11.06	DNA primase large subunit Spp2

SPBC11C11.01	RNA-binding protein	SPAC56E4.07	N-acetyltransferase (predicted)
SPAC2E1P3.05c	fungal cellulose binding domain protein	SPBC1778.01c	zuotin (predicted)
SPCC584.16c	sequence orphan	SPAC17A5.01	peroxin-6 (predicted)
SPBC1348.02	S. pombe specific 5Tm protein family	SPBC16H5.10c	ATP-dependent RNA helicase Prp43
SPAC17D4.01	peroxin-7 (predicted)	SPBC24C6.09c	phosphoketolase family protein (predicted)
SPAC57A7.11	WD repeat protein Mip1	SPBC3B9.07c	DNA-directed RNA polymerase I complex subunit Rpa43
SPAC1039.02	phosphoprotein phosphatase (predicted)	SPAC13C5.06c	sequence orphan
SPAC186.03	L-asparaginase (predicted)	SPAC19B12.10	human AMSH protein homolog
SPAC22A12.06c	serine hydrolase	SPBC1709.07	3-keto sterol reductase (predicted)
SPBC36.03c	spermidine family transporter (predicted)	SPBC21C3.08c	ornithine aminotransferase
SPAC17C9.16c	MFS family transmembrane transporter Mfs1	SPCC663.13c	N-acetyltransferase (predicted)
SPAC20G4.01	CCR4-Not complex subunit Caf16	SPCC1259.09c	pyruvate dehydrogenase protein x component
SPAC869.04	formamidase-like protein	SPAC17G8.06c	dihydroxy-acid dehydratase (predicted)
SPAC144.03	adenylosuccinate synthetase Ade2	SPBC215.06c	human LYHRT homolog
SPBC337.09	Erg28 protein	SPCC777.15	tRNA dihydrouridine synthase Dus4 (predicted)
SPAC6F6.11c	pyridoxine-pyridoxal-pyridoxamine kinase (predicted)	SPBC2G2.05	60S ribosomal protein L13/L16
SPAC17G8.13c	histone acetyltransferase Mst2	SPBC725.15	orotate phosphoribosyltransferase Ura5
SPBC16D10.06	ZIP zinc transporter Zrt1	SPBC1709.10c	copper chaperone Atx1
SPAC23C4.06c	methyltransferase (predicted)	SPAC6C3.09	RNase P subunit (predicted)
SPBC11B10.02c	histidinol-phosphate aminotransferase imidazole acetol phosphate transaminase His3	SPCC1795.05c	uridylate kinase
SPBPB2B2.10c	galactose-1-phosphate uridylyltransferase (predicted)	SPAP27G11.08c	sequence orphan
SPBC428.11	homocysteine synthase Met17	SPAC25G10.05c	ATP phosphoribosyltransferase
SPBC3B9.18c	V-type ATPase subunit F (predicted)	SPAC343.05	V-type ATPase subunit A
SPAC750.02c	membrane transporter	SPAC2F3.11	exopolyphosphatase (predicted)
SPBC651.12c	sequence orphan	SPBC1271.12	oxysterol binding protein (predicted)
SPAC1A6.10	Moeb/ThiF domain	SPAC2E1P5.04c	geranylgeranyltransferase I beta subunit Cwg2
SPBC3H7.05c	sequence orphan	SPBPB2B2.16c	MFS family membrane transporter (predicted)
SPAC1786.02	phospholipase (predicted)	SPAC23H3.05c	Set1C complex subunit Swd1
SPBPB2B2.18	sequence orphan	SPAC56F8.05c	BAR domain protein (predicted)
SPBPB2B2.09c	2-dehydropantoate 2-reductase (predicted)	SPBC646.07c	enoyl reductase
SPBC3H7.11	actin binding methyltransferase (predicted)	SPCC4G3.02	bis(5'-nucleosidyl)-tetraphosphatase
SPBC24C6.04	delta-1-pyrroline-5-carboxylate dehydrogenase	SPBC577.12	endoribonuclease (predicted)
SPBC1105.05	glucan 1,3-beta-glucosidase I/II precursor	SPBC26H8.03	phosphatidylethanolamine N-methyltransferase Cho2
SPBC32F12.12c	Golgi membrane protein involved in vesicle-medated transport (predicted)	SPBC106.11c	phospholipase A2, PAF family homolog
SPCC1827.03c	acetyl-CoA ligase (predicted)	SPAC4F10.15c	WASp homolog
SPCC1840.07c	phosphoprotein phosphatase (predicted)	SPAC57A10.12c	dihydroorotate dehydrogenase Ura3
SPBC1271.04c	eIF-5A-deoxyhypusine synthase	SPBC359.04c	cell surface glycoprotein (prediected), DIPSY family
SPBC342.06c	RTT109 family histone lysine acetyltransferase	SPBC21B10.08c	sequence orphan
SPBC1289.14	adducin	SPBC1703.03c	armadillo repeat protein, unknown biological role
SPAP7G5.04c	aminoadipate-semialdehyde dehydrogenase	SPBC21.01	kinetochore protein Mis17
SPBC18E5.08	N-acetyltransferase (predicted)	SPBC660.12c	aminotransferase (predicted)
SPAC9.02c	N-acetyltransferase (predicted)	SPBC1A4.07c	U3 snoRNP-associated protein Sof1
SPCC4B3.18	phosphopantothenate-cysteine ligase	SPAC56F8.10	methylenetetrahydrofolate reductase Met9
SPAC16E8.04c	chorismate mutase (predicted)	SPBC18E5.05c	elongator complex subunit Iki1 (predicted)
SPBC23E6.06c	3,4-dihydroxy-2-butanone 4-phosphate synthase (predicted)	SPBC365.02c	protoheme IX farnesyltransferase
		SPCC1450.14c	ER oxidoreductin Ero1b

Up-regulated

2 fold up gcn5 KCl	annotation
SPCC736.15	protein kinase inhibitor (predicted)
SPAC6F12.10c	phosphoribosylformylglycinamidine synthase Ade3 (predicted)
SPCC13B11.01	alcohol dehydrogenase Adh1
SPCC330.08	alpha-1,2-mannosyltransferase Alg11
SPBC530.10c	mitochondrial adenine nucleotide carrier Anc1
SPAC3H8.06	inositol phosphorylceramide synthase (predicted)
SPAC644.18c	TRAPP complex subunit Bet3 (predicted)
SPCC1235.02	biotin synthase
SPAC13D6.02c	zinc finger protein Byr3
SPAC27F1.02c	tropomyosin
SPAC23C11.11	serine/threonine protein kinase Cka1
SPBC1347.06c	serine/threonine protein kinase Cki1
SPCC188.03	condensin, non-SMCsubunit Cnd3
SPMIT.11	cytochrome c oxidase 2
SPCC191.07	cytochrome c (predicted)
SPBP8B7.16c	ATP-dependent RNA helicase Dbp2
SPBC20F10.01	snoRNP pseudouridylase complex protein Gar1
SPAC140.02	nucleolar protein required for rRNA processing
SPCC736.04c	alpha-1,2-galactosyltransferase Gma12
SPBC354.12	glyceraldehyde 3-phosphate dehydrogenase Gpd3
SPBC725.09c	BAR adaptor protein Hob3
SPAC12G12.04	mitochondrial heat shock protein Hsp60/Mcp60
SPAP8A3.04c	heat shock protein Hsp9
SPAC24H6.04	hexokinase 1
SPAC11H11.04	pheromone p-factor receptor
SPAC10F6.12c	protein-S isoprenylcysteine O-methyltransferase Mam4
SPAC27D7.03c	RNA-binding protein involved in meiosis Mei2
SPAC31G5.12c	repressor of RNA polymerase III Maf1
SPAC31G5.11	cAMP-independent regulatory protein Pac2
SPAC21E11.03c	transcription factor Pcr1
SPBC14F5.04c	phosphoglycerate kinase Pgk1 (predicted)
SPBP22H7.05c	ATPase with bromodomain protein
SPCC126.02c	Ku domain protein Pku70
SPBC365.06	SUMO
SPAC1002.13c	beta-glucosidase Psu1 (predicted)
SPAC4H3.10c	pyruvate kinase (predicted)
SPBC17G9.11c	pyruvate carboxylase
SPBC28F2.12	DNA-directed RNA polymerase II large subunit
SPCC1020.04c	DNA-directed RNA polymerase I, II and III subunit Rpb6
SPAC22E12.13c	60S ribosomal protein L24-3 (L30)
SPBC29A3.04	60S ribosomal protein L7a (L8)
SPBC119.01	19S proteasome regulatory subunit Rpn3
SPCC18.14c	60S acidic ribosomal protein Rpp0 (predicted)
SPBC14F5.05c	S-adenosylmethionine synthetase
SPBC31F10.06c	ADP-ribosylation factor Sar1
SPAC6G9.11	SNAP receptor, synaptobrevin family
SPAC167.03c	U4/U6 x U5 tri-snRNP complex subunit Snu66 (predicted)
SPAC821.10c	superoxide dismutase Sod1
SPAC1002.02	nucleoporin Pom34 (predicted)
SPAC1002.17c	uracil phosphoribosyltransferase (predicted)
SPAC1002.19	GTP cyclohydrolase II (predicted)
SPAC1093.05	ATP-dependent RNA helicase Hca4 (predicted)
SPAC10F6.16	endosulphine family protein
SPAC11E3.07	V-type ATPase subunit E
SPAC11G7.03	isocitrate dehydrogenase (NAD+) subunit 1 Idh1
SPAC12G12.07c	conserved fungal protein
SPAC14C4.12c	clr6 L associated factor 1 Laf1
SPAC16.05c	transcription factor Sfp1 (predicted)
SPAC16E8.06c	RNA-binding protein Nop12
SPAC1705.03c	conserved fungal family
SPAC17H9.05	rRNA processing protein Ebp2

2 fold up mst2	annotation
SPAC1002.17c	uracil phosphoribosyltransferase (predicted)
SPAC9E9.12c	ABC transporter Ybt1
SPCC13B11.01	alcohol dehydrogenase Adh1
SPBC337.16	phosphatidyl-N-methylethanolamine N-methyltransferase (predicted)
SPCC191.07	cytochrome c (predicted)
SPAC8E11.03c	RecA family ATPase Dmc1
SPBC1198.14c	fructose-1,6-bisphosphatase Fbp1
SPBC646.12c	GTPase activating protein Gap1
SPAC144.14	kinesin-like protein Klp8
SPBC1921.03c	mRNA export receptor Mex67
SPAC3C7.14c	ubiquitinated histone-like protein Uhp1
SPCC1739.12	serine/threonine protein phosphatase Ppe1
SPBC119.13c	U4/U6 x U5 tri-snRNP complex subunit Prp31
SPAC343.12	conserved fungal protein
SPCC4G3.10c	DNA repair protein Rhp42
SPBC14F5.05c	S-adenosylmethionine synthetase
SPBC31F10.06c	ADP-ribosylation factor Sar1
SPAC1B9.02c	serine/threonine protein kinase Sck1
SPAC1002.18	DUF1688 family protein
SPAC1002.19	GTP cyclohydrolase II (predicted)
SPAC10F6.16	endosulphine family protein
SPAC17A5.18c	meiotic recombination protein Rec25
SPAC18B11.04	related to neuronal calcium sensor Ncs1
SPAC19G12.16c	conserved fungal protein
SPAC1A6.03c	phospholipase (predicted)
SPAC22E12.06c	alpha-1,2-galactosyltransferase Gmh3
SPAC22G7.08	serine/threonine protein kinase Ppk8 (predicted)
SPAC23A1.14c	uncharacterised trans-sulfuration enzyme (predicted)
SPAC24C9.14	ubiquitin-specific protease (predicted)
SPAC25A8.02	sequence orphan
SPAC25H1.02	Jmj1 protein
SPAC27D7.11c	But2 family protein
SPAC2F3.05c	xylose and arabinose reductase (predicted)
SPAC31A2.12	arrestin/PY protein 1
SPAC3C7.02c	protein kinase inhibitor (predicted)
SPAC3C7.13c	glucose-6-phosphate 1-dehydrogenase (predicted)
SPAC3G6.03c	Maf-like protein
SPAC4G8.11c	F1-F0 ATPase assembly protein (predicted)
SPAC4G9.19	DNAJ domain protein DNAJB family
SPAC513.06c	dihydrodiol dehydrogenase (predicted)
SPAC521.03	short chain dehydrogenase (predicted)
SPAC57A10.09c	High-mobility group non-histone chromatin protein
SPAC167.06c	sequence orphan
SPAC630.08c	C-4 methylsterol oxidase (predicted)
SPAC688.04c	glutathione S-transferase Gst3
SPAC806.04c	DUF89 family protein
SPAC869.10c	proline specific permease (predicted)
SPAP7G5.06	amino acid permease, unknown 4
SPAPB24D3.07c	sequence orphan
SPAPB2B4.02	monothiol glutaredoxin Grx5
SPAC1782.01	proteasome component
SPBC106.16	20S proteasome component alpha 4 Pre6
SPBC1198.13c	transcription factor TFIIF complex beta subunit Tfg2 (predicted)
SPBC1347.11	Stress Responsive Orphan 1
SPBC14F5.10c	ubiquitin-protein ligase E3 (predicted)
SPBC16E9.11c	HECT-type ubiquitin-protein ligase Pub3
SPBC19G7.06	MADS-box transcription factor Mbx1
SPBC211.05	splicing factor 3B
SPBC21D10.06c	cell surface agglutination protein Map4
SPBC21H7.06c	inositol metabolism protein Opi10 (predicted)
SPBC26H8.13c	sequence orphan

SPAC18G6.01c	calchone related protein family
SPAC19D5.09c	retrotransposable element
SPAC19G12.16c	conserved fungal protein
SPAC1A6.03c	phospholipase (predicted)
SPAC1A6.04c	phospholipase B homolog Plb1
SPAC1B3.06c	UbiE family methyltransferase (predicted)
SPAC1F5.03c	FAD-dependent oxidoreductase (predicted)
SPAC227.11c	sensor for misfolded ER glycoproteins Yos9 (predicted)
SPAC227.17c	conserved protein (fungal and plant)
SPAC22A12.06c	serine hydrolase
SPAC22A12.10	diacylglycerol cholinephosphotranferase/ diacylglycerol ethanolaminesphotranferase (predicted)
SPAC22E12.03c	THIJ/PFPI family peptidase (predicted)
SPAC22G7.07c	mRNA (N6-adenosine)-methyltransferase (predicted)
SPAC23A1.17	WIP homolog
SPAC23D3.12	inorganic phosphate transporter (predicted)
SPAC23H4.17c	cyclin-dependent protein Srb mediator subunit kinase Srb10
SPAC25B8.12c	nucleotide-sugar phosphatase (predicted)
SPAC26A3.08	Sm snRNP core protein Smb1
SPAC26A3.16	UBA domain protein Dph1
SPAC26F1.02	pinin homologue
SPAC27F1.06c	FKBP-type peptidyl-prolyl cis-trans isomerase (predicted)
SPAC29A4.13	urease accessory protein UreF
SPAC2C4.13	V-type ATPase subunit c''
SPAC29E6.02	U4/U6 x U5 tri-snRNP complex subunit Prp3 (predicted)
SPAC30C2.04	cofactor for methionyl-and glutamyl-tRNA synthetases (predicted)
SPAC30D11.03	ATP-dependent RNA helicase Ddx27/Drs1 (predicted)
SPAC30D11.11	Haemolysin-III family protein
SPAC31G5.10	Myb family protein Eta2
SPAC323.02c	20S proteasome component alpha 5, Pup2 (predicted)
SPAC3A12.16c	TIM23 translocase complex subunit Tim17
SPAC4F8.06	mitochondrial ribosomal protein subunit S12 (predicted)
SPAC513.04	sequence orphan
SPAC56F8.15	dubious
SPAC57A10.09c	High-mobility group non-histone chromatin protein
SPAC607.06c	metallopeptidase
SPAC644.08	methionine salvage haloacid dehalogenase-like hydrolase (predicted)
SPAC644.17c	mitochondrial ribosomal protein subunit L9 (predicted)
SPAC683.02c	zf-CCHC type zinc finger protein
SPAC6G9.08	ubiquitin C-terminal hydrolase Ubp6
SPAC732.01	V-type ATPase proteolipid subunit
SPAC806.04c	DUF89 family protein
SPAC9.09	homocysteine methyltransferase
SPAC9E9.09c	aldehyde dehydrogenase (predicted)
SPAP27G11.09c	GTP cyclohydrolase (predicted)
SPAP7G5.06	amino acid permease, unknown 4
SPAP8A3.05	ski complex interacting GTPase (predicted)
SPAPB1E7.07	glutamate synthase Glt1 (predicted)
SPAPB2B4.02	monothiol glutaredoxin Grx5
SPAPB2B4.07	ubiquitin family protein, human UBTD1 homolog
SPBC106.16	20S proteasome component alpha 4 Pre6
SPBC1105.14	transcription factor Rsv2
SPBC119.09c	ORMDL family protein
SPBC119.10	asparagine synthetase
SPBC1198.07c	mannan endo-1,6-alpha-mannosidase (predicted)
SPBC1198.13c	transcription factor TFIIF complex beta subunit Tfg2 (predicted)
SPBC11C11.05	KRE9 family cell wall biosynthesis protein (predicted)
SPBC11G11.03	ribosome assembly protein (predicted)
SPBC1347.11	Stress Responsive Orphan 1
SPBC13E7.04	F1-ATPase delta subunit (predicted)
SPBC146.02	sequence orphan
SPBC14F5.10c	ubiquitin-protein ligase E3 (predicted)
SPBC16A3.08c	serpine1 related protein (predicted)
SPBC16E9.11c	HECT-type ubiquitin-protein ligase Pub3
SPBC16H5.05c	cyclophilin family peptidyl-prolyl cis-trans isomerase

SPBC530.07c	phosphomethylpyrimidine kinase (predicted)
SPBC582.08	alanine aminotransferase (predicted)
SPBC725.03	conserved fungal protein
SPBC839.16	C1-5,6,7,8-tetrahydrofolate (THF) synthase
SPBP19A11.02c	sequence orphan
SPBP23A10.14c	RNA polymerase II transcription elongation factor SpELL
SPCC1322.10	conserved fungal protein
SPCC16A11.04	sorting nexin Snx12 (predicted)
SPCC16A11.15c	sequence orphan
SPCC24B10.06	sequence orphan
SPCC285.04	transthyretin (predicted)
SPCC4B3.06c	NADPH-dependent FMN reductase (predicted)
SPCC584.11c	Svf1 family protein Svf1
SPCC663.09c	short chain dehydrogenase (predicted)
SPCC736.10c	mitochondrial ribosomal protein subunit S8
SPCC757.03c	ThiJ domain protein
SPCC757.11c	membrane transporter
SPCC794.15	sequence orphan
SPCC965.06	potassium channel subunit (predicted)
SPCPB16A4.06c	sequence orphan
SPCC825.03c	SNARE Psy1
SPAC1250.03	ubiquitin conjugating enzyme Ubc14
SPCC330.05c	orotidine 5'-phosphate decarboxylase Ura4
SPBC649.04	UV-induced protein Uvi15
SPAC9E9.07c	GTPase Ypt2

	Cyp7
SPBC1703.13c	mitochondrial inorganic phosphate transporter (predicted)
SPBC1709.15c	cleavage factor two Cft2/polyadenylation factor CPSF-73 (predicted)
SPBC1773.01	striatin homolog
SPBC17D11.08	WD repeat protein, human WDR68 family
SPBC1E8.04	retrotransposable element: pseudo
SPBC21D10.09c	ubiquitin-protein ligase E3 (predicted)
SPBC25H2.15	programmed cell death protein homolog
SPBC29A10.06c	conserved fungal protein
SPBC29A10.09c	CAF1 family ribonuclease
SPBC2A9.05c	DUF846 family protein
SPBC2G2.12	mitochondrial and cytoplasmic histidine-tRNA ligase Hrs1
SPBC2G5.05	transketolase (predicted)
SPBC30B4.08	double-strand siRNA ribonuclease
SPBC27B12.01c	Mdm10/Mdm12/Mmm1 complex subunit Mmm1 (predicted)
SPBC359.03c	amino acid permease, unknown 8
SPBC359.06	adducin
SPBC36B7.08c	nucleosome assembly protein (predicted)
SPBC3H7.06c	F-box protein Pof9
SPBC3H7.07c	phosphoserine phosphatase (predicted)
SPBC543.02c	DNAJ/TPR domain protein DNAJC7 family
SPBC577.11	sequence orphan
SPBC660.05	conserved fungal protein
SPBC725.13c	GINS complex subunit Psf2
SPBC776.03	homoserine dehydrogenase (predicted)
SPBC83.13	mitochondrial tricarboxylic acid transporter
SPBC839.10	U1 snRNP-associated protein Usp107
SPBP23A10.03c	ACN9 family mitochondrial protein
SPBP23A10.15c	mitochondrial processing peptidase complex beta subunit Qcr1
SPBP23A10.16	TIM22 inner membrane protein import complex anchor subunit Tim18
SPBP8B7.05c	carbonic anhydrase (predicted)
SPBP8B7.25	cyclophilin family peptidyl-prolyl cis-trans isomerase Cyp4
SPCC1281.06c	acyl-coA desaturase (predicted)
SPCC1393.08	transcription factor, zf-GATA type (predicted)
SPCC1393.09c	RWD domain
SPCC1393.12	sequence orphan
SPCC1393.13	DUF89 family protein
SPCC1494.11c	retrotransposable element: pseudo
SPCC1620.09c	transcription factor TFIIF complex alpha subunit Tfg1 (predicted)
SPCC1682.04	centrin
SPCC16C4.08c	Shk1 kinase binding protein 15
SPCC1739.09c	cytochrome c oxidase subunit VIa (predicted)
SPCC18.05c	notchless-like protein
SPCC1827.06c	aspartate semialdehyde dehydrogenase (predicted)
SPCC191.08	DUF1715 family protein
SPCC1919.07	sequence orphan
SPCC31H12.03c	RNA binding protein (predicted)
SPCC1235.01	sequence orphan
SPCC320.11c	RNA-binding protein
SPCC338.10c	cytochrome c oxidase subunit V
SPCC364.06	nucleosome assembly protein Nap1
SPCC417.08	translation elongation factor eEF3
SPCC550.11	karyopherin
SPCC550.15c	ribosome biogenesis protein (predicted)
SPCC553.12c	conserved fungal protein
SPCC584.11c	Svf1 family protein Svf1
SPCC584.16c	sequence orphan
SPCC594.04c	steroid oxidoreductase superfamily protein
SPCC594.07c	sequence orphan
SPCC11E10.01	cystathionine beta-lyase (predicted)
SPCC61.01c	siderophore-iron transporter Str2
SPCC63.14	conserved fungal protein
SPCC736.10c	mitochondrial ribosomal protein subunit S8

SPCC737.02c	ubiquinol-cytochrome-c reductase complex subunit 6
SPCC790.03	rhomboid family protease
SPCC794.04c	membrane transporter
SPCC794.11c	ENTH/VHS domain protein Ent3
SPCC970.10c	ubiquitin-protein ligase E3 Brl2
SPCP1E11.05c	acyl-coA-sterol acyltransferase (predicted)
SPCP1E11.08	ribosome biogenesis protein Nsa2 (predicted)
SPCP1E11.11	Puf family RNA-binding protein
SPCPB16A4.05c	urease accessory protein UREG (predicted)
SPCPJ732.02c	xylulose kinase (predicted)
SPBC21C3.18	serine/threonine protein kinase Spo4
SPCC188.06c	signal recognition particle subunit Srp54
SPAC4H3.05	ATP-dependent DNA helicase, UvrD subfamily
SPCC825.03c	SNARE Psy1
SPBC776.09	ATP-dependent RNA helicase Ste13
SPAC1565.04c	adaptor protein Ste4
SPCC5E4.03c	SAGA complex subunit/TATA-binding protein associated factor/transcription factor TFIID complex subunit Taf5
SPAC29E6.08	TATA-binding protein (TBP)
SPAC29A4.02c	translation elongation factor EF-1 gamma subunit
SPCC1450.04	translation elongation factor EF-1 beta subunit (eEF1B)
SPBC25H2.07	translation initiation factor eIF1A
SPBC17G9.09	translation initiation factor eIF2 gamma subunit
SPAC4D7.05	translation initiation factor eIF3i
SPBC1709.18	translation initiation factor eIF4E 4F complex subunit
SPCC1919.09	translation initiation factor eIF6
SPCC24B10.21	triosephosphate isomerase
SPBC337.08c	ubiquitin, ubi4
SPCC285.07c	wtf element Wtf18
SPBC1289.17	retrotransposable element
SPBC1E8.04	retrotransposable element
SPAC13D1.01c	retrotransposable element
SPAC26A3.13c	retrotransposable element
SPAC167.08	retrotransposable element
SPCC1020.14	retrotransposable element
SPAPB15E9.03c	retrotransposable element
SPAC27E2.08	retrotransposable element
SPAC9.04	retrotransposable element
SPBC9B6.02c	retrotransposable element

2 fold up-regulated in Δgcn5 Δmst2 after 60 min,, KCL	annotation
SPCC330.05c	orotidine 5'-phosphate decarboxylase Ura4
SPCC1739.08c	short chain dehydrogenase (predicted)
SPBC359.06	adducin
SPBC1198.14c	fructose-1,6-bisphosphatase Fbp1
SPAC3G9.11c	pyruvate decarboxylase (predicted)
SPCC794.01c	glucose-6-phosphate 1-dehydrogenase (predicted)
SPCC13B11.01	alcohol dehydrogenase Adh1
SPAC13F5.03c	mitochondrial glycerol dehydrogenase Gld1
SPACUNK4.10	hydroxyacid dehydrogenase (predicted)
SPAC1002.19	GTP cyclohydrolase II (predicted)
SPAC22H10.13	metallothionein Zym1
SPBC13A2.04c	PTR family peptide transporter
SPBC19C2.05	serine/threonine protein kinase Ran1
SPBC1347.11	Stress Responsive Orphan 1
SPAC31G5.09c	MAP kinase Spk1
SPCC757.03c	ThiJ domain protein
SPAC1039.09	amino acid permease Isp5
SPBC3E7.13c	Splicing factor, SYF2 family

2 fold up-regulated in Δgcn5 Δmst2	annotation
SPAC5D6.04	auxin family transmembrane transporter (predicted)
SPBC800.07c	mitochondrial translation elongation factor EF-Ts Tsf1
SPBC18H10.05	WD repeat protein, human WDR44 family
SPBC16G5.05c	MSP domain
SPAPB24D3.10c	alpha-glucosidase Agl1
SPAC1327.01c	transcription factor, zf-fungal binuclear cluster type (predicted)
SPAPB24D3.07c	sequence orphan
SPAC19G12.08	sphingosine hydroxylase (predicted)
SPCC306.10	wtf element Wtf8, pseudo
SPAPB2B4.03	cyclin Cig2
SPBC1271.05c	zf-AN1 type zinc finger protein
SPCP31B10.06	C2 domain protein
SPAC29B12.04	pyridoxine biosynthesis protein
SPCC663.06c	short chain dehydrogenase (predicted)
SPAC22F8.04	triose phosphate transporter (predicted)
SPCC1259.03	DNA-directed RNA polymerase complex I subunit Rpa12
SPCC338.18	sequence orphan
SPBC646.12c	GTPase activating protein Gap1

SPCC16A11.15c	sequence orphan	SPAC11G7.03	isocitrate dehydrogenase (NAD+) subunit 1 Idh1
SPAC27D7.03c	RNA-binding protein involved in meiosis Mei2	SPAC343.12	conserved fungal protein
SPAC22F3.12c	regulator of G-protein signaling Rgs1	SPAC3G9.04	phosphoric ester hydrolase Ssu72 (predicted)
SPAC977.16c	dihydroxyacetone kinase Dak2	SPAPB24D3.08c	NADP-dependent oxidoreductase (predicted)
SPAC15E1.02c	DUF1761 family protein	SPBC1711.02	mating-type m-specific polypeptide mc
SPCC1393.12	sequence orphan	SPBC1683.08	hexose transporter Ght4
SPCC1020.02	kinetochore protein Spc7	SPCPB16A4.03c	IMPcyclohydrolase/phosphoribosylaminoimidazolecarbox amideformyltransferase
SPAC11H11.04	pheromone p-factor receptor	SPAP8A3.13c	Vid24 family protein
SPAC1F5.09c	PAK-related kinase Shk2	SPAC2F3.10	GARP complex subunit Vps54 (predicted)
SPCC965.06	potassium channel subunit (predicted)	SPAC56F8.16	transcription factor Esc1 (predicted)
SPBC725.10	tspO homolog/ peripheral benzodiazepine receptor homolog, involved in the transport cytoplas/mitochondrial of haem (predicted)	SPAC26H5.08c	glucan 1,3-beta-glucosidase Bgl2
SPBC32C12.02	transcription factor Ste11	SPAC1635.01	voltage-dependent anion-selective channel
SPAC26H5.09c	GFO/IDH/MocA family oxidoreductase	SPAC23D3.12	inorganic phosphate transporter (predicted)
SPAC1A6.04c	phospholipase B homolog Plb1	SPBC215.11c	aldo/keto reductase, unknown biological role
SPAC19G12.16c	conserved fungal protein	SPBC1683.01	inorganic phosphate transporter (predicted)
SPAC11D3.01c	conserved fungal protein	SPAC57A10.09c	High-mobility group non-histone chromatin protein
SPAC19G12.09	NADH/NADPH dependent indole-3-acetaldehyde reductase AKR3C2	SPBC800.10c	EPS15 repeat family actin cortical patch component (predicted)
SPAC26F1.14c	apoptosis-inducing factor homolog Aif1	SPAC16E8.02	DUF962 family protein
SPCC965.07c	glutathione S-transferase Gst2	SPCC1919.06c	wtf element
SPBC16E9.11c	HECT-type ubiquitin-protein ligase Pub3	SPAC20G8.02	mitochondrial phospholipase (predicted)
SPCC584.11c	Svf1 family protein Svf1	SPBC24C6.06	G-protein alpha subunit
SPBC19F8.07	cyclin-dependent kinase activating kinase Crk1	SPBC1778.04	Spo4-Spo6 kinase complex regulatory subunit Spo6
SPAC2F3.05c	xylose and arabinose reductase (predicted)	SPBC1711.02	mating-type m-specific polypeptide mc
SPBC19C2.04c	ubiquitin C-terminal hydrolase Ubp11	SPAC4A8.04	vacuolar serine protease Isp6
SPAC3C7.14c	ubiquitinated histone-like protein Uhp1	SPBC3H7.02	sulfate transporter (predicted)
SPAC8F11.10c	pyruvyltransferase Pvg1	SPAP14E8.02	homolog of S. cerevisiae Tos4
SPBC29A10.14	meiotic cohesin complex subunit Rec8	SPCC1620.04c	Cdc20/Fizzy family WD repeat protein
SPBC56F2.06	sequence orphan	SPAC637.03	conserved fungal protein
SPCC737.04	S. pombe specific UPF0300 family protein 6	SPAC14C4.07	membrane transporter
SPAC21E11.04	L-azetidine-2-carboxylic acid acetyltransferase	SPBC2G2.17c	beta-glucosidase Psu2 (predicted)
SPCC1442.01	guanyl-nucleotide exchange factor Ste6	SPCC594.02c	conserved fungal protein
SPBC1683.06c	uridine ribohydrolase (predicted)	SPAC1D4.14	THO complex subunit Tho2 (predicted)
SPAC14C4.01c	DUF1770 family protein	SPBP4H10.10	rhomboid family protease
SPAC4H3.03c	glucan 1,4-alpha-glucosidase (predicted)	SPAC12B10.14c	serine/threonine protein kinase Ppk2 (predicted)
SPAC521.03	short chain dehydrogenase (predicted)	SPAC9E9.03	3-isopropylmalate dehydratase Leu2 (predicted)
SPBC19G7.06	MADS-box transcription factor Mbx1	SPCC895.08c	conserved fungal protein
SPCC663.08c	short chain dehydrogenase (predicted)	SPAC869.10c	proline specific permease (predicted)
SPAPB8E5.03	malic acid transport protein Mae1	SPBC29A10.03c	chromatin remodeling complex subunit Rlf2 (predicted)
SPCC70.08c	methyltransferase (predicted)	SPAC30D11.02c	sequence orphan
SPCC548.07c	hexose transporter Ght1	SPCC285.11	UBA/UAS domain protein Ucp10
SPAC4G9.12	gluconokinase	SPAC31G5.10	Myb family protein Eta2
SPBC32H8.02c	NEDD8 protease Nep2	SPAC19G12.10c	vacuolar carboxypeptidase Y
SPBC725.11c	CCAAT-binding factor complex subunit Php2	SPBC119.03	human COMT homolog 1
SPBC725.03	conserved fungal protein	SPAC513.02	phosphoglycerate mutase family
SPAPB1A10.14	F-box protein	SPCC191.01	sequence orphan
SPAC31G5.08	uroporphyrinogen-III synthase Ups1	SPBC902.05c	isocitrate dehydrogenase (NAD+) subunit 2

SPCC306.08c	malate dehydrogenase	SPAC977.17	MIP water channel (predicted)
SPAC11D3.18c	nicotinic acid plasma membrane transporter (predicted)	SPBC32F12.03c	glutathione peroxidase Gpx1
SPCC594.04c	steroid oxidoreductase superfamily protein	SPBC215.10	haloacid dehalogenase-like hydrolase
SPBC23G7.10c	NADH-dependent flavin oxidoreductase (predicted)	SPAC167.05	Usp (universal stress protein) family protein, implicated in meiotic chromosome segregation
SPBP23A10.11c	conserved fungal protein	SPCC1739.06c	uroporphyrin methyltransferase (predicted)
SPAC27D7.11c	But2 family protein	SPBC8D2.18c	adenosylhomocysteinase (predicted)
SPCC191.11	beta-fructofuranosidase	SPCC16A11.04	sorting nexin Snx12 (predicted)
SPAC688.04c	glutathione S-transferase Gst3	SPAC513.06c	dihydrodiol dehydrogenase (predicted)
SPBC1921.03c	mRNA export receptor Mex67	SPAC6G9.05	coenzyme A diphosphatase (predicted)
SPBC1773.06c	alcohol dehydrogenase (predicted)	SPBC651.03c	GTPase activating protein Gyp10
SPAC922.03	1-aminocyclopropane-1-carboxylate deaminase (predicted)	SPCC645.06c	RhoGEF Rgf3
SPCC1739.01	zf-CCCH type zinc finger protein	SPAC11E3.06	MADS-box transcription factor Map1
SPAC227.13c	mitochondrial iron-sulfur cluster assembly scaffold protein Isu1	SPBC19C2.02	DNA methyltransferase homolog
SPAC25G10.09c	actin cortical patch component, with EF hand and WH2 motif Panl (predicted)	SPBC543.07	MAP kinase kinase Pek1
SPBC1718.07c	CCCH tandem zinc finger protein, human Tristetraprolin homolog Zfs1	SPAC4D7.02c	glycerophosphoryl diester phosphodiesterase (predicted)
SPCC1322.10	conserved fungal protein	SPBC216.03	conserved fungal protein
SPBC25B2.03	zf-C3HC4 type zinc finger	SPAC167.06c	sequence orphan
SPAC806.04c	DUF89 family protein	SPAC19A8.12	mRNA decapping complex subunit Dcp2
SPAC1B9.02c	serine/threonine protein kinase Sck1	SPCC757.02c	epimarase (predicted)
SPAC22A12.11	dihydroxyacetone kinase Dak1	SPBC13E7.03c	RNA hairpin binding protein (predicted)
SPAC26F1.04c	enoyl-[acyl-carrier protein] reductase	SPCC1902.01	transcription factor Gaf1
SPBPB7E8.02	PSP1 family protein	SPBC337.16	phosphatidyl-N-methylethanolamine N-methyltransferase (predicted)
SPBC19G7.13	DNA binding factor Trf1	SPAC5D6.13	Golgi phosphoprotein 3 family
SPBC29A10.16c	cytochrome b5 (predicted)	SPBC1718.01	F-box/WD repeat protein Pop1
SPAC328.07c	heavy metal ion homeostasis protein (predicted)	SPCC794.07	dihydrolipoamide S-acetyltransferase E2 (predicted)
SPAC23E2.03c	meiotic suppressor protein Ste7	SPAC20H4.11c	Rho family GTPase Rho5
SPBC14F5.10c	ubiquitin-protein ligase E3 (predicted)	SPCC1620.02	wtf element Wtf23
SPBC1685.05	serine protease (predicted)	SPCC663.09c	short chain dehydrogenase (predicted)
SPBC21C3.18	serine/threonine protein kinase Spo4	SPBC1D7.02c	transcription factor Scr1
SPAC3H1.11	transcription factor Hsr1	SPBP4H10.12	conserved protein (fungal and bacterial)
SPAC1F7.07c	iron permease Fip1		
SPAC19E9.03	cyclin Pas1		

This table lists the down and up-regulated genes of mutant HATs using an Eurogentech microarray.
