# Supplementary material for: Expression profiling of S. pombe acetyltransferase mutants identifies redundant pathways of gene regulation
Source: BMC Genomics. 2010 Jan 22;11:59. doi: 10.1186/1471-2164-11-59 (PMC2823694; doi:10.1186/1471-2164-11-59)
Supplement: Additional file 5 — Differentially expressed gene (1.7 fold) in HAT mutants. This table lists the differentially changed genes in the HAT mutants compared to wild-type using an Affymetrix microarray. [file 1471-2164-11-59-S5.PDF]

Additional File 5: Differentially expressed genes (1.7 fold) in HAT mutants

Downregulated

	Δ*elp3*			Δ*gcn5*			Δ*mst2*	
Gene	Log2 Change	pvalue	Gene	Log2 Change	pvalue	Gene	Log2 Change	pvalue
SPAC29A4.20	-4.98	7.31E-15	SPBC11B10.02c	-5.12	7.16E-03	SPAC186.05c	-3.77	4.00E-07
SPAC186.05c	-4.31	6.03E-08	SPAC186.05c	-4.09	1.26E-07	SPBPB2B2.06c	-3.66	6.60E-07
SPAC186.06	-3.23	5.87E-06	SPAC1952.05	-3.47	1.08E-02	SPAC186.06	-3.52	1.94E-06
SPCC794.04c	-3.13	6.59E-05	SPBPB2B2.06c	-3.26	3.11E-06	SPAC17G8.13c	-2.76	2.03E-02
SPCC191.11	-2.57	4.45E-03	SPAC186.06	-3.23	5.82E-06	SPAC1039.02	-2.26	2.58E-03
SPBC4F6.09	-2.50	3.77E-05	SPBPB2B2.01	-2.60	2.00E-05	SPBPB2B2.01	-2.23	1.12E-04
SPBPB2B2.08	-2.34	1.48E-02	SPBPB2B2.05	-2.35	1.46E-02	SPAC977.05c	-1.70	9.42E-03
SPAC27D7.03c	-2.27	1.22E-02	SPBPB2B2.08	-2.22	2.01E-02	SPAC1F8.06	-1.57	7.11E-05
SPCC1235.14	-2.22	8.76E-08	SPAC977.14c	-2.21	2.84E-02	SPBC29B5.02c	-1.36	1.32E-05
SPCC569.03	-2.18	9.22E-07	SPAC1039.02	-2.17	3.46E-03	SPCC285.05	-1.28	6.43E-03
SPAC23H3.15c	-2.16	1.25E-03	SPAC977.05c	-2.10	2.09E-03	SPBPB2B2.18	-1.25	2.65E-02
SPBC660.05	-2.08	1.75E-02	SPBPB10D8.02c	-2.02	1.47E-03	SPBC947.04	-1.24	6.60E-03
SPBC354.12	-2.06	5.60E-08	SPAC23H3.15c	-1.82	4.83E-03	SPCC1393.10	-1.22	2.17E-04
SPAC869.05c	-2.00	1.97E-03	SPAC57A10.06	-1.69	3.58E-02	SPCC584.16c	-1.20	1.05E-02
SPBC1683.08	-1.90	8.55E-03	SPCC569.05c	-1.57	6.36E-03	SPCPB1C11.01	-1.18	2.27E-03
SPAC57A10.06	-1.87	2.15E-02	SPAC3C7.14c	-1.54	1.83E-02	SPAC8E11.10	-1.15	6.06E-04
SPBC215.11c	-1.75	1.23E-03	SPAC24B11.14	-1.48	2.34E-02	SPBCPT2R1.02	-1.11	4.33E-04
SPBP4H10.10	-1.64	7.21E-03	SPBPB2B2.18	-1.28	2.35E-02	SPCC1682.09c	-1.10	6.11E-04
SPBC16A3.16	-1.62	2.87E-03	SPBPB10D8.01	-1.20	3.42E-02	SPAC1002.17c	-1.08	3.88E-02
SPAC977.05c	-1.56	1.59E-02	SPBC106.02c	-1.17	1.30E-02	SPCC965.14c	-1.06	2.34E-02
SPCC1902.02	-1.55	1.30E-04	SPBC947.04	-1.14	1.13E-02	SPAC1B3.16c	-1.05	2.17E-04
SPAC27D7.11c	-1.53	8.73E-04	SPBCPT2R1.02	-1.09	5.27E-04	SPAC1399.02	-1.05	7.38E-06
SPAC167.06c	-1.51	7.97E-04	SPAC11D3.03c	-1.06	2.67E-03	SPCC965.12	-1.03	5.83E-04
SPCC191.01	-1.51	1.11E-04	SPAC23C4.06c	-1.02	1.68E-02	SPBC317.01	-1.03	1.23E-03
SPAC18G6.12c	-1.49	1.70E-04	SPAC1F8.06	-0.99	4.64E-03	SPAC869.10c	-1.03	1.38E-02
SPAC3F10.15c	-1.49	9.46E-04	SPBC29B5.02c	-0.98	4.53E-04	SPAC1399.04c	-0.99	4.40E-02

SPAC17A5.09c	-1.46	1.02E-07	SPCC663.09c	-0.94	1.75E-02	SPAC1002.19	-0.98	2.31E-02
SPBC26H8.11c	-1.46	2.72E-05	SPCC1682.08c	-0.90	1.17E-02	SPCC576.02	-0.97	2.90E-04
SPBC29B5.02c	-1.42	8.03E-06	SPAC869.09	-0.88	4.42E-02	SPAC24H6.13	-0.96	1.58E-03
SPAC26F1.04c	-1.41	4.05E-06	SPBC1683.02	-0.87	1.97E-02	SPCC1223.13	-0.95	4.28E-02
SPBC16D10.06	-1.33	5.84E-07	SPCC965.12	-0.85	3.09E-03	SPCC1682.08c	-0.94	9.14E-03
SPAC27D7.09c	-1.32	2.41E-02	SPAC869.06c	-0.84	2.68E-02	SPBC3H7.09	-0.93	3.24E-03
SPBC8D2.09c	-1.29	5.60E-03				SPCC965.11c	-0.92	5.10E-03
SPCC320.06	-1.28	1.85E-05				SPBC36.01c	-0.91	3.53E-03
SPAC22H10.13	-1.26	2.24E-02				SPBC30B4.01c	-0.89	2.83E-02
SPAPYUG7.04c	-1.26	9.16E-04				SPBC25H2.04c	-0.84	4.97E-04
SPAC589.09	-1.26	3.20E-04				SPAC869.06c	-0.82	2.99E-02
SPBC3E7.02c	-1.26	1.67E-02				SPAC4G9.20c	-0.81	4.41E-03
SPBPB21E7.04c	-1.25	3.58E-02				SPBC359.04c	-0.80	7.80E-03
SPBC106.02c	-1.23	9.24E-03				SPBC725.12	-0.80	4.37E-02
SPCC338.18	-1.23	7.79E-03						
SPBPB7E8.02	-1.21	6.51E-03						
SPBC337.16	-1.21	5.42E-06						
SPAC20G4.03c	-1.20	2.45E-03						
SPAC22F8.05	-1.20	3.03E-02						
SPCC23B6.05c	-1.19	8.61E-04						
SPBP22H7.03	-1.17	5.35E-05						
SPAC8C9.16c	-1.17	2.29E-04						
SPAC19G12.09	-1.17	1.89E-04						
SPAC1F12.03c	-1.17	7.84E-03						
SPAC29B12.11c	-1.16	1.24E-04						
SPAC1687.14c	-1.16	7.79E-04						
SPAC11E3.06	-1.16	2.61E-02						
SPBPB2B2.06c	-1.16	2.98E-02						
SPBC365.01	-1.15	1.02E-05						
SPAC22F3.05c	-1.15	8.55E-03						
SPAC18G6.01c	-1.14	1.62E-02						

SPCC338.12	-1.12	1.07E-03
SPAC2F3.05c	-1.12	1.46E-04
SPBC725.03	-1.09	1.43E-02
SPAC1F7.07c	-1.08	4.80E-02
SPBC3B8.10c	-1.08	1.68E-03
SPAC1834.03c	-1.07	9.71E-05
SPCC663.09c	-1.05	9.10E-03
SPAC18B11.04	-1.05	2.05E-04
SPAC1565.04c	-1.05	4.55E-03
SPAC31G5.21	-1.05	7.46E-03
SPBC6B1.02	-1.04	4.82E-06
SPAC23A1.09	-1.04	1.65E-04
SPAC637.03	-1.04	2.36E-02
SPAC17G6.02c	-1.04	2.58E-05
SPCC1620.01c	-1.03	4.29E-05
SPBP8B7.08c	-1.03	5.77E-04
SPBC24C6.06	-1.03	5.47E-04
SPAC9E9.14	-1.03	2.89E-04
SPAC458.06	-1.02	1.77E-05
SPBC36.03c	-1.02	1.89E-04
SPAC25B8.01	-1.01	4.41E-03
SPBC21C3.04c	-0.98	5.78E-04
SPCC1223.13	-0.97	3.85E-02
SPBC27.05	-0.97	4.18E-03
SPBC25B2.10	-0.97	1.16E-04
SPCC18B5.09c	-0.97	4.67E-05
SPBC20F10.06	-0.96	7.36E-03
SPBC800.14c	-0.95	1.16E-03
SPBC2F12.15c	-0.95	2.78E-04
SPAC1786.01c	-0.95	1.68E-04
SPAP32A8.02	-0.95	1.26E-04

SPBC1271.05c	-0.95	2.85E-03
SPBC16E9.16c	-0.94	4.50E-04
SPBC365.20c	-0.93	9.54E-04
SPAC29A4.12c	-0.93	1.84E-02
SPBP19A11.01	-0.93	8.52E-07
SPCC63.03	-0.93	3.31E-02
SPAC24H6.05	-0.93	7.03E-05
SPAC869.09	-0.92	3.59E-02
SPCC1259.15c	-0.92	5.18E-07
SPAC27D7.04	-0.92	3.36E-06
SPAC12B10.10	-0.91	3.95E-02
SPBC354.12	-0.91	1.66E-06
SPCC1223.12c	-0.90	3.36E-03
SPBC146.04	-0.89	4.44E-03
SPBC21C3.16c	-0.89	1.18E-05
SPBC577.08c	-0.89	1.39E-05
SPAC688.03c	-0.88	8.76E-04
SPCC1919.04	-0.88	9.77E-04
SPAC3A12.14	-0.88	1.79E-07
SPBC211.01	-0.88	1.18E-04
SPBC1198.13c	-0.88	5.20E-06
SPAC12B10.06c	-0.87	1.57E-02
SPBC1773.02c	-0.87	3.76E-03
SPAC6G10.12c	-0.87	2.71E-02
SPCC188.02	-0.87	1.73E-08
SPAC15A10.05c	-0.87	1.67E-02
SPAC1B3.10c	-0.87	3.86E-02
SPAC23A1.02c	-0.86	8.86E-03
SPAC4G9.13c	-0.86	3.94E-03
SPACUNK4.16c	-0.85	6.56E-03
SPAC1B3.20	-0.85	1.93E-02

SPBC3H7.13	-0.85	1.99E-04
SPCC1672.08c	-0.85	3.90E-07
SPBC119.06	-0.85	2.16E-04
SPAC23A1.05	-0.84	1.14E-03
SPCC191.03c	-0.84	2.87E-03
SPAC144.17c	-0.84	2.86E-05
SPAC57A10.08c	-0.84	7.06E-05
SPAC6F12.02	-0.83	1.75E-05
SPBC21H7.06c	-0.83	1.70E-02
SPCC11E10.02c	-0.83	6.38E-07
SPBC1709.18	-0.83	2.02E-07
SPAC4A8.02c	-0.82	3.94E-04
SPAC13D6.05	-0.82	9.99E-07
SPCC736.10c	-0.82	4.83E-04
SPAC31A2.08	-0.82	1.10E-02
SPAC25G10.01	-0.82	5.24E-03
SPAC17G6.09	-0.82	2.27E-05
SPCC1183.09c	-0.82	3.40E-03
SPBC3B9.01	-0.82	2.16E-02
SPAP8A3.02c	-0.81	3.77E-02
SPAC25B8.18	-0.81	7.38E-04
SPAC11D3.15	-0.81	2.09E-02
SPAC513.07	-0.81	3.57E-02
SPBC1604.20c	-0.80	4.51E-02
SPCC622.02	-0.80	5.30E-04
SPBC19F8.05	-0.80	3.61E-03
SPAPB2B4.03	-0.80	1.46E-02
SPBC660.14	-0.80	2.04E-02
SPBC405.04c	-0.80	2.75E-04

	Δgcn5 Δelp3			*Δgcn5 Δmst2*			*Δmst2 Δelp3*	
Gene	Log2 Change	pvalue	Gene	Log2 Change	pvalue	Gene	Log2 Change	pvalue
SPAC29A4.20	-5.81	3.53E-15	SPBC11B10.02c	-7.51	3.17E-04	SPAC29A4.20	-5.45	1.53E-15
SPAC1952.05	-5.62	6.60E-04	SPAC1952.05	-5.32	3.85E-04	SPBPB2B2.09c	-5.12	1.07E-13
SPAC57A10.06	-3.46	5.93E-04	SPBPB10D8.02c	-5.00	2.88E-08	SPBPB2B2.06c	-4.69	1.86E-08
SPCC794.04c	-3.34	1.09E-04	SPBPB10D8.01	-4.81	3.53E-08	SPAC17G8.13c	-4.37	8.06E-04
SPBPB2B2.08	-2.75	1.11E-02	SPBPB2B2.06c	-4.77	1.43E-08	SPBPB2B2.05	-3.73	4.40E-04
SPBC215.11c	-2.67	5.73E-05	SPAC1039.02	-4.73	8.49E-07	SPBPB10D8.02c	-3.70	1.98E-06
SPAC1039.09	-2.41	1.82E-02	SPAC186.05c	-4.59	2.32E-08	SPBPB2B2.10c	-3.62	2.63E-06
SPAC869.05c	-2.32	1.45E-03	SPAC17G8.13c	-4.41	7.41E-04	SPBPB2B2.08	-3.40	1.02E-03
SPCC965.07c	-2.09	3.99E-02	SPBPB2B2.05	-4.06	1.93E-04	SPBPB2B2.13	-3.36	2.77E-04
SPAC18G6.12c	-2.08	1.37E-05	SPAC977.14c	-4.03	3.87E-04	SPCC794.04c	-3.24	4.43E-05
SPCC569.03	-2.06	8.21E-06	SPAC186.03	-3.63	7.22E-10	SPBPB2B2.01	-3.02	3.01E-06
SPAC23H3.15c	-2.05	4.57E-03	SPAC186.06	-3.41	2.97E-06	SPBC660.05	-2.94	1.64E-03
SPBC106.02c	-2.04	4.26E-04	SPBPB2B2.01	-3.30	9.24E-07	SPAC22F8.05	-2.58	8.09E-05
SPCC663.09c	-2.00	1.04E-04	SPAC57A10.06	-3.11	5.55E-04	SPAC57A10.06	-2.58	2.76E-03
SPAC22H10.13	-1.99	2.51E-03	SPBC359.04c	-2.98	1.81E-09	SPBC4F6.09	-2.44	5.05E-05
SPAC1F7.07c	-1.98	2.69E-03	SPBPB21E7.10	-2.89	1.02E-05	SPAC869.05c	-2.41	3.81E-04
SPAC29B12.10c	-1.95	6.76E-04	SPAC8E11.10	-2.87	5.39E-09	SPCC1235.14	-2.40	2.87E-08
SPBC27.05	-1.93	1.65E-05	SPBC947.04	-2.80	1.92E-06	SPAC23H3.15c	-2.32	6.68E-04
SPAC1F12.03c	-1.92	3.52E-04	SPBPB2B2.08	-2.59	8.09E-03	SPCC569.03	-2.22	6.85E-07
SPBC16A3.16	-1.90	1.98E-03	SPAC977.05c	-2.38	7.28E-04	SPCC191.11	-2.13	1.48E-02
SPBC4F6.09	-1.89	1.79E-03	SPBC26H8.11c	-2.35	4.53E-08	SPBPB10D8.01	-2.10	8.44E-04
SPBPB2B2.01	-1.88	1.61E-03	SPAC3C7.14c	-2.35	9.26E-04	SPBC16A3.16	-1.97	5.65E-04
SPBC23G7.13c	-1.83	1.88E-02	SPCC663.09c	-2.34	4.39E-06	SPBC1683.08	-1.89	8.80E-03
SPBPB2B2.06c	-1.82	3.75E-03	SPBC1683.02	-2.31	2.23E-06	SPCC622.05	-1.85	1.07E-04
SPCC1902.02	-1.80	8.76E-05	SPAC977.15	-2.26	3.04E-02	SPAP8A3.04c	-1.79	1.88E-03
SPAC17A5.09c	-1.79	2.52E-08	SPAC1F8.06	-2.17	1.44E-06	SPBC29B5.02c	-1.74	5.36E-07
SPBC29B5.02c	-1.76	2.08E-06	SPCC965.14c	-2.14	9.02E-05	SPBC21C3.19	-1.67	4.47E-02
SPBC8D2.09c	-1.58	2.92E-03	SPBC3H7.07c	-2.12	3.86E-07	SPCC1902.02	-1.66	6.45E-05
SPBC2A9.02	-1.56	1.55E-02	SPAC23C4.06c	-2.10	3.77E-05	SPBPB2B2.02	-1.64	2.22E-03

SPBC651.04	-1.55	4.97E-05	SPCC1223.13	-2.09	1.45E-04	SPAC27D7.11c	-1.64	4.70E-04
SPAPYUG7.04c	-1.55	3.96E-04	SPCC569.05c	-2.07	7.09E-04	SPMIT.05	-1.62	5.03E-03
SPBPB10D8.02c	-1.54	1.95E-02	SPAC24B11.14	-2.07	2.73E-03	SPAC977.05c	-1.58	1.46E-02
SPAC26F1.04c	-1.53	6.29E-06	SPAC869.05c	-2.05	1.65E-03	SPAC18G6.12c	-1.54	1.16E-04
SPBC660.14	-1.51	4.12E-04	SPBPB2B2.18	-1.95	1.44E-03	SPAC1B3.20	-1.52	2.25E-04
SPAC29B12.11c	-1.51	2.30E-05	SPAC8C9.05	-1.94	5.72E-07	SPCC320.06	-1.52	2.31E-06
SPBC26H8.11c	-1.51	6.60E-05	SPBC359.03c	-1.91	1.26E-11	SPAC22H10.13	-1.48	9.21E-03
SPCC338.18	-1.48	4.62E-03	SPAC29B12.10c	-1.87	3.53E-04	SPAC1782.12c	-1.46	6.28E-04
SPAC589.09	-1.47	2.20E-04	SPBC1271.08c	-1.83	3.88E-03	SPAC18G6.01c	-1.39	4.68E-03
SPCC965.06	-1.46	3.05E-04	SPBC106.02c	-1.80	4.98E-04	SPBC21H7.06c	-1.39	3.69E-04
SPBC1271.08c	-1.44	3.17E-02	SPAC869.02c	-1.76	1.16E-04	SPAC1834.03c	-1.38	4.61E-06
SPAC3F10.15c	-1.43	3.20E-03	SPAC17C9.16c	-1.76	5.57E-08	SPAC2F3.05c	-1.37	1.51E-05
SPBC1271.05c	-1.42	2.03E-04	SPBC2A9.02	-1.74	3.71E-03	SPBC106.02c	-1.36	4.92E-03
SPBC365.01	-1.41	3.33E-06	SPBPB21E7.07	-1.72	2.78E-04	SPAC15A10.05c	-1.34	7.05E-04
SPAC6G10.12c	-1.38	3.12E-03	SPAC2E1P3.05c	-1.70	1.36E-08	SPACUNK4.17	-1.34	5.69E-03
SPCC320.06	-1.36	3.41E-05	SPCC1682.09c	-1.69	5.46E-06	SPAC24C9.16c	-1.33	6.37E-04
SPAC1F7.08	-1.36	4.91E-02	SPAC5H10.03	-1.67	6.49E-07	SPAPYUG7.04c	-1.33	5.75E-04
SPBC21C3.04c	-1.35	7.30E-05	SPBP4H10.20	-1.57	6.02E-10	SPBP4H10.10	-1.32	2.56E-02
SPAC13C5.04	-1.34	1.36E-03	SPAC23H3.13c	-1.55	7.95E-07	SPBC215.11c	-1.30	1.06E-02
SPBC106.17c	-1.33	6.19E-04	SPBC12D12.07c	-1.54	7.64E-05	SPCC757.07c	-1.26	1.13E-02
SPCC285.04	-1.32	2.97E-04	SPBC337.09	-1.52	8.40E-09	SPAC22A12.17c	-1.23	7.99E-05
SPBP26C9.02c	-1.29	8.88E-06	SPAC688.02c	-1.51	3.12E-05	SPAC167.06c	-1.21	4.54E-03
SPBC19F8.05	-1.29	1.43E-04	SPBC1861.01c	-1.51	4.17E-03	SPAC23C11.10	-1.20	1.62E-03
SPCC1620.01c	-1.27	1.34E-05	SPBC16A3.16	-1.50	5.00E-03	SPCC191.03c	-1.20	1.09E-04
SPAC9E9.11	-1.27	8.38E-03	SPBC365.11	-1.45	1.39E-03	SPCC1739.06c	-1.19	7.81E-05
SPBC3H7.07c	-1.27	5.96E-04	SPBC106.17c	-1.45	8.85E-05	SPAC26F1.04c	-1.19	3.30E-05
SPAC19G12.09	-1.27	2.62E-04	SPAC513.07	-1.43	7.98E-04	SPAC343.12	-1.18	1.67E-02
SPAC31G5.21	-1.26	4.38E-03	SPBC215.11c	-1.42	6.12E-03	SPAC869.09	-1.17	9.62E-03
SPAC18B11.04	-1.25	1.10E-04	SPAC11D3.17	-1.41	4.98E-03	SPAC25B8.01	-1.16	1.56E-03
SPAC1786.01c	-1.25	2.77E-05	SPBC25B2.08	-1.40	4.91E-05	SPAC29B12.10c	-1.15	1.44E-02
SPCC18B5.09c	-1.24	9.61E-06	SPBP8B7.31	-1.39	2.00E-04	SPBC20F10.06	-1.14	2.02E-03

SPAC1834.03c	-1.22	7.83E-05	SPAPB24D3.08c	-1.38	1.34E-02	SPAC31G5.21	-1.11	4.91E-03
SPAC2F3.05c	-1.21	2.09E-04	SPBC31F10.17c	-1.37	1.72E-02	SPBC887.17	-1.11	5.51E-05
SPAC1687.14c	-1.21	1.46E-03	SPBC17D1.06	-1.36	1.01E-03	SPBC8D2.09c	-1.10	1.47E-02
SPBC56F2.14	-1.21	2.27E-02	SPAC977.09c	-1.35	1.27E-04	SPAC589.09	-1.10	1.11E-03
SPAC17A2.10c	-1.19	1.19E-02	SPAC589.09	-1.35	1.68E-04	SPBC725.03	-1.10	1.32E-02
SPCC1682.08c	-1.19	3.92E-03	SPBC16D10.01c	-1.34	5.78E-04	SPBC16E9.16c	-1.10	9.95E-05
SPAC23A1.09	-1.18	1.43E-04	SPAC1399.02	-1.34	2.92E-07	SPAC17A5.09c	-1.09	5.42E-06
SPAC17A5.10	-1.17	7.85E-04	SPCC622.01c	-1.33	7.03E-06	SPAC869.10c	-1.09	9.65E-03
SPAC11G7.01	-1.17	1.15E-02	SPBC365.04c	-1.32	1.16E-03	SPAC23H3.12c	-1.09	3.46E-04
SPBC2F12.15c	-1.17	1.04E-04	SPBPB2B2.02	-1.31	1.06E-02	SPCC63.03	-1.09	1.47E-02
SPAC6C3.04	-1.17	1.60E-08	SPCC285.05	-1.30	5.61E-03	SPCC663.09c	-1.08	7.79E-03
SPAP8A3.02c	-1.17	1.00E-02	SPAC9E9.11	-1.29	3.45E-03	SPAC1F12.03c	-1.07	1.38E-02
SPAC458.06	-1.16	1.50E-05	SPBC2G2.05	-1.27	2.31E-07	SPBC4B4.08	-1.06	9.99E-05
SPBC20F10.06	-1.15	4.60E-03	SPBC1718.03	-1.27	7.62E-04	SPCC1494.09c	-1.06	3.51E-05
SPAPB1E7.04c	-1.14	4.38E-03	SPBC1198.02	-1.25	8.82E-04	SPAC637.03	-1.06	2.16E-02
SPBC3E7.02c	-1.14	4.65E-02	SPAC19G12.05	-1.25	2.52E-05	SPAC27D7.04	-1.05	5.14E-07
SPBC887.17	-1.14	1.30E-04	SPAC750.07c	-1.25	5.33E-03	SPAC29A4.17c	-1.05	4.32E-03
SPCC191.03c	-1.14	5.33E-04	SPBCPT2R1.02	-1.25	1.38E-04	SPAC26F1.14c	-1.05	7.37E-03
SPBC3B8.10c	-1.13	2.81E-03	SPBPB10D8.07c	-1.24	2.06E-04	SPAC23C4.11	-1.04	2.85E-03
SPBC83.04	-1.13	3.97E-03	SPCC4B3.18	-1.24	5.28E-05	SPAC23A1.09	-1.04	1.69E-04
SPCC1223.09	-1.13	5.71E-04	SPBC216.04c	-1.24	1.46E-03	SPBC20F10.03	-1.04	2.94E-03
SPAC15A10.05c	-1.11	7.50E-03	SPBC4B4.11	-1.23	5.28E-04	SPAC1A6.08c	-1.03	1.34E-02
SPAC1093.01	-1.10	2.86E-05	SPCC1259.02c	-1.23	1.19E-08	SPBP8B7.08c	-1.03	5.97E-04
SPCC24B10.20	-1.10	1.08E-02	SPBC2G2.15c	-1.22	7.76E-08	SPBC354.12	-1.02	3.41E-04
SPBP23A10.12	-1.10	2.05E-04	SPAC16A10.05c	-1.20	4.87E-02	SPAC19A8.09	-1.02	1.72E-03
SPCC1682.09c	-1.09	1.71E-03	SPCC1393.10	-1.20	2.55E-04	SPAC3H8.07c	-1.02	1.90E-03
SPCC622.02	-1.09	7.34E-05	SPAC1250.04c	-1.19	1.55E-03	SPAC2F3.17c	-1.01	1.32E-02
SPBC6B1.02	-1.09	1.13E-05	SPBP8B7.28c	-1.18	3.12E-03	SPAC29B12.11c	-1.00	5.31E-04
SPBC1773.02c	-1.09	1.61E-03	SPBC2G2.01c	-1.17	3.95E-05	SPBC25B2.08	-1.00	1.35E-03
SPBP22H7.03	-1.08	4.10E-04	SPACUNK4.09	-1.17	6.96E-05	SPAP32A8.02	-0.99	7.50E-05
SPCC63.03	-1.07	2.83E-02	SPBP16F5.05c	-1.17	1.47E-03	SPAC23A1.02c	-0.99	3.30E-03

SPAC1805.16c	-1.07	7.00E-06	SPBC1683.05	-1.16	7.95E-05	SPAC139.05	-0.99	2.34E-02
SPAC5H10.05c	-1.07	2.17E-03	SPBC83.15	-1.16	2.27E-04	SPBC56F2.14	-0.99	3.49E-02
SPBC19F8.07	-1.06	8.84E-04	SPAC31G5.08	-1.15	1.42E-07	SPCC338.12	-0.99	3.09E-03
SPBC26H8.13c	-1.06	2.65E-03	SPAC12G12.02	-1.15	6.67E-04	SPBC1677.02	-0.98	2.30E-03
SPAC1B3.10c	-1.06	2.54E-02	SPBC1703.08c	-1.14	5.27E-03	SPBC337.16	-0.98	6.34E-05
SPBC577.08c	-1.05	6.60E-06	SPAC1F12.03c	-1.14	8.97E-03	SPBC25B2.10	-0.98	1.07E-04
SPBC713.08	-1.05	7.90E-04	SPAC10F6.10	-1.14	4.44E-03	SPBC887.01	-0.97	7.00E-03
SPBC1685.03	-1.05	6.02E-05	SPAC1039.04	-1.13	1.30E-04	SPAC3G6.02	-0.97	6.00E-04
SPBC211.01	-1.05	5.62E-05	SPAC4H3.01	-1.13	2.06E-04	SPAC7D4.05	-0.97	1.34E-03
SPAC24H6.05	-1.04	6.62E-05	SPCC18B5.01c	-1.12	1.26E-03	SPBC365.12c	-0.95	1.37E-02
SPBC15D4.08c	-1.04	4.43E-02	SPBC1861.02	-1.12	2.57E-02	SPAC22F3.05c	-0.95	2.57E-02
SPAC1B3.20	-1.03	1.20E-02	SPBC1271.07c	-1.11	1.35E-05	SPBC21C3.16c	-0.94	6.03E-06
SPAC869.09	-1.03	3.49E-02	SPCC4B3.10c	-1.11	1.33E-03	SPBC11G11.04	-0.94	1.07E-03
SPAC5D6.09c	-1.03	7.34E-04	SPAC56F8.10	-1.11	4.38E-08	SPBC685.05	-0.94	5.44E-03
SPBC4F6.08c	-1.03	1.58E-02	SPAC977.10	-1.10	1.15E-07	SPBC19F8.05	-0.94	1.04E-03
SPBC27B12.11c	-1.02	1.97E-04	SPBC1734.14c	-1.10	1.07E-03	SPBC1711.09c	-0.94	1.93E-05
SPAC12B10.10	-1.02	4.04E-02	SPAC926.05c	-1.10	2.28E-03	SPCC191.01	-0.93	6.80E-03
SPBC27.04	-1.02	2.52E-02	SPBC4F6.09	-1.10	2.85E-02	SPBC119.06	-0.93	8.38E-05
SPAC27D7.04	-1.01	3.94E-06	SPAC20H4.08	-1.10	5.12E-03	SPCC1620.01c	-0.92	1.46E-04
SPCC23B6.05c	-1.01	7.31E-03	SPAC1D4.01	-1.10	8.07E-03	SPCC23B6.05c	-0.92	6.54E-03
SPBC21C3.16c	-1.01	1.07E-05	SPCC338.11c	-1.09	2.18E-03	SPBC365.20c	-0.92	1.11E-03
SPBC428.13c	-1.01	5.78E-03	SPAC11D3.02c	-1.09	2.08E-04	SPBC3B8.10c	-0.91	5.90E-03
SPCC622.01c	-1.00	5.11E-04	SPBC2A9.12	-1.09	1.92E-02	SPCC338.18	-0.91	3.85E-02
SPBC12D12.07c	-1.00	8.08E-03	SPAC11E3.03	-1.09	1.59E-02	SPAC3H5.05c	-0.91	1.61E-05
SPBC119.06	-1.00	1.24E-04	SPBC11G11.05	-1.09	3.88E-03	SPCC188.02	-0.91	8.51E-09
SPAC27D7.07c	-1.00	1.34E-03	SPBC21D10.07	-1.08	2.92E-06	SPBP8B7.13	-0.91	1.03E-05
SPAC869.10c	-0.99	2.97E-02	SPBC23E6.06c	-1.08	3.80E-06	SPAC458.06	-0.91	6.88E-05
SPAC328.10c	-0.99	2.09E-04	SPCC4G3.17	-1.08	2.94E-05	SPAC869.06c	-0.90	1.90E-02
SPBC17D11.03c	-0.99	2.20E-05	SPAC227.16c	-1.08	6.91E-05	SPBC13E7.11	-0.89	1.21E-05
SPBC428.18	-0.98	3.35E-03	SPBC215.06c	-1.07	3.81E-02	SPAC3H8.03	-0.89	6.49E-04
SPBC11G11.06c	-0.98	1.01E-03	SPAC20G4.01	-1.07	2.70E-08	SPBC6B1.02	-0.88	3.57E-05

SPBC216.04c	-0.98	1.58E-02	SPCC663.13c	-1.06	1.12E-02	SPBC800.12c	-0.88	1.52E-04
SPBC106.15	-0.98	2.02E-05	SPBC1711.05	-1.05	4.59E-04	SPAC15E1.08	-0.88	1.30E-03
SPCC188.02	-0.98	1.58E-08	SPBC651.04	-1.05	8.32E-04	SPAC12B10.10	-0.88	4.65E-02
SPBC30D10.14	-0.97	5.51E-04	SPAC1399.04c	-1.04	3.65E-02	SPCC622.02	-0.88	2.19E-04
SPAC25B8.01	-0.96	1.27E-02	SPBC3B8.06	-1.04	7.05E-06	SPBC20F10.10	-0.88	1.77E-02
SPCC18.01c	-0.96	1.69E-03	SPCC126.03	-1.03	1.90E-03	SPAC9E9.14	-0.87	1.26E-03
SPBP4H10.11c	-0.96	2.11E-03	SPAC589.03c	-1.03	1.43E-03	SPAC869.07c	-0.87	1.20E-04
SPAC110.01	-0.95	1.04E-05	SPBC3H7.14	-1.03	8.78E-04	SPBC3H7.02	-0.87	4.00E-05
SPBC20F10.10	-0.94	2.23E-02	SPBC359.05	-1.03	6.28E-03	SPAC4F10.20	-0.87	1.47E-03
SPAC8C9.16c	-0.94	4.09E-03	SPCC16A11.07	-1.02	2.53E-03	SPBC11G11.06c	-0.87	1.14E-03
SPAC23A1.02c	-0.94	1.05E-02	SPAC4G9.10	-1.02	1.04E-05	SPAC16A10.06c	-0.87	7.44E-03
SPAC869.06c	-0.94	2.73E-02	SPAC11D3.03c	-1.01	3.65E-03	SPBC31F10.15c	-0.86	3.93E-03
SPBC725.12	-0.93	3.69E-02	SPCC24B10.20	-1.01	9.41E-03	SPAC222.03c	-0.86	1.48E-02
SPBC119.05c	-0.93	3.95E-03	SPAC227.05	-1.01	2.81E-02	SPBC23E6.03c	-0.86	8.65E-03
SPBC14C8.10	-0.93	5.93E-04	SPAC27D7.07c	-1.00	4.73E-04	SPBC4F6.08c	-0.86	2.30E-02
SPBC651.06	-0.93	1.42E-02	SPBC3B9.07c	-0.99	9.59E-04	SPBC4B4.05	-0.86	2.59E-02
SPBC17G9.12c	-0.92	3.17E-02	SPAC328.09	-0.99	2.26E-03	SPCC622.01c	-0.86	7.95E-04
SPAC17G6.02c	-0.92	2.92E-04	SPBC25H2.04c	-0.99	9.46E-05	SPAC15A10.07	-0.85	3.57E-03
SPAC9E9.14	-0.92	1.99E-03	SPAC140.03	-0.99	1.23E-03	SPBC21C3.04c	-0.85	1.99E-03
SPAC17G6.09	-0.92	2.04E-05	SPCC330.03c	-0.99	1.07E-03	SPAC1786.01c	-0.85	5.15E-04
SPAC823.06	-0.92	1.82E-02	SPAC29A4.14c	-0.98	1.32E-04	SPMIT.11	-0.85	4.84E-03
SPAC13D6.05	-0.92	9.52E-07	SPAC23C11.13c	-0.98	3.36E-05	SPBC359.04c	-0.84	5.58E-03
SPAC3A12.14	-0.92	4.37E-07	SPAC20G4.04c	-0.98	5.35E-03	SPAC8C9.16c	-0.84	4.07E-03
SPCC191.01	-0.92	1.49E-02	SPBC23G7.07c	-0.97	3.19E-02	SPAC1834.05	-0.84	3.29E-06
SPAC20G4.03c	-0.92	2.71E-02	SPBC1539.10	-0.97	1.50E-02	SPBC211.01	-0.83	1.99E-04
SPAC227.08c	-0.91	1.61E-03	SPAC19B12.12c	-0.97	1.74E-04	SPAC25G10.02	-0.83	8.43E-03
SPBC337.16	-0.91	4.23E-04	SPAC5H10.06c	-0.97	6.81E-04	SPCC18B5.09c	-0.83	2.39E-04
SPBC16C6.03c	-0.91	3.96E-05	SPBC32H8.08c	-0.97	1.96E-05	SPAC11D3.10	-0.83	9.40E-06
SPBC1709.18	-0.91	2.52E-07	SPBP35G2.13c	-0.96	2.00E-03	SPCC330.10	-0.82	9.31E-04
SPBC32H8.02c	-0.91	1.68E-02	SPAC1486.08	-0.96	3.15E-02	SPBC2D10.09	-0.82	1.97E-05
SPBC685.05	-0.90	1.41E-02	SPAPB1E7.10	-0.96	1.08E-02	SPCC1672.04c	-0.82	1.05E-02

SPAC589.03c	-0.90	8.87E-03	SPAC14C4.12c	-0.96	6.10E-03	SPBC800.14c	-0.82	3.93E-03
SPBC25B2.11	-0.90	8.61E-04	SPCC1919.13c	-0.95	8.78E-04	SPAC589.03c	-0.82	8.19E-03
SPBC887.01	-0.90	2.19E-02	SPAC6B12.09	-0.95	4.34E-03	SPAC12B10.06c	-0.81	2.29E-02
SPAC144.08	-0.90	1.55E-04	SPAC1952.06c	-0.95	9.74E-06	SPAC23C4.09c	-0.81	1.84E-02
SPBC3H7.13	-0.89	3.56E-04	SPCC1840.07c	-0.95	1.58E-05	SPAC1610.01	-0.81	1.17E-03
SPBC1D7.03	-0.89	9.54E-03	SPBC19C7.07c	-0.94	9.43E-04	SPAC25B8.18	-0.81	8.09E-04
SPAC23A1.05	-0.89	1.80E-03	SPAC25B8.02	-0.94	6.63E-06	SPAC15A10.12c	-0.80	2.04E-02
SPAC17A2.05	-0.89	1.83E-02	SPCC622.02	-0.94	1.10E-04	SPAC11D3.15	-0.80	2.23E-02
SPAC10F6.07c	-0.89	1.62E-03	SPBC16D10.02	-0.94	3.25E-04	SPBC16D10.06	-0.80	2.43E-04
SPAC24H6.08	-0.89	1.44E-04	SPCC1739.05	-0.94	3.01E-03	SPAC11H11.02c	-0.80	4.63E-03
SPBC1703.09	-0.89	3.06E-03	SPCC1281.07c	-0.93	1.01E-02	SPBPB2B2.11	-0.80	4.15E-03
SPCC16A11.07	-0.88	1.43E-02	SPAC17A2.14	-0.93	2.07E-07	SPBC2D10.03c	-0.80	3.63E-04
SPAC11D3.16c	-0.88	2.27E-03	SPAC16C9.02c	-0.93	3.32E-05	SPBC26H8.11c	-0.80	6.76E-03
SPBC21D10.07	-0.88	1.26E-04	SPAC3H1.07	-0.92	9.62E-04	SPAC19D5.02c	-0.80	1.24E-04
SPBC646.08c	-0.88	1.26E-04	SPBC725.16	-0.92	1.42E-03			
SPAC3H1.11	-0.88	4.33E-03	SPAC23H3.14	-0.92	7.28E-07			
SPBC365.20c	-0.87	4.02E-03	SPBC2G2.08	-0.92	5.20E-03			
SPCC1442.16c	-0.87	8.93E-05	SPBC19G7.04	-0.92	1.06E-02			
SPAC513.07	-0.87	4.12E-02	SPCC1682.08c	-0.91	1.08E-02			
SPBC16E9.01c	-0.87	1.03E-03	SPAC3G6.05	-0.91	1.94E-05			
SPBC14C8.07c	-0.87	1.46E-02	SPBP23A10.12	-0.91	4.61E-04			
SPCC1235.14	-0.87	7.60E-03	SPAC1834.07	-0.90	8.70E-05			
SPCC1919.04	-0.86	2.74E-03	SPAC56F8.09	-0.90	5.09E-03			
SPBC11G11.04	-0.86	5.20E-03	SPBC30D10.15	-0.90	2.02E-03			
SPAC664.13	-0.86	4.73E-04	SPBC12D12.02c	-0.90	2.97E-03			
SPAC23C4.11	-0.86	2.06E-02	SPBC19C2.03	-0.89	3.24E-03			
SPCC1672.08c	-0.85	1.63E-06	SPAC5H10.12c	-0.89	6.27E-04			
SPCC736.10c	-0.85	9.25E-04	SPAC24H6.11c	-0.89	3.09E-04			
SPAC12B10.06c	-0.85	3.17E-02	SPAC8C9.10c	-0.89	9.33E-03			
SPCC1259.15c	-0.85	6.07E-06	SPAC1952.03	-0.88	9.83E-03			
SPAC6F12.06	-0.85	4.20E-05	SPBP26C9.02c	-0.88	1.94E-04			

SPAC22F8.02c	-0.85	4.59E-04	SPBC577.04	-0.88	1.93E-03
SPCC1795.04c	-0.84	1.69E-05	SPAC11G7.01	-0.88	3.01E-02
SPBP19A11.01	-0.84	1.25E-05	SPBC21.03c	-0.87	2.81E-03
SPBC19F5.03	-0.84	2.18E-04	SPCC737.06c	-0.87	1.05E-04
SPBP8B7.08c	-0.84	7.08E-03	SPBC25B2.05	-0.87	2.10E-02
SPCC622.12c	-0.84	2.46E-06	SPAC13G7.09c	-0.87	1.54E-02
SPBC428.04	-0.84	1.45E-03	SPAC5H10.05c	-0.87	4.45E-03
SPAC227.16c	-0.84	2.21E-03	SPAC56E4.07	-0.86	5.28E-05
SPAC31G5.18c	-0.84	3.07E-05	SPAC1B3.16c	-0.86	1.43E-03
SPCC24B10.05	-0.83	7.87E-03	SPCC18.12c	-0.86	2.27E-02
SPBC24C6.06	-0.83	7.28E-03	SPBC2D10.15c	-0.86	4.18E-04
SPAC6G9.11	-0.83	7.63E-04	SPAC23G3.05c	-0.86	3.53E-03
SPAC3H1.14	-0.83	1.83E-02	SPBC839.03c	-0.86	5.61E-05
SPAC24C9.16c	-0.83	3.48E-02	SPAC17G8.05	-0.86	4.74E-04
SPAC16E8.13	-0.82	1.35E-05	SPAC607.04	-0.86	3.40E-03
SPAPB1A11.02	-0.82	1.30E-02	SPAC821.09	-0.85	2.22E-02
SPBC146.04	-0.82	1.61E-02	SPBC887.01	-0.85	1.62E-02
SPAPB1A10.10c	-0.82	5.45E-04	SPBC342.06c	-0.85	7.29E-03
SPBC543.08	-0.82	1.90E-03	SPAC2C4.08	-0.85	3.40E-05
SPCC126.06	-0.81	9.48E-07	SPAC4F10.06	-0.84	4.16E-02
SPCC290.04	-0.81	3.26E-02	SPAC1952.02	-0.84	5.02E-03
SPBC691.04	-0.81	1.39E-03	SPAC16E8.04c	-0.84	1.94E-05
SPAC16A10.04	-0.81	6.17E-03	SPBC36B7.08c	-0.84	5.79E-03
SPBC211.07c	-0.81	1.38E-03	SPAP8A3.11c	-0.84	1.12E-03
SPCC24B10.13	-0.81	7.35E-04	SPCC757.10	-0.84	1.41E-05
SPBPB10D8.07c	-0.81	1.48E-02	SPAC5H10.10	-0.84	4.97E-04
SPAC3G6.10c	-0.81	4.32E-02	SPAC11D3.05	-0.83	3.88E-02
SPBC19G7.04	-0.81	3.74E-02	SPAP7G5.04c	-0.83	1.17E-05
SPAC23C11.08	-0.81	1.21E-02	SPCC1827.04	-0.83	1.19E-02
SPCC962.03c	-0.81	1.78E-02	SPAC26F1.12c	-0.83	1.34E-02
SPAC26A3.10	-0.80	1.03E-03	SPBC30B4.08	-0.83	2.45E-06

SPAC23H3.12c	-0.80	9.50E-03	SPAC29B12.06c	-0.82	5.65E-04
SPBC3B9.01	-0.80	4.07E-02	SPAC4G8.07c	-0.82	1.82E-02
			SPCC4G3.16	-0.82	4.43E-02
			SPBC13A2.03	-0.82	3.58E-05
			SPAC17A5.13	-0.82	5.04E-04
			SPAC22E12.13c	-0.81	9.02E-03
			SPCC330.07c	-0.81	2.68E-04
			SPAC2E1P5.03	-0.81	4.99E-04
			SPAP27G11.04c	-0.81	1.56E-03
			SPBC1703.05	-0.81	2.06E-02
			SPAC890.04c	-0.81	4.09E-03
			SPBC839.07	-0.80	2.49E-03
			SPAC57A10.10c	-0.80	6.72E-03
			SPAC3G9.16c	-0.80	5.04E-04
			SPBP22H7.06	-0.80	2.83E-05
			SPBC13G1.03c	-0.80	2.31E-02

Δ*gcn5* Δ*elp3* Δ*mst2*

Gene	Log2 Change	pvalue	Gene	Log2 Change	pvalue	Gene	Log2 Change	pvalue
SPAC1952.05	-5.66	4.19E-03	SPCC285.05	-1.25	4.75E-02	SPBP8B7.31	-0.95	3.75E-02
SPAC29A4.20	-5.64	3.29E-13	SPAC3G6.10c	-1.24	1.65E-02	SPBC25B2.11	-0.95	3.69E-03
SPBPB10D8.01	-4.74	5.16E-06	SPCC965.13	-1.24	3.47E-05	SPAC17G8.05	-0.95	3.74E-03
SPBPB10D8.02c	-4.74	7.09E-06	SPAC12B10.10	-1.23	4.84E-02	SPAC17A5.09c	-0.95	1.02E-03
SPAC17G8.13c	-4.41	1.02E-02	SPAC15E1.08	-1.22	1.55E-03	SPAC4H3.13	-0.95	2.73E-02
SPAC57A10.06	-3.73	2.28E-03	SPBC887.01	-1.21	1.52E-02	SPAC110.01	-0.95	1.64E-04
SPBPB2B2.06c	-3.65	5.26E-05	SPAC227.16c	-1.20	7.38E-04	SPBC1703.09	-0.95	9.76E-03
SPBC26H8.11c	-3.64	1.20E-08	SPAC26H5.02c	-1.20	4.71E-06	SPBC23G7.14	-0.95	1.04E-02
SPAC1039.02	-3.40	1.56E-03	SPAC869.10c	-1.19	3.76E-02	SPAC22G7.01c	-0.95	1.44E-05
SPAC2E1P3.05c	-3.17	1.71E-10	SPBC16D10.06	-1.19	1.48E-04	SPBC1105.12	-0.94	8.94E-03

SPBPB2B2.01	-2.88	2.83E-04	SPBC20F10.06	-1.18	1.68E-02	SPAC328.10c	-0.94	2.71E-03
SPAC186.06	-2.78	1.16E-03	SPAC5H10.03	-1.18	1.56E-03	SPAC23H4.13c	-0.93	1.28E-02
SPAC977.05c	-2.60	5.56E-03	SPAC11H11.02c	-1.15	4.11E-03	SPAC31G5.14	-0.92	2.22E-03
SPAC186.05c	-2.53	1.73E-03	SPAC5H10.05c	-1.15	6.87E-03	SPAC56E4.04c	-0.92	6.27E-05
SPAC29B12.10c	-2.53	5.33E-04	SPBC1861.05	-1.15	1.87E-02	SPAC6C3.04	-0.92	1.15E-05
SPBC16A3.16	-2.39	2.04E-03	SPBPB10D8.07c	-1.14	7.43E-03	SPAC23H4.09	-0.92	4.79E-05
SPAC186.03	-2.36	3.93E-05	SPAC29A4.14c	-1.14	9.08E-04	SPBC12D12.05c	-0.91	6.63E-04
SPAC869.05c	-2.21	1.13E-02	SPAC2F3.09	-1.13	8.44E-05	SPAC20G4.01	-0.91	2.66E-05
SPAC1F12.03c	-2.17	9.71E-04	SPBC26H8.09c	-1.13	1.50E-03	SPBC337.16	-0.91	3.32E-03
SPAC18G6.12c	-2.15	1.37E-04	SPBC1271.14	-1.13	8.08E-03	SPAC56E4.07	-0.90	1.08E-03
SPBC947.04	-2.11	1.73E-03	SPBC3B8.07c	-1.13	1.22E-04	SPCC70.05c	-0.90	5.62E-06
SPCC622.01c	-2.09	1.73E-06	SPCC622.06c	-1.12	3.47E-02	SPAC23C4.14	-0.89	6.49E-05
SPCC1902.02	-2.08	2.34E-04	SPAC2E12.03c	-1.12	2.54E-02	SPAC22G7.02	-0.89	6.14E-05
SPAC589.09	-2.08	6.64E-05	SPAC17C9.09c	-1.11	9.45E-03	SPBC2G2.05	-0.89	8.40E-04
SPBC25B2.08	-2.05	3.28E-05	SPAC29B12.14c	-1.11	1.26E-04	SPAC56F8.07	-0.89	2.21E-02
SPCC1682.09c	-2.04	3.76E-05	SPAC13G7.09c	-1.10	2.72E-02	SPCC24B10.13	-0.89	2.42E-03
SPAC1399.04c	-2.03	5.85E-03	SPBC28F2.11	-1.09	7.21E-06	SPAC1851.02	-0.89	4.49E-05
SPAC13G7.13c	-2.02	3.86E-05	SPBC1773.17c	-1.09	8.34E-03	SPBC409.11	-0.89	7.91E-03
SPBC23G7.13c	-2.01	3.79E-02	SPBC4B4.11	-1.09	1.68E-02	SPCC1494.09c	-0.88	4.78E-03
SPCC1223.13	-1.93	5.77E-03	SPBC29A10.13	-1.08	1.96E-04	SPAC23D3.11	-0.88	3.47E-02
SPBP26C9.03c	-1.89	3.18E-02	SPBC216.04c	-1.08	3.21E-02	SPAC24H6.05	-0.88	2.94E-03
SPBC29B5.02c	-1.83	2.38E-05	SPAC1834.07	-1.08	4.95E-04	SPAC3G6.05	-0.88	1.03E-03
SPBC337.09	-1.81	1.10E-07	SPCC1795.07	-1.07	8.83E-05	SPAC1093.01	-0.87	2.68E-03
SPBC21D10.07	-1.80	3.03E-07	SPAC13F5.05	-1.06	6.33E-05	SPAC222.10c	-0.87	2.52E-04
SPAC1834.03c	-1.76	1.68E-05	SPBP4H10.11c	-1.06	5.94E-03	SPBC6B1.02	-0.87	1.34E-03
SPAC17G6.02c	-1.73	3.36E-06	SPAC23H3.13c	-1.05	2.34E-03	SPBC1706.01	-0.87	6.33E-05
SPCC663.09c	-1.68	4.11E-03	SPAC4G9.10	-1.04	3.60E-04	SPAC13G6.15c	-0.86	5.17E-03
SPAC8E11.10	-1.67	4.59E-04	SPBC713.05	-1.04	1.78E-04	SPBC19G7.07c	-0.86	9.20E-04
SPAC23H3.12c	-1.62	2.13E-04	SPAC823.15	-1.04	1.03E-05	SPBC211.01	-0.86	3.14E-03
SPCC1682.08c	-1.59	2.61E-03	SPAC6F12.05c	-1.03	3.67E-05	SPAPB1E7.07	-0.86	1.11E-03
SPAPYUG7.04c	-1.54	3.02E-03	SPAC1782.01	-1.03	1.31E-04	SPCC1620.01c	-0.86	5.31E-03

SPBP4H10.20	-1.53	1.69E-07	SPBC16G5.17	-1.03	1.73E-04	SPBC19C2.08	-0.86	1.61E-02
SPBC359.04c	-1.53	7.69E-04	SPAC17A2.14	-1.03	5.77E-06	SPAC23G3.05c	-0.86	2.90E-02
SPAC5H10.06c	-1.52	2.42E-04	SPBC1105.03c	-1.02	5.53E-04	SPBC577.08c	-0.86	7.90E-04
SPAC1250.04c	-1.50	3.86E-03	SPBC713.12	-1.02	1.10E-02	SPBC2D10.09	-0.85	5.40E-04
SPCC63.03	-1.50	1.71E-02	SPAC5H10.10	-1.02	1.92E-03	SPAC56F8.10	-0.85	1.15E-04
SPAC589.03c	-1.47	1.34E-03	SPBC839.07	-1.01	5.77E-03	SPCC1672.01	-0.85	4.11E-04
SPBC8D2.09c	-1.46	2.16E-02	SPBC3H7.12	-1.01	8.09E-03	SPBC11C11.08	-0.84	1.08E-04
SPBC12D12.07c	-1.45	3.16E-03	SPCC622.12c	-1.01	4.65E-06	SPBC3B9.18c	-0.84	7.74E-03
SPAC27D7.04	-1.44	7.97E-07	SPCC126.10	-1.01	1.11E-04	SPBC31F10.04c	-0.84	1.39E-03
SPBP23A10.12	-1.44	1.45E-04	SPBC29A10.16c	-1.00	2.50E-04	SPBC106.15	-0.83	1.16E-03
SPAC17C9.16c	-1.44	6.63E-05	SPAC2E1P3.04	-1.00	3.43E-03	SPBC651.04	-0.83	3.66E-02
SPCC622.02	-1.43	4.81E-05	SPBC839.03c	-1.00	4.20E-04	SPBC800.11	-0.83	9.79E-03
SPCC622.07	-1.42	2.44E-02	SPBC21C3.04c	-1.00	7.78E-03	SPAC7D4.06c	-0.83	2.23E-03
SPBC725.16	-1.42	6.90E-04	SPCC553.03	-0.99	9.01E-04	SPAC2F7.08c	-0.83	5.91E-03
SPBC1683.02	-1.41	8.47E-03	SPAC227.08c	-0.99	5.28E-03	SPBC4B4.10c	-0.83	2.42E-04
SPBC2G2.15c	-1.39	1.50E-06	SPAC926.05c	-0.99	3.64E-02	SPAC1039.04	-0.83	2.19E-02
SPBC26H8.03	-1.38	4.54E-07	SPAC20G4.04c	-0.99	3.57E-02	SPBC17D11.03c	-0.83	1.48E-03
SPBC119.06	-1.37	5.24E-05	SPAP7G5.04c	-0.98	9.50E-05	SPAC19B12.12c	-0.83	1.07E-02
SPBC19F8.05	-1.36	8.26E-04	SPAC328.09	-0.98	2.24E-02	SPAC3C7.10	-0.82	3.96E-03
SPBC1683.05	-1.36	5.44E-04	SPAC1805.16c	-0.98	2.53E-04	SPAC664.13	-0.82	4.56E-03
SPAC24H6.11c	-1.35	1.60E-04	SPCC320.06	-0.98	5.99E-03	SPBC19F5.03	-0.82	2.18E-03
SPBP22H7.06	-1.34	3.46E-06	SPAC13G6.06c	-0.98	2.45E-02	SPBC365.01	-0.82	7.14E-03
SPAC1834.05	-1.31	8.91E-07	SPAC1002.18	-0.98	4.42E-02	SPAC22F3.07c	-0.81	2.59E-03
SPAC8C9.05	-1.29	2.25E-03	SPAC22A12.11	-0.97	2.57E-03	SPAPB1A10.03	-0.81	1.33E-03
SPBC2G2.01c	-1.28	5.78E-04	SPBC211.06	-0.97	1.09E-03	SPBC1A4.04	-0.81	3.84E-03
SPAC1B3.10c	-1.27	3.22E-02	SPCC1919.13c	-0.97	1.04E-02	SPBC1198.06c	-0.81	1.14E-03
SPCC1259.09c	-1.27	1.95E-05	SPBC428.04	-0.97	2.99E-03	SPAC3H1.04c	-0.81	1.92E-03
SPCC191.03c	-1.26	1.69E-03	SPBC1734.02c	-0.96	1.90E-02	SPAC2H10.02c	-0.81	5.90E-03
SPBC3H7.07c	-1.25	4.55E-03	SPBC13E7.11	-0.96	2.45E-04	SPAC10F6.07c	-0.80	1.63E-02
SPBC691.04	-1.25	2.31E-04	SPAC1399.02	-0.96	7.98E-04	SPAC2E1P5.03	-0.80	8.31E-03
			SPCC70.03c	-0.95	4.19E-02	SPAC19B12.07c	-0.80	1.85E-02

SPBC36.03c	-0.80	1.85E-02
SPBC11B10.03	-0.80	2.07E-02

	*mst1^ts^*			*mst1^ts^*			*mst1^ts^*	
Gene	Log2 Change	pvalue	Gene	Log2 Change	pvalue	Gene	Log2 Change	pvalue
SPAC1039.02	-2.66	6.36E-04	SPACUNK4.14	-1.12	4.95E-05	SPBP35G2.03c	-0.92	6.48E-04
SPBC29A3.05	-2.18	5.55E-05	SPCC550.02c	-1.12	2.19E-06	SPAC17C9.08	-0.92	7.87E-09
SPCC757.06	-2.08	1.87E-07	SPAC589.09	-1.12	9.76E-04	SPACUNK4.06c	-0.91	6.36E-07
SPAC6B12.09	-1.86	5.44E-06	SPAC7D4.05	-1.12	3.57E-04	SPCC576.13	-0.91	4.94E-05
SPBC947.04	-1.85	2.36E-04	SPBC31F10.12	-1.11	1.01E-04	SPAPB2B4.06	-0.91	7.04E-05
SPAC4H3.06	-1.78	1.49E-05	SPBP8B7.31	-1.11	1.61E-03	SPAC4G9.13c	-0.91	2.44E-03
SPBC14F5.01	-1.77	4.08E-05	SPCC663.09c	-1.11	6.60E-03	SPAC3G9.02	-0.91	1.56E-04
SPAC1486.08	-1.74	5.14E-04	SPBC4B4.05	-1.11	5.68E-03	SPAC227.02c	-0.91	1.21E-02
SPCC338.04	-1.68	9.00E-04	SPBC30B4.02c	-1.11	2.43E-04	SPBC577.03c	-0.91	5.32E-03
SPAC17G8.09	-1.59	3.35E-04	SPCC613.07	-1.11	2.43E-05	SPAC8C9.05	-0.91	2.39E-03
SPAC1F12.08	-1.59	1.53E-07	SPBC1683.06c	-1.10	3.69E-02	SPBC16E9.19	-0.91	8.95E-06
SPAC8C9.07	-1.59	1.14E-03	SPBC1861.01c	-1.10	2.77E-02	SPBC11G11.02c	-0.91	2.94E-07
SPBC146.08c	-1.59	2.04E-04	SPAC1F12.03c	-1.10	1.13E-02	SPCC1183.03c	-0.91	9.54E-04
SPBC365.11	-1.58	6.64E-04	SPBP18G5.02	-1.10	2.23E-05	SPBC27.05	-0.91	6.85E-03
SPCC1919.07	-1.58	3.99E-05	SPBC16E9.06c	-1.10	1.55E-04	SPBC839.03c	-0.90	3.04E-05
SPAC1250.04c	-1.57	1.21E-04	SPBC27B12.07	-1.09	7.80E-07	SPAC13G6.14	-0.90	5.25E-04
SPAC2C4.12c	-1.56	2.82E-06	SPBC365.05c	-1.09	5.07E-05	SPBC16A3.06	-0.90	1.34E-04
SPAC2E1P3.05c	-1.56	4.99E-08	SPBC1861.07	-1.08	9.22E-05	SPAC17A5.13	-0.90	1.85E-04
SPBC2A9.12	-1.53	1.99E-03	SPAC1F12.09	-1.08	7.93E-10	SPCC736.03c	-0.90	1.69E-05
SPBC3D6.09	-1.53	3.75E-05	SPBC1861.04c	-1.08	2.46E-05	SPAC17A2.14	-0.90	3.15E-07
SPAC139.06	-1.52	2.60E-06	SPAC15E1.08	-1.08	1.94E-04	SPAC19G12.13c	-0.90	7.47E-04
SPBC19G7.02	-1.52	1.02E-04	SPAC22F3.11c	-1.08	1.22E-02	SPAC2C4.14c	-0.90	1.09E-04
SPAC3G6.06c	-1.51	5.65E-06	SPAC926.05c	-1.08	2.65E-03	SPAC9.12c	-0.90	5.36E-07

SPBC336.08	-1.51	6.85E-07	SPAC664.08c	-1.08	2.57E-03	SPBC713.09	-0.90	2.54E-03
SPAC12G12.02	-1.51	3.99E-05	SPBC36.12c	-1.08	5.12E-05	SPAC13G7.09c	-0.90	1.26E-02
SPBC365.04c	-1.49	3.84E-04	SPAC25B8.15c	-1.07	2.24E-05	SPBC725.13c	-0.90	8.60E-04
SPBC3B9.21	-1.49	1.89E-03	SPAC688.12c	-1.07	6.45E-04	SPCC1827.01c	-0.90	1.10E-02
SPBC16A3.16	-1.47	5.79E-03	SPCC4G3.16	-1.07	1.10E-02	SPBC14F5.08	-0.90	1.92E-06
SPCC16C4.20c	-1.45	5.35E-04	SPAC2F7.17	-1.07	5.19E-05	SPBC36.05c	-0.90	8.63E-09
SPAC13G7.07	-1.45	4.54E-06	SPBC83.08	-1.07	2.61E-08	SPCC1795.01c	-0.90	3.27E-05
SPAC17A5.01	-1.43	5.74E-08	SPAC16E8.04c	-1.07	9.47E-07	SPBC16G5.17	-0.90	1.67E-05
SPAC1B3.10c	-1.42	1.83E-03	SPBC3H7.07c	-1.07	1.03E-03	SPCC126.10	-0.89	7.93E-06
SPAC630.06c	-1.42	1.73E-04	SPAC227.16c	-1.07	7.74E-05	SPAC20G4.04c	-0.89	9.54E-03
SPBC56F2.14	-1.42	4.23E-03	SPAC56E4.07	-1.07	3.96E-06	SPAC12B10.10	-0.89	4.41E-02
SPAC1952.02	-1.41	4.41E-05	SPBC30D10.12c	-1.07	5.52E-04	SPCC622.06c	-0.89	1.93E-02
SPBC4B4.12c	-1.41	5.35E-07	SPAC3F10.08c	-1.06	2.87E-03	SPAC1A6.02	-0.89	3.63E-05
SPCC663.11	-1.40	7.08E-06	SPBC20F10.04c	-1.06	6.65E-07	SPBC24C6.07	-0.89	8.63E-05
SPAC8C9.10c	-1.40	2.19E-04	SPAC14C4.02c	-1.06	5.40E-05	SPAC823.08c	-0.89	3.20E-03
SPAC57A10.03	-1.40	2.96E-09	SPAC1002.07c	-1.06	1.36E-05	SPAC31A2.02	-0.89	3.78E-04
SPAC227.05	-1.40	3.88E-03	SPBC19F8.01c	-1.06	1.23E-05	SPAC7D4.02c	-0.89	5.35E-06
SPCC663.13c	-1.39	1.56E-03	SPBC1734.05c	-1.06	8.63E-04	SPAC227.09	-0.89	9.78E-05
SPCC1840.10	-1.39	1.92E-04	SPBC32F12.15	-1.06	2.09E-02	SPAC23H4.07c	-0.89	4.39E-03
SPACUNK4.13c	-1.38	3.25E-07	SPBC19C7.01	-1.06	1.34E-04	SPCC830.10	-0.89	1.87E-05
SPCC2H8.04	-1.38	1.91E-04	SPCC1919.14c	-1.06	1.87E-04	SPAC1142.06	-0.88	1.51E-09
SPAC1D4.01	-1.38	1.49E-03	SPAC4C5.04	-1.05	2.90E-07	SPBC31F10.04c	-0.88	2.34E-05
SPAC24C9.09	-1.37	3.72E-07	SPAC10F6.08c	-1.05	3.86E-05	SPAC23C4.09c	-0.88	1.13E-02
SPAC31G5.14	-1.36	7.08E-07	SPBC119.15	-1.05	4.40E-06	SPBC3B8.01c	-0.88	1.26E-05
SPCC63.07	-1.36	3.29E-04	SPACUNK4.09	-1.05	2.18E-04	SPBC649.03	-0.88	7.34E-03
SPAP8A3.02c	-1.35	1.57E-03	SPBC21.03c	-1.05	5.86E-04	SPAPB24D3.06c	-0.88	4.01E-05
SPBC36B7.08c	-1.35	9.15E-05	SPBC2A9.09	-1.05	4.13E-05	SPBC19C2.13c	-0.88	1.03E-03
SPAC11E3.03	-1.35	4.04E-03	SPBC29A10.16c	-1.05	2.81E-06	SPBC8D2.13	-0.88	1.16E-05
SPBP8B7.02	-1.34	3.30E-04	SPCC162.05	-1.04	3.60E-05	SPAC20H4.05c	-0.88	5.09E-05
SPAC22A12.14c	-1.34	2.00E-04	SPAC17D4.04	-1.04	7.46E-06	SPAC16E8.06c	-0.88	3.79E-03
SPAC1F12.06c	-1.33	1.35E-07	SPCC1223.13	-1.04	2.78E-02	SPAC4F8.04	-0.88	8.23E-03

SPBC887.08	-1.33	2.75E-02	SPAC29A4.14c	-1.04	7.15E-05	SPBC21D10.07	-0.88	3.69E-05
SPBC25H2.10c	-1.33	8.84E-07	SPAC1782.03	-1.04	3.22E-03	SPBC25B2.04c	-0.88	1.77E-03
SPBC19F8.02	-1.33	5.75E-04	SPBC1271.07c	-1.04	2.98E-05	SPAC4G8.10	-0.88	1.70E-04
SPCP1E11.07c	-1.33	5.31E-03	SPAC12B10.04	-1.04	4.00E-04	SPAC343.03	-0.87	1.21E-05
SPBC21B10.13c	-1.33	4.92E-04	SPAC3A11.13	-1.03	4.06E-03	SPAC27D7.02c	-0.87	7.45E-04
SPAC8C9.17c	-1.32	2.06E-07	SPBC1709.07	-1.03	2.93E-05	SPAC25G10.07c	-0.87	2.09E-05
SPCC162.01c	-1.31	1.43E-03	SPAC9.06c	-1.03	1.30E-05	SPBC3B9.12	-0.87	9.26E-03
SPBC1703.08c	-1.31	1.95E-03	SPBC1709.19c	-1.03	3.72E-05	SPBC1703.01c	-0.87	3.33E-03
SPAC4H3.14c	-1.31	1.12E-05	SPCC11E10.04	-1.03	5.47E-06	SPBC15C4.01c	-0.87	1.15E-06
SPBC1703.05	-1.31	6.69E-04	SPAC110.02	-1.02	1.66E-05	SPAC1002.10c	-0.87	3.24E-03
SPAC1952.03	-1.30	4.62E-04	SPAC13G6.05c	-1.02	2.24E-05	SPAC589.02c	-0.87	2.05E-03
SPBC354.04	-1.30	8.85E-07	SPBC23G7.07c	-1.02	2.54E-02	SPCC1739.07	-0.87	1.07E-02
SPAC688.06c	-1.30	2.58E-06	SPAC1F8.06	-1.02	3.79E-03	SPBP23A10.12	-0.87	6.95E-04
SPAC1F3.01	-1.30	1.88E-04	SPBC14C8.15	-1.02	3.69E-07	SPCC24B10.19c	-0.87	3.35E-04
SPAC11E3.08c	-1.30	8.33E-06	SPBC1683.02	-1.02	7.47E-03	SPAC31G5.15	-0.87	1.96E-04
SPCC1827.04	-1.29	3.90E-04	SPCC16A11.12c	-1.02	4.75E-04	SPBC6B1.04	-0.86	6.36E-06
SPCC613.11c	-1.29	3.23E-06	SPBC13G1.03c	-1.02	5.29E-03	SPBC2D10.16	-0.86	2.70E-02
SPAC27F1.04c	-1.29	1.18E-03	SPCP20C8.02c	-1.01	1.36E-02	SPAC17C9.05c	-0.86	1.44E-06
SPBC29A3.07c	-1.28	2.86E-03	SPBC19C7.07c	-1.01	5.15E-04	SPCC4B3.08	-0.86	2.68E-04
SPBC405.05	-1.28	1.62E-03	SPAC227.17c	-1.01	2.86E-05	SPAC23G3.10c	-0.86	4.18E-05
SPAC144.01	-1.27	3.79E-02	SPAC227.12	-1.01	2.21E-05	SPBP8B7.01c	-0.86	5.09E-03
SPBC27B12.02	-1.27	2.30E-02	SPBP8B7.08c	-1.01	7.14E-04	SPAC13A11.03	-0.86	2.45E-02
SPBC32F12.05c	-1.26	2.65E-05	SPBC27.04	-1.01	1.46E-02	SPBC365.09c	-0.86	6.34E-05
SPAC11D3.02c	-1.26	4.53E-05	SPAC167.08	-1.00	1.05E-03	SPCC1840.11	-0.86	3.25E-04
SPCC16A11.05c	-1.26	3.53E-04	SPBP16F5.05c	-1.00	4.81E-03	SPBC887.18c	-0.86	1.98E-04
SPAC1556.04c	-1.25	5.59E-04	SPCC330.03c	-1.00	9.84E-04	SPCC18B5.06	-0.85	5.07E-05
SPBC20F10.09	-1.25	7.69E-05	SPCC645.10	-1.00	1.67E-07	SPCC777.17c	-0.85	2.52E-02
SPAC1039.08	-1.25	6.38E-04	SPAC3A12.11c	-0.99	1.73E-03	SPBC26H8.03	-0.85	2.78E-06
SPBC13E7.06	-1.25	1.27E-04	SPAC4F8.01	-0.99	6.42E-05	SPBC1271.04c	-0.85	1.18E-05
SPAC10F6.10	-1.25	2.27E-03	SPCC825.02	-0.99	1.13E-07	SPBC1539.10	-0.85	3.03E-02
SPBC21C3.07c	-1.24	8.14E-09	SPCC126.03	-0.99	2.73E-03	SPAC4F8.05c	-0.85	2.56E-02

SPBC14C8.12	-1.24	5.41E-05	SPBC1685.11	-0.99	1.54E-04	SPAC27E2.02	-0.85	4.32E-05
SPAC15A10.12c	-1.24	1.01E-03	SPBC30D10.08	-0.99	2.15E-04	SPAC3H1.14	-0.85	8.08E-03
SPAC683.02c	-1.24	4.70E-03	SPBC1D7.01	-0.99	3.15E-03	SPBC1105.15c	-0.85	1.17E-04
SPAC26F1.12c	-1.23	7.06E-04	SPCC2H8.05c	-0.98	5.27E-06	SPAC4F10.19c	-0.84	7.74E-04
SPACUNK4.11c	-1.23	5.88E-03	SPAC31A2.08	-0.98	3.13E-03	SPBC1271.13	-0.84	9.26E-05
SPAC23H4.04	-1.22	6.60E-04	SPAC6G9.06c	-0.98	1.95E-04	SPCC24B10.12	-0.84	5.37E-03
SPCC825.04c	-1.22	1.18E-06	SPBC2D10.13	-0.98	5.31E-04	SPAC16A10.02	-0.84	4.32E-03
SPCC1393.05	-1.22	3.92E-06	SPBC3B9.08c	-0.98	3.31E-03	SPAC1142.04	-0.84	6.78E-03
SPAC644.04	-1.22	4.86E-05	SPBC2D10.15c	-0.98	1.12E-04	SPCC16A11.16c	-0.84	3.93E-05
SPAC5H10.11	-1.21	1.16E-06	SPBC365.13c	-0.98	1.34E-04	SPAC1B3.02c	-0.84	1.36E-03
SPAC688.02c	-1.21	3.46E-04	SPBC1706.03	-0.98	1.66E-05	SPAC6F12.08c	-0.84	9.25E-05
SPBC2G2.05	-1.20	4.82E-07	SPAC323.01c	-0.98	1.34E-05	SPAC823.04	-0.84	3.49E-02
SPBC16H5.15	-1.20	1.27E-04	SPBC31F10.04c	-0.98	2.62E-06	SPCP31B10.03c	-0.83	1.98E-03
SPAC607.02c	-1.20	1.02E-02	SPBC577.15c	-0.98	3.45E-07	SPBC11B10.04c	-0.83	1.97E-03
SPCC126.14	-1.20	1.98E-07	SPAC222.03c	-0.98	6.88E-03	SPAC26H5.06	-0.83	2.68E-05
SPAC3H1.03	-1.20	1.38E-02	SPAC1D4.09c	-0.97	2.08E-04	SPBC15C4.03	-0.83	1.42E-05
SPAC17A2.08c	-1.20	1.00E-04	SPBP22H7.05c	-0.97	1.50E-04	SPBC3H7.14	-0.83	4.85E-03
SPBC31E1.03	-1.20	1.90E-02	SPBC1709.04c	-0.97	3.65E-05	SPCC4B3.16	-0.83	1.42E-03
SPBC12D12.08c	-1.19	1.45E-02	SPAC12B10.06c	-0.97	8.20E-03	SPCC4G3.02	-0.83	1.47E-05
SPBC1271.08c	-1.19	4.44E-02	SPCC757.05c	-0.97	1.50E-08	SPBC4B4.01c	-0.82	8.28E-05
SPBC14C8.09c	-1.19	3.13E-06	SPCC61.02	-0.97	1.41E-02	SPAC27E2.06c	-0.82	1.59E-05
SPBC83.15	-1.19	1.72E-04	SPAC15A10.08	-0.96	2.65E-05	SPBC1718.03	-0.82	1.73E-02
SPCC1223.04c	-1.19	2.86E-06	SPAC20H4.03c	-0.96	8.98E-08	SPAC688.09	-0.82	3.42E-05
SPCC1393.09c	-1.19	4.12E-05	SPAC4F8.03	-0.96	8.04E-05	SPBC543.03c	-0.82	2.14E-06
SPAP27G11.04c	-1.18	3.54E-05	SPAC458.07	-0.96	4.48E-05	SPBC16G5.12c	-0.82	3.80E-03
SPCC553.07c	-1.18	3.02E-06	SPAC1834.10c	-0.96	7.76E-04	SPBC3B9.02c	-0.82	3.00E-04
SPAC644.10	-1.17	1.99E-04	SPAC3A12.09c	-0.96	5.52E-04	SPBC20F10.05	-0.82	7.94E-06
SPAC1F5.07c	-1.17	6.26E-06	SPBC215.02	-0.96	7.65E-03	SPBC336.13c	-0.82	1.02E-04
SPCC16C4.05	-1.17	2.28E-04	SPBC20F10.06	-0.96	7.46E-03	SPBC1773.17c	-0.81	5.91E-03
SPAC688.14	-1.16	3.53E-05	SPBP4H10.20	-0.95	9.88E-07	SPAC167.04	-0.81	1.89E-05
SPAC12G12.16c	-1.16	2.76E-06	SPAC3H8.07c	-0.95	3.10E-03	SPAC12G12.13c	-0.81	6.70E-04

SPAC323.06c	-1.16	4.87E-04	SPAC11E3.12	-0.95	3.07E-04	SPAC29E6.03c	-0.81	9.24E-03
SPCC569.07	-1.16	4.10E-04	SPBC1703.14c	-0.95	5.27E-04	SPCC297.06c	-0.81	1.26E-02
SPCC613.03	-1.16	6.64E-06	SPAC823.05c	-0.95	8.63E-05	SPAC24H6.08	-0.81	1.15E-04
SPCC830.11c	-1.16	1.36E-04	SPBC31E1.05	-0.95	3.45E-05	SPAC17H9.06c	-0.81	1.72E-03
SPBC11C11.01	-1.16	5.34E-05	SPAC4A8.09c	-0.95	2.52E-04	SPBC776.14	-0.81	1.83E-04
SPAPB1E7.10	-1.16	2.88E-03	SPAC29A4.06c	-0.95	8.85E-04	SPAC922.06	-0.81	3.17E-03
SPAC8E11.07c	-1.16	2.14E-03	SPAC11H11.03c	-0.95	3.32E-04	SPBC2D10.11c	-0.81	2.16E-05
SPBC1539.02	-1.16	2.14E-04	SPBC3E7.11c	-0.95	4.13E-06	SPBC30D10.02	-0.81	4.46E-04
SPCPB16A4.04c	-1.16	1.97E-05	SPBC18H10.15	-0.95	3.22E-06	SPBC947.07	-0.81	1.03E-02
SPBC409.11	-1.16	3.18E-05	SPAC2F7.15	-0.94	1.04E-05	SPBP35G2.04c	-0.81	7.35E-03
SPAC664.12c	-1.15	2.26E-04	SPCC645.02	-0.94	8.83E-03	SPAC11H11.05c	-0.81	7.11E-04
SPAC56F8.09	-1.15	7.35E-04	SPAC589.08c	-0.94	5.32E-03	SPCC622.14	-0.81	5.55E-05
SPAC1687.13c	-1.15	7.64E-07	SPBC12D12.07c	-0.94	6.14E-03	SPAP8A3.10	-0.81	2.83E-03
SPCC1442.13c	-1.14	1.68E-04	SPCC1753.04	-0.93	1.25E-08	SPAC13G6.04	-0.81	2.58E-02
SPBC1347.04	-1.14	2.56E-04	SPAC12B10.05	-0.93	9.86E-07	SPAC1071.04c	-0.80	2.83E-02
SPBC3D6.04c	-1.14	5.44E-04	SPBC29A10.12	-0.93	1.09E-03	SPCC737.06c	-0.80	2.44E-04
SPBP8B7.28c	-1.14	4.21E-03	SPCC1919.13c	-0.93	1.10E-03	SPBC405.06	-0.80	8.56E-05
SPBC1347.01c	-1.14	1.22E-03	SPAPB1E7.11c	-0.93	1.76E-04	SPAC20H4.08	-0.80	3.23E-02
SPBC3B9.10	-1.13	1.19E-05	SPAC630.10	-0.93	1.72E-05	SPBC9B6.05c	-0.80	1.66E-04
SPBC354.03	-1.13	7.44E-05	SPBC17A3.08	-0.93	3.80E-09	SPAC57A10.11c	-0.80	1.89E-04
SPAC27D7.07c	-1.13	1.41E-04	SPAC10F6.07c	-0.93	4.19E-04	SPCC1919.02	-0.80	5.13E-04
SPBC1734.02c	-1.13	4.70E-04	SPCC962.05	-0.92	1.14E-06	SPCC645.12c	-0.80	1.45E-04
SPBC6B1.12c	-1.12	1.87E-03	SPBC776.12c	-0.92	2.75E-04			
SPCC613.12c	-1.12	9.16E-06	SPBC725.17c	-0.92	1.47E-05			
SPBC19G7.04	-1.12	2.57E-03	SPBC1105.18c	-0.92	2.61E-02			
SPBC16C6.05	-1.12	6.84E-04	SPAC23C4.02	-0.92	2.06E-02			
			SPBC16A3.12c	-0.92	6.95E-09			
			SPAC17H9.13c	-0.92	6.40E-06			
			SPAC4A8.05c	-0.92	2.14E-03			

Up-regulated

Δelp3

Gene	Log2 Change	pvalue	Gene	Log2 Change	pvalue	Gene	Log2 Change	pvalue
SPBCPT2R1.08c	4.50	1.90E-04	SPCC584.02	1.18	2.64E-05	SPBC19G7.13	0.93	2.54E-05
SPAC977.16c	3.86	3.35E-10	SPAC343.07	1.18	4.11E-05	SPAC11D3.09	0.93	3.75E-04
SPAC750.07c	3.83	1.50E-08	SPCC70.08c	1.17	5.62E-04	SPAPB15E9.02c	0.92	9.68E-04
SPAC186.01	3.35	3.47E-10	SPAC57A7.05	1.17	1.78E-02	SPBC3B8.09	0.92	3.31E-03
SPAC212.08c	2.97	1.73E-14	SPBC27.03	1.16	3.44E-06	SPCC13B11.04c	0.92	6.37E-04
SPAC2E1P3.02c	2.62	2.24E-09	SPCC162.02c	1.15	3.38E-07	SPCC162.03	0.92	6.07E-05
SPAC186.02c	2.30	3.71E-09	SPBC6B1.03c	1.15	1.56E-05	SPAC3F10.10c	0.92	3.13E-05
SPAC869.01	2.29	2.04E-12	SPCC569.07	1.15	4.69E-04	SPAC1039.04	0.92	9.91E-04
SPAC6C3.03c	2.25	1.97E-07	SPAP7G5.03	1.15	9.86E-07	SPAC15E1.02c	0.92	6.58E-03
SPCC1393.10	2.22	1.28E-07	SPCC1672.03c	1.14	9.29E-05	SPAC1039.01	0.91	2.98E-02
SPBPB2B2.01	2.20	1.27E-04	SPBC16E9.10c	1.14	8.08E-05	SPAC1006.03c	0.91	1.72E-05
SPBC23G7.10c	2.17	1.62E-02	SPAC1F8.05	1.14	5.70E-03	SPAC21E11.04	0.91	4.13E-04
SPAC24C9.15c	2.17	8.66E-05	SPBC8E4.03	1.12	1.48E-04	SPAC56E4.06c	0.91	4.17E-06
SPAC750.04c	2.08	4.04E-07	SPBC1683.01	1.12	5.83E-05	SPAC6F6.04c	0.91	1.04E-06
SPBC359.02	2.08	3.34E-09	SPBC3D6.11c	1.11	4.91E-06	SPAC7D4.08	0.90	5.57E-03
SPBC1778.04	2.05	1.97E-08	SPAC8C9.10c	1.11	1.83E-03	SPAC29A4.19c	0.90	1.19E-07
SPBPB8B6.03	2.05	2.21E-08	SPBC29A10.02	1.11	1.05E-04	SPAC27D7.13c	0.90	1.69E-05
SPBC2G2.17c	2.02	8.36E-06	SPAPB24D3.02c	1.11	9.64E-08	SPBC11G11.05	0.90	1.36E-02
SPCC132.04c	2.01	3.61E-06	SPBC29A10.14	1.11	1.96E-05	SPAC323.04	0.89	3.46E-04
SPBC32H8.11	1.93	3.06E-06	SPBC13E7.02	1.10	5.34E-06	SPCC895.06	0.89	1.85E-04
SPAPB1A11.01	1.88	4.70E-07	SPBC947.13	1.10	3.93E-05	SPCC550.10	0.89	2.11E-05
SPBC1861.06c	1.87	1.43E-06	SPAC6G9.01c	1.10	1.00E-03	SPBC23G7.11	0.88	1.16E-02
SPAC11H11.04	1.86	1.93E-02	SPBC1347.03	1.09	1.06E-04	SPAC16.04	0.88	3.77E-04
SPAC10F6.15	1.85	3.86E-08	SPAC11D3.05	1.08	9.83E-03	SPAC5H10.04	0.88	8.05E-03
SPAC56F8.12	1.83	1.07E-07	SPCC790.02	1.08	6.01E-08	SPAC3C7.13c	0.88	2.24E-05
SPCC830.04c	1.77	3.11E-06	SPBC359.04c	1.08	7.84E-04	SPBC651.01c	0.88	1.02E-03
SPBC31F10.08	1.76	3.63E-05	SPAC29B12.12	1.08	4.98E-04	SPBC28E12.02	0.88	4.93E-04

SPBC354.08c	1.75	5.80E-06	SPCC285.09c	1.08	2.49E-04	SPAC11E3.09	0.88	2.33E-04
SPBC21C3.02c	1.73	3.75E-08	SPAC922.07c	1.07	5.34E-04	SPAC4F10.15c	0.87	6.93E-04
SPBC8E4.02c	1.73	3.53E-08	SPAC25H1.09	1.07	9.45E-05	SPAC3C7.04	0.87	3.33E-06
SPBC146.11c	1.70	3.31E-08	SPBC359.01	1.07	2.11E-05	SPAC15A10.10	0.87	2.20E-04
SPAC2H10.01	1.68	3.07E-02	SPCC18.10	1.06	2.66E-07	SPAC17A5.04c	0.87	6.79E-07
SPAC32A11.01	1.65	6.28E-04	SPCC2H8.02	1.06	3.34E-06	SPBC1652.01	0.87	5.24E-03
SPCC330.07c	1.62	4.64E-08	SPBC1685.12c	1.06	9.17E-03	SPBC26H8.08c	0.87	5.46E-03
SPBC36.01c	1.57	1.78E-05	SPAC31A2.12	1.06	4.71E-06	SPAC328.08c	0.87	3.86E-03
SPCC74.02c	1.57	1.52E-07	SPCC11E10.01	1.06	8.56E-06	SPCC613.12c	0.87	1.77E-04
SPBC1685.13	1.56	1.51E-02	SPBC19F5.02c	1.05	1.22E-04	SPBC1711.07	0.86	6.60E-03
SPCC417.04	1.55	5.01E-09	SPAC9E9.12c	1.05	1.39E-06	SPBC557.02c	0.86	9.61E-06
SPBC1861.01c	1.55	3.44E-03	SPCC1827.01c	1.05	3.92E-03	SPBC1347.12	0.86	3.15E-05
SPAC977.17	1.54	8.49E-06	SPCC290.02	1.04	2.94E-04	SPAC637.11	0.86	1.19E-04
SPBPB8B6.04c	1.52	3.79E-04	SPAC2E1P3.04	1.04	1.08E-04	SPCC1494.06c	0.86	2.63E-03
SPBC317.01	1.52	2.25E-05	SPCC553.01c	1.03	5.72E-09	SPBC1604.01	0.86	1.93E-05
SPAC1142.05	1.51	8.79E-07	SPCC1450.07c	1.03	1.79E-04	SPAC14C4.02c	0.85	5.06E-04
SPAPB1A10.08	1.48	1.68E-07	SPBC3D6.03c	1.03	5.04E-04	SPAC3C7.02c	0.85	1.09E-03
SPAPB18E9.03c	1.47	2.97E-04	SPBPB21E7.10	1.03	4.48E-02	SPAC4G9.10	0.85	7.81E-05
SPBC8D2.19	1.44	9.74E-07	SPAC4H3.11c	1.03	9.08E-05	SPBC1D7.05	0.85	4.24E-03
SPBCPT2R1.02	1.42	3.18E-05	SPBC30B4.01c	1.03	1.35E-02	SPAC56F8.14c	0.85	1.14E-02
SPBC947.04	1.41	2.62E-03	SPAC11D3.06	1.02	2.26E-07	SPBC17D1.07c	0.85	6.63E-03
SPBC1685.06	1.41	5.92E-05	SPAC6C3.02c	1.01	2.40E-03	SPBP35G2.03c	0.85	1.32E-03
SPAC1006.04c	1.38	1.28E-05	SPCC162.04c	1.00	2.14E-04	SPBC106.08c	0.84	5.05E-04
SPAC4G9.05	1.38	5.05E-05	SPBC713.06	1.00	2.60E-04	SPAC140.02	0.84	1.05E-02
SPAC4F10.09c	1.38	4.66E-06	SPAC8E11.03c	1.00	4.80E-03	SPBC30B4.02c	0.84	2.75E-03
SPBC9B6.03	1.37	4.59E-04	SPBC2G2.15c	1.00	1.26E-06	SPAC5H10.07	0.84	1.37E-03
SPBC18E5.10	1.37	1.93E-06	SPBP8B7.18c	1.00	5.53E-06	SPAC11D3.03c	0.84	1.28E-02
SPAC144.09c	1.37	2.09E-06	SPAC19G12.01c	0.99	4.59E-08	SPAC3A11.09	0.84	4.19E-05
SPBC215.13	1.36	3.67E-08	SPAC22F8.11	0.99	4.49E-07	SPCC417.03	0.84	2.59E-05
SPCC965.12	1.36	3.34E-05	SPAC328.09	0.99	2.22E-03	SPBC1198.12	0.84	6.15E-04
SPBPB2B2.18	1.35	1.78E-02	SPBC582.06c	0.99	2.09E-06	SPAC890.05	0.84	5.02E-03

SPCC576.17c	1.34	2.81E-06	SPAC2E1P5.05	0.99	7.15E-04	SPAC29A4.10	0.83	2.39E-03
SPAC1F8.04c	1.34	1.11E-02	SPAC57A7.06	0.99	1.28E-03	SPCC330.09	0.83	1.70E-04
SPACUNK4.09	1.34	1.48E-05	SPBC119.17	0.99	1.91E-04	SPBC21H7.04	0.83	1.21E-03
SPCC965.11c	1.34	2.06E-04	SPBC16A3.17c	0.98	1.89E-03	SPAC18B11.03c	0.83	1.41E-02
SPAPB24D3.07c	1.33	5.52E-02	SPAC1805.03c	0.98	7.31E-06	SPBC4F6.07c	0.83	2.98E-04
SPCC965.14c	1.33	6.05E-03	SPBP8B7.04	0.98	8.34E-05	SPAC6G10.06	0.83	3.45E-05
SPBC56F2.03	1.32	6.49E-07	SPAC1527.03	0.97	1.48E-04	SPBC2G2.01c	0.83	1.31E-03
SPBC1348.01	1.32	3.43E-02	SPAC1002.16c	0.97	6.08E-04	SPBC530.07c	0.83	3.35E-05
SPCC1919.14c	1.30	1.83E-05	SPAC57A10.04	0.97	1.50E-05	SPCC18.05c	0.83	1.11E-04
SPAC4F10.08	1.29	9.03E-07	SPAC6G9.12	0.96	2.57E-05	SPBP23A10.11c	0.82	3.27E-05
SPCC757.11c	1.29	3.59E-05	SPAC3G6.04	0.96	1.22E-03	SPAC23D3.01	0.82	2.51E-05
SPAC1250.01	1.29	1.17E-06	SPBC13A2.04c	0.96	5.43E-02	SPCC1020.05	0.82	3.69E-03
SPBC24C6.02	1.28	7.38E-05	SPAC3A11.07	0.96	1.08E-02	SPBC3D6.12	0.82	2.00E-04
SPCC1020.09	1.28	4.64E-03	SPBC2G5.03	0.96	7.12E-03	SPBC6B1.05c	0.82	5.12E-05
SPAC11D3.01c	1.25	1.07E-02	SPAC22F8.09	0.96	2.89E-02	SPAC17A2.12	0.82	2.45E-03
SPBC14C8.01c	1.24	3.74E-04	SPAC19A8.07c	0.96	2.80E-03	SPCC320.08	0.81	1.71E-03
SPAPB18E9.04c	1.23	1.20E-04	SPCC1223.02	0.96	3.43E-04	SPAC26A3.12c	0.81	1.09E-04
SPBC660.15	1.23	5.51E-05	SPBC19C7.03	0.95	7.54E-04	SPAC1B3.15c	0.81	1.47E-03
SPCC663.14c	1.22	2.00E-06	SPAC11H11.03c	0.95	3.05E-04	SPBC119.14	0.81	1.08E-04
SPAC977.07c	1.21	4.27E-05	SPBC21D10.08c	0.95	2.84E-02	SPAC977.03	0.81	1.13E-03
SPAC9.10	1.21	8.22E-06	SPBC18H10.09	0.95	5.73E-06	SPAC24H6.13	0.81	5.39E-03
SPAC926.08c	1.21	2.07E-03	SPCC18.13	0.95	1.30E-03	SPCC16C4.14c	0.81	6.87E-04
SPAC2C4.06c	1.21	1.38E-05	SPAC167.08	0.95	1.66E-03	SPBC19C2.11c	0.81	1.36E-03
SPAC26H5.06	1.21	2.04E-07	SPBC12C2.03c	0.94	1.30E-04	SPCP20C8.02c	0.81	4.25E-02
SPCC330.03c	1.20	1.63E-04	SPBC21C3.10c	0.94	2.46E-02	SPBC17D1.01	0.81	4.49E-04
SPAC1F12.10c	1.20	5.05E-02	SPBC28F2.12	0.94	3.76E-04	SPAC3F10.16c	0.80	4.48E-04
SPAC8C9.11	1.20	1.49E-04	SPCC962.02c	0.94	1.79E-04	SPBC21B10.12	0.80	1.04E-05
SPAC1952.15c	1.19	1.25E-05	SPAC869.11	0.94	2.42E-04	SPCC622.19	0.80	4.22E-04
SPAC869.02c	1.19	3.86E-03	SPAC1527.01	0.93	3.77E-05	SPCC63.06	0.80	5.30E-05
SPAC6C3.05	1.19	3.90E-05	SPBC29A3.11c	0.93	7.67E-04	SPBC3F6.04c	0.80	5.89E-03
SPBC83.12	1.19	1.95E-04	SPAC1093.05	0.93	6.90E-04	SPAC1399.02	0.80	1.70E-04

SPBC18H10.07	0.93	6.75E-06	SPAPB24D3.03	0.80	1.18E-06
SPAC23H3.11c	0.93	2.82E-04	SPBC1773.08c	0.80	1.27E-05

	Δ*gcn5*			Δ*mst2*	
Gene	Log2 Change	pvalue	Gene	Log2 Change	pvalue
SPAC513.03	4.73	8.99E-03	SPBCPT2R1.08c	2.69	1.20E-02
SPBC1711.02	4.18	8.90E-03	SPBC359.06	2.29	2.33E-02
SPBCPT2R1.08c	3.89	7.73E-04	SPAC3G6.07	1.37	2.68E-02
SPAC1F8.01	3.87	8.30E-03	SPAC6B12.03c	1.36	1.52E-02
SPBPJ4664.03	3.40	3.66E-03	SPBC32F12.15	1.22	9.22E-03
SPBC359.06	3.20	2.79E-03	SPAC1565.04c	1.21	1.46E-03
SPAPB8E5.05	3.04	2.62E-03	SPBC27B12.02	1.21	2.89E-02
SPCC1739.08c	2.94	2.22E-02	SPAC14C4.08	1.19	6.86E-03
SPAC27D7.03c	2.57	5.66E-03	SPAC11E3.06	1.16	2.62E-02
SPAC11H11.04	2.45	3.38E-03	SPCC162.10	1.14	2.51E-02
SPCC794.01c	2.34	3.09E-02	SPCC1906.04	1.13	8.22E-03
SPAC31G5.09c	2.26	4.08E-03	SPAC4H3.03c	1.13	3.71E-02
SPCC188.12	2.05	4.71E-03	SPAC167.06c	1.09	9.36E-03
SPBC23G7.10c	1.80	4.08E-02	SPBC409.03	1.06	1.26E-02
SPCC1442.01	1.76	3.57E-03	SPBC3E7.02c	1.05	4.17E-02
SPAC11E3.06	1.72	2.01E-03	SPAC18G6.13	1.03	9.99E-03
SPAC56F8.15	1.71	2.55E-02	SPBC31F10.08	1.03	5.15E-03
SPCC162.10	1.65	2.36E-03	SPBC1773.12	1.03	2.04E-02
SPBC32C12.02	1.63	5.27E-03	SPBC405.05	1.01	8.87E-03
SPAC22F3.12c	1.58	8.83E-03	SPBC409.14c	0.96	5.74E-03
SPCC777.04	1.42	8.73E-03	SPAC3H1.03	0.96	4.27E-02
SPAC977.16c	1.36	3.70E-04	SPAC3H1.08c	0.95	1.02E-02
SPCC70.04c	1.31	2.14E-04	SPCP1E11.07c	0.94	3.68E-02
SPCC1906.04	1.29	3.25E-03	SPCC622.06c	0.92	1.64E-02
SPAP11E10.02c	1.26	3.91E-03	SPBC1105.16c	0.92	1.87E-02

SPAC23E2.03c	1.25	3.36E-02	SPCC1442.11c	0.91	7.26E-03
SPAC20H4.11c	1.19	3.84E-03	SPAC22F3.11c	0.90	3.14E-02
SPAPB1A10.14	1.16	1.25E-03	SPBC36B7.06c	0.90	6.07E-03
SPCC1020.01c	1.15	4.37E-03	SPBC2A9.12	0.89	4.95E-02
SPAC3G9.11c	1.07	9.81E-03	SPCC338.18	0.89	4.28E-02
SPCC338.18	1.02	2.22E-02	SPCC622.03c	0.89	1.44E-02
SPBC25B2.02c	1.02	1.38E-02	SPAC4G9.13c	0.87	3.34E-03
SPAC14C4.01c	0.98	2.93E-03	SPBC30D10.04	0.87	8.41E-03
SPACUNK4.10	0.95	1.37E-02	SPAC13C5.03	0.87	4.43E-04
SPAC1565.04c	0.92	1.07E-02	SPAC15F9.01c	0.86	2.80E-03
SPBC21C3.10c	0.92	2.80E-02	SPCC777.11	0.86	2.38E-02
SPAC1F8.05	0.88	2.57E-02	SPAC12B10.06c	0.85	1.76E-02
SPBC19C2.05	0.87	4.88E-02	SPAPB1A10.14	0.85	1.20E-02
SPAC26H5.09c	0.86	3.31E-02	SPBC887.16	0.84	2.97E-02
SPCC1529.01	0.84	3.97E-02	SPBC21B10.13c	0.84	1.55E-02
SPAC1952.04c	0.81	4.07E-02	SPCC830.04c	0.82	6.52E-03
SPAC13C5.03	0.81	9.05E-04	SPAC15A10.12c	0.81	1.99E-02
			SPAPB24D3.04c	0.80	1.38E-03

	Δgcn5 Δelp3			*Δgcn5 Δmst2*			*Δmst2 Δelp3*	
Gene	Log2 Change	pvalue	Gene	Log2 Change	pvalue	Gene	Log2 Change	pvalue
SPBCPT2R1.08c	4.68	3.86E-04	SPBCPT2R1.08c	6.70	1.71E-06	SPAC977.16c	4.40	3.91E-11
SPAC977.05c	4.56	1.72E-06	SPCC1795.06	6.36	9.50E-17	SPCC330.05c	4.25	1.65E-02
SPAC977.16c	3.64	4.94E-09	SPAC1F8.01	6.19	1.63E-04	SPBCPT2R1.08c	2.92	7.13E-03
SPBC359.02	3.05	4.16E-11	SPAC31G5.09c	4.76	1.85E-06	SPCC1393.10	2.88	2.52E-09
SPBPB8B6.04c	2.81	1.09E-06	SPBC359.06	4.72	7.39E-05	SPAC977.15	2.79	9.58E-03
SPBPB8B6.02c	2.78	2.18E-13	SPCC1739.08c	4.27	1.92E-03	SPBC354.08c	2.71	1.31E-08
SPAC2E1P3.02c	2.76	5.64E-09	SPBC4.01	4.20	1.88E-08	SPAC2E1P3.02c	2.32	1.45E-08
SPCC188.12	2.74	1.17E-03	SPAC22F3.12c	4.08	5.34E-07	SPBC1861.06c	2.19	1.62E-07
SPAC1952.04c	2.66	4.36E-06	SPCC794.01c	4.08	6.89E-04	SPBC359.02	2.16	1.83E-09

SPAC1006.04c	2.62	8.20E-09	SPAC27D7.03c	4.01	1.14E-04	SPCC737.04	2.15	3.53E-04
SPAC24C9.15c	2.58	4.26E-05	SPCC162.10	3.93	1.21E-07	SPAPB24D3.07c	2.07	4.94E-03
SPCC777.04	2.48	2.27E-04	SPCC188.12	3.84	1.08E-05	SPBC1778.04	2.03	2.28E-08
SPBC1778.04	2.46	6.92E-09	SPAC1F5.09c	3.80	2.81E-11	SPBC2G2.17c	2.02	8.49E-06
SPBPB8B6.03	2.35	1.43E-08	SPAC3G9.11c	3.74	8.98E-09	SPBC31F10.08	2.00	7.89E-06
SPAC750.04c	2.35	3.46E-07	SPBC56F2.06	3.68	2.97E-06	SPAC56F8.12	1.98	3.35E-08
SPBC31F10.08	2.27	6.61E-06	SPBC1198.14c	3.67	5.79E-13	SPAPB1A11.01	1.97	2.41E-07
SPAC750.07c	2.27	6.97E-05	SPAPB24D3.10c	3.59	1.78E-03	SPAC6C3.03c	1.92	1.73E-06
SPCC1393.10	2.20	6.91E-07	SPCC1442.01	3.56	2.49E-06	SPAC1142.05	1.87	4.14E-08
SPBC146.11c	2.20	3.50E-09	SPCC330.05c	3.53	4.12E-02	SPCC132.04c	1.86	9.18E-06
SPAC186.01	2.18	1.12E-06	SPCC548.07c	3.38	1.44E-02	SPCC830.04c	1.85	1.78E-06
SPAC977.07c	2.16	1.03E-07	SPBC32C12.02	3.19	7.81E-06	SPBC146.11c	1.84	9.60E-09
SPAC11H11.04	2.15	1.63E-02	SPAC11E3.06	3.11	4.16E-06	SPAC11H11.04	1.83	2.09E-02
SPBC354.08c	2.03	3.56E-06	SPCC737.04	2.99	8.94E-06	SPCC330.03c	1.81	1.20E-06
SPBC1348.01	2.02	5.55E-03	SPAC23E2.03c	2.99	3.26E-05	SPAC32A11.01	1.81	2.58E-04
SPBC32H8.11	2.00	7.97E-06	SPCC1906.04	2.90	5.26E-07	SPAC18B11.03c	1.79	1.44E-05
SPCC830.04c	1.91	4.97E-06	SPAC6B12.03c	2.86	2.44E-05	SPAC4G9.05	1.78	2.15E-06
SPAC4G9.05	1.89	4.35E-06	SPBC19C2.05	2.79	2.48E-06	SPAC10F6.15	1.78	6.86E-08
SPCC1020.01c	1.86	1.69E-04	SPAC20H4.11c	2.70	5.84E-07	SPAC977.17	1.76	1.58E-06
SPCC74.02c	1.86	6.50E-08	SPAC3F10.10c	2.65	5.28E-12	SPCPB1C11.02	1.71	2.25E-05
SPBC1861.06c	1.85	6.74E-06	SPAC1F8.08	2.65	6.91E-08	SPBC1861.01c	1.71	1.59E-03
SPCC1020.09	1.85	5.86E-04	SPBC21D10.06c	2.64	1.15E-12	SPAPB1A10.08	1.70	2.16E-08
SPAC57A7.05	1.79	2.17E-03	SPCC338.18	2.63	4.83E-06	SPBC21C3.02c	1.69	5.34E-08
SPAC4F10.08	1.76	5.76E-08	SPBC1683.08	2.63	6.85E-04	SPCC663.06c	1.67	2.19E-02
SPCC1223.02	1.74	1.11E-06	SPBPB2B2.12c	2.63	2.11E-02	SPCC417.04	1.66	1.68E-09
SPBC1347.03	1.70	1.87E-06	SPAC4H3.03c	2.57	7.41E-05	SPBC8E4.02c	1.66	6.10E-08
SPAC8E11.03c	1.70	1.17E-04	SPAC977.16c	2.56	1.74E-07	SPAC869.02c	1.66	2.21E-04
SPBC21C3.02c	1.65	3.77E-07	SPCC794.04c	2.42	8.62E-04	SPAC11D3.05	1.64	3.58E-04
SPBC18E5.10	1.64	7.52E-07	SPBC146.02	2.33	5.56E-08	SPBC8D2.19	1.64	1.62E-07
SPCC162.04c	1.64	2.53E-06	SPACUNK4.17	2.32	3.65E-05	SPAC186.06	1.62	5.18E-03
SPBC2G2.17c	1.62	3.31E-04	SPCC70.04c	2.32	1.90E-07	SPAC24C9.15c	1.62	1.40E-03

SPCC777.03c	1.61	1.49E-04	SPAC13F5.03c	2.24	2.84E-05	SPBC19C7.04c	1.60	9.14E-03
SPCC1919.14c	1.61	5.39E-06	SPAC13C5.03	2.23	2.19E-09	SPCC74.02c	1.57	1.47E-07
SPBC36.01c	1.60	5.09E-05	SPAC14C4.01c	2.19	4.47E-07	SPAC343.07	1.56	1.31E-06
SPBC8E4.02c	1.60	5.36E-07	SPCC777.04	2.19	2.67E-04	SPBC36.01c	1.53	2.38E-05
SPAC977.06	1.58	1.23E-08	SPBC1289.16c	2.18	1.83E-04	SPAC25H1.09	1.52	1.38E-06
SPAC5H10.04	1.58	1.55E-04	SPAC1F8.05	2.18	1.15E-05	SPAC6C3.05	1.49	2.55E-06
SPAC5H10.07	1.56	6.58E-06	SPBC725.10	2.12	6.57E-04	SPAC15A10.10	1.48	3.51E-07
SPBC23G7.11	1.54	3.44E-04	SPCC1393.12	2.09	1.29E-09	SPBC32H8.11	1.47	8.25E-05
SPCC417.04	1.54	3.12E-08	SPCC1223.12c	2.09	3.59E-07	SPBC9B6.03	1.46	2.51E-04
SPAC343.07	1.54	6.73E-06	SPAC31G5.07	2.07	6.02E-09	SPAC5H10.04	1.46	1.08E-04
SPAC56F8.14c	1.53	2.57E-04	SPCP31B10.06	2.06	1.01E-06	SPCC330.07c	1.44	2.45E-07
SPCC132.04c	1.52	2.99E-04	SPAC5H10.01	2.06	2.73E-02	SPCC584.02	1.44	2.33E-06
SPBC317.01	1.52	7.96E-05	SPBC16E9.16c	2.04	3.18E-08	SPAC4F10.08	1.44	2.05E-07
SPAC1F12.10c	1.52	2.89E-02	SPAC1565.04c	1.97	9.48E-06	SPACUNK4.09	1.43	6.27E-06
SPBC582.06c	1.52	2.40E-08	SPAC22F8.05	1.82	2.21E-03	SPAC57A7.05	1.43	5.01E-03
SPAC27D7.13c	1.50	7.42E-08	SPAC167.06c	1.80	1.42E-04	SPBC8E4.03	1.41	1.07E-05
SPBC27.03	1.50	5.24E-07	SPAC56F8.15	1.78	2.08E-02	SPAC4F10.09c	1.39	4.10E-06
SPAPB1A11.03	1.49	8.74E-07	SPAC29A4.12c	1.77	1.12E-04	SPAPB18E9.03c	1.39	5.16E-04
SPBC215.13	1.49	5.37E-08	SPCC569.03	1.74	1.64E-05	SPBC660.15	1.38	1.40E-05
SPCC1450.08c	1.48	1.35E-04	SPBC19C7.04c	1.69	6.22E-03	SPAC11D3.01c	1.37	5.96E-03
SPAC4F10.09c	1.47	8.06E-06	SPAC4H3.04c	1.66	4.45E-05	SPAC144.09c	1.37	2.09E-06
SPAC1A6.11	1.47	7.89E-07	SPAC1610.03c	1.66	1.23E-07	SPBC18E5.10	1.36	2.03E-06
SPBC3D6.11c	1.46	5.95E-07	SPBC24C6.06	1.65	2.64E-06	SPAC1952.15c	1.32	3.49E-06
SPAC31G5.10	1.46	4.44E-05	SPAC1952.04c	1.65	2.86E-04	SPAC1006.04c	1.32	2.26E-05
SPBPB2B2.18	1.44	2.34E-02	SPCC1450.08c	1.61	1.48E-05	SPBC6B1.03c	1.31	3.08E-06
SPBPB2B2.07c	1.43	4.35E-07	SPCC757.03c	1.60	2.25E-05	SPAC3H1.06c	1.30	2.41E-02
SPAC3H1.06c	1.42	2.71E-02	SPBC19C2.04c	1.60	2.07E-05	SPAC14C4.01c	1.30	2.38E-04
SPBC725.10	1.42	2.39E-02	SPBPB2B2.13	1.58	4.84E-02	SPCC1919.14c	1.29	2.07E-05
SPBC530.11c	1.41	1.76E-04	SPBC1711.11	1.56	4.60E-07	SPBC1685.13	1.29	4.03E-02
SPBC56F2.03	1.40	1.29E-06	SPBC13A2.04c	1.54	3.92E-03	SPCC757.11c	1.28	3.98E-05
SPAC25H1.09	1.40	1.70E-05	SPBC11C11.06c	1.54	7.57E-03	SPAC6G9.12	1.27	7.86E-07

SPBC1685.13	1.39	4.63E-02	SPAC30D11.02c	1.52	5.32E-04	SPBC1347.03	1.25	2.23E-05
SPBC660.15	1.39	4.73E-05	SPBC725.03	1.52	1.37E-03	SPBC21D10.08c	1.24	6.16E-03
SPAC1805.03c	1.37	3.64E-07	SPAC13G7.02c	1.51	1.89E-02	SPBC609.04	1.23	1.35E-02
SPBC14C8.05c	1.37	4.13E-07	SPCC285.07c	1.50	2.25E-05	SPCC1020.09	1.23	6.34E-03
SPBC29A10.02	1.36	3.82E-05	SPBC1347.03	1.49	2.50E-06	SPBC14C8.01c	1.22	4.12E-04
SPAC2C4.06c	1.36	1.29E-05	SPBC1685.06	1.48	3.30E-05	SPBP8B7.18c	1.22	3.70E-07
SPCC162.02c	1.35	1.72E-07	SPCC777.03c	1.47	1.17E-04	SPAPB18E9.04c	1.21	1.46E-04
SPBC1D7.05	1.34	2.29E-04	SPAC2F7.06c	1.47	1.41E-03	SPCC569.07	1.20	2.90E-04
SPBC6B1.03c	1.33	1.00E-05	SPBC1685.05	1.45	5.80E-07	SPAC22F8.11	1.20	2.99E-08
SPBC1348.09	1.33	5.45E-08	SPCC1223.02	1.45	2.97E-06	SPCC417.06c	1.20	4.80E-05
SPAC16E8.05c	1.33	1.32E-05	SPAC1751.01c	1.45	4.96E-03	SPBC1685.06	1.19	3.25E-04
SPBC21B10.12	1.32	5.47E-08	SPAC637.03	1.44	2.98E-03	SPBC3D6.11c	1.19	2.01E-06
SPCC1259.14c	1.32	2.23E-06	SPCC576.01c	1.42	1.24E-04	SPAC2E1P3.04	1.16	2.98E-05
SPBC1685.06	1.32	3.69E-04	SPCC794.02	1.42	3.45E-05	SPAC8C9.11	1.16	1.99E-04
SPBC18H10.09	1.32	3.29E-07	SPCC4G3.03	1.42	8.82E-06	SPCC18.10	1.16	7.43E-08
SPCC13B11.03c	1.32	7.25E-08	SPCC970.11c	1.41	7.07E-05	SPAC2C4.06c	1.16	2.31E-05
SPAC22F3.03c	1.31	4.83E-03	SPAC1A6.06c	1.41	1.42E-05	SPAC15F9.01c	1.16	1.96E-04
SPBC8D2.19	1.29	1.71E-05	SPAC32A11.01	1.40	2.47E-03	SPAC926.08c	1.15	3.02E-03
SPCC569.04	1.28	9.07E-08	SPCC1739.15	1.40	1.46E-06	SPAC4H3.11c	1.15	2.56E-05
SPAC26A3.03c	1.28	3.43E-08	SPAC4G9.07	1.40	3.48E-05	SPBC13E7.02	1.15	3.21E-06
SPAC9.10	1.28	1.64E-05	SPCC1183.10	1.39	1.37E-08	SPCC162.02c	1.13	4.56E-07
SPAC22F3.02	1.28	1.00E-06	SPAC29A4.17c	1.39	4.24E-04	SPAPB24D3.02c	1.11	9.51E-08
SPBC1289.16c	1.27	2.50E-02	SPAP11E10.02c	1.38	1.95E-03	SPCC553.01c	1.09	2.44E-09
SPAC977.17	1.26	2.89E-04	SPCP20C8.02c	1.38	1.34E-05	SPBC3D6.03c	1.09	2.95E-04
SPBC36.02c	1.26	2.55E-02	SPAC4D7.02c	1.38	9.19E-05	SPCC962.02c	1.06	4.54E-05
SPBC1685.14c	1.25	5.32E-04	SPBC18H10.05	1.37	7.18E-06	SPBC56F2.03	1.06	1.05E-05
SPAC1952.15c	1.25	2.78E-05	SPCC1020.01c	1.37	1.08E-03	SPCC1672.03c	1.06	2.11E-04
SPBC13A2.04c	1.25	2.77E-02	SPBP19A11.07c	1.37	5.09E-08	SPCC285.09c	1.05	3.17E-04
SPCC584.16c	1.25	1.62E-02	SPACUNK4.10	1.35	1.16E-03	SPBC215.13	1.05	1.47E-06
SPBC18H10.07	1.24	6.45E-07	SPAPB15E9.02c	1.34	2.13E-05	SPAC1250.01	1.05	1.73E-05
SPCC4B3.10c	1.23	1.41E-03	SPBC1347.01c	1.33	2.83E-04	SPAC18G6.09c	1.04	1.62E-02

SPAC1006.03c	1.23	1.70E-06	SPAP7G5.03	1.33	1.25E-07	SPCC2H8.02	1.04	4.18E-06
SPBC19G7.13	1.22	3.39E-06	SPCC1020.05	1.33	4.17E-05	SPCC757.02c	1.04	1.52E-04
SPAC11H11.03c	1.22	7.70E-05	SPBC8E4.05c	1.32	4.73E-06	SPAC3C7.04	1.03	3.40E-07
SPAC19G12.01c	1.21	1.23E-08	SPCC290.04	1.32	5.40E-04	SPAC637.11	1.03	1.51E-05
SPAC26H5.06	1.21	9.36E-07	SPAPB18E9.04c	1.32	5.58E-05	SPBC947.13	1.02	8.86E-05
SPCC285.09c	1.21	2.43E-04	SPCPB1C11.02	1.31	4.02E-04	SPAC6C3.02c	1.02	2.14E-03
SPAC29B12.12	1.20	5.02E-04	SPCC74.09	1.31	1.88E-02	SPCC70.08c	1.02	1.98E-03
SPAC10F6.15	1.20	5.11E-05	SPMIT.06	1.31	1.09E-02	SPAC17A5.13	1.01	5.36E-05
SPAC1F8.04c	1.20	3.59E-02	SPBC21D10.08c	1.31	4.37E-03	SPBC3H7.06c	1.00	4.55E-03
SPAC6C3.07	1.20	1.91E-05	SPAC1006.04c	1.30	2.55E-05	SPAC9.10	1.00	7.44E-05
SPAC6G9.01c	1.19	1.32E-03	SPAC27D7.11c	1.29	3.52E-03	SPBC19C2.11c	1.00	1.86E-04
SPCC962.02c	1.18	4.84E-05	SPAC16E8.03	1.29	5.33E-05	SPCC1223.02	1.00	2.21E-04
SPCC576.17c	1.18	5.14E-05	SPAPB1A10.14	1.28	5.07E-04	SPBC24C6.02	1.00	8.98E-04
SPACUNK4.09	1.17	2.26E-04	SPAC688.03c	1.28	1.65E-05	SPCC1020.05	1.00	7.73E-04
SPAPB8E5.10	1.17	2.57E-04	SPAC8E11.03c	1.28	6.46E-04	SPAP7G5.03	0.99	6.36E-06
SPBC9B6.03	1.16	4.65E-03	SPAC19B12.08	1.27	1.47E-03	SPBC17D1.01	0.99	5.20E-05
SPAC56F8.13	1.16	1.37E-06	SPBC365.12c	1.25	2.02E-03	SPBPB8B6.03	0.99	2.31E-04
SPAC32A11.01	1.16	1.83E-02	SPCC70.02c	1.25	2.42E-05	SPBC1683.01	0.99	2.14E-04
SPAC926.08c	1.15	6.76E-03	SPBC14C8.05c	1.25	3.12E-07	SPAC22F3.02	0.99	6.54E-06
SPCC330.07c	1.15	2.16E-05	SPAC1F7.05	1.25	1.60E-03	SPAC1805.03c	0.99	6.68E-06
SPBC21D10.08c	1.14	2.01E-02	SPBC36.02c	1.25	1.46E-02	SPBP8B7.04	0.99	7.41E-05
SPAC11D3.06	1.14	2.30E-07	SPCC126.07c	1.24	2.16E-08	SPAC26H5.06	0.98	3.34E-06
SPBC18H10.21c	1.13	2.83E-05	SPAC1565.03	1.24	4.38E-04	SPCC1450.07c	0.98	2.82E-04
SPAC1A6.08c	1.13	1.51E-02	SPCPB16A4.06c	1.23	6.25E-04	SPBC28E12.02	0.98	1.64E-04
SPACUNK4.10	1.13	9.83E-03	SPMIT.02	1.23	6.56E-03	SPCC1827.01c	0.98	6.16E-03
SPCC188.09c	1.12	4.54E-07	SPBC660.07	1.23	5.63E-04	SPAC922.07c	0.98	1.24E-03
SPBC14C8.01c	1.11	2.58E-03	SPAC15F9.01c	1.22	1.13E-04	SPBP35G2.13c	0.97	1.89E-03
SPBC1652.01	1.11	2.03E-03	SPCC1393.07c	1.21	3.70E-06	SPAC19G12.01c	0.97	7.03E-08
SPAC57A7.06	1.10	1.28E-03	SPAC15A10.03c	1.21	3.37E-05	SPCC663.14c	0.96	3.78E-05
SPBC3D6.03c	1.10	7.60E-04	SPAC26H5.09c	1.21	4.35E-03	SPBC1685.14c	0.96	1.95E-03
SPBC17D1.07c	1.10	2.25E-03	SPAC2C4.17c	1.21	1.81E-05	SPCC11E10.01	0.96	2.68E-05

SPAC23D3.01	1.10	2.79E-06	SPBC1271.01c	1.21	1.05E-07	SPAC26H5.08c	0.96	8.91E-04
SPBC685.03	1.10	9.65E-07	SPBC2G2.17c	1.21	1.71E-03	SPCC790.02	0.96	3.31E-07
SPBCPT2R1.02	1.10	1.32E-03	SPAC17H9.19c	1.21	4.66E-03	SPBC3B8.09	0.96	2.52E-03
SPAC22F8.09	1.10	2.55E-02	SPBC1685.14c	1.20	2.63E-04	SPBC582.06c	0.96	3.28E-06
SPCC1906.04	1.09	1.98E-02	SPBC1347.11	1.20	4.08E-03	SPBC16A3.17c	0.96	2.32E-03
SPAC212.08c	1.09	9.05E-07	SPAC11D3.14c	1.20	2.97E-02	SPBC317.01	0.96	2.23E-03
SPBC23E6.09	1.09	7.17E-03	SPAC32A11.02c	1.19	1.75E-04	SPAC9E9.12c	0.95	4.72E-06
SPAC637.11	1.08	3.21E-05	SPBC119.07	1.19	1.53E-04	SPAC29A4.19c	0.95	5.06E-08
SPAC31A2.12	1.08	1.40E-05	SPBC15D4.02	1.19	1.21E-03	SPAC57A10.04	0.95	1.93E-05
SPCC18.10	1.08	1.03E-06	SPBC354.12	1.18	7.69E-05	SPBC16E9.10c	0.95	5.32E-04
SPBC216.02	1.07	1.43E-04	SPAC4A8.04	1.18	1.01E-03	SPBC359.01	0.95	8.06E-05
SPAPB1E7.01c	1.07	5.66E-04	SPAC1002.12c	1.18	5.46E-05	SPBC119.17	0.94	2.96E-04
SPBC16E9.10c	1.07	4.80E-04	SPAC4F10.16c	1.18	2.55E-05	SPBC18H10.09	0.94	6.33E-06
SPAC6C3.05	1.07	3.87E-04	SPAC25G10.04c	1.18	1.96E-04	SPAC328.09	0.94	3.27E-03
SPAC4H3.11c	1.07	1.91E-04	SPBC14C8.11c	1.15	3.09E-04	SPAC1250.02	0.94	4.92E-04
SPCC31H12.02c	1.07	2.16E-05	SPAC29B12.12	1.15	2.67E-04	SPCC18.13	0.93	1.50E-03
SPCC11E10.09c	1.06	6.81E-06	SPAC9.10	1.14	1.67E-05	SPAC4G9.10	0.93	2.85E-05
SPCC290.02	1.06	6.79E-04	SPAC5H10.07	1.13	7.77E-05	SPCC965.11c	0.93	4.57E-03
SPBC17D1.01	1.06	8.69E-05	SPAC2E1P3.02c	1.13	1.67E-04	SPAC21E11.04	0.93	3.43E-04
SPBC28E12.02	1.06	2.41E-04	SPAC17A5.04c	1.13	1.67E-08	SPAC1527.03	0.93	2.37E-04
SPAC57A10.04	1.05	2.14E-05	SPAC1805.15c	1.12	4.53E-05	SPAC56E4.06c	0.92	3.24E-06
SPAC1250.01	1.05	5.80E-05	SPAP8A3.04c	1.12	3.58E-02	SPAC14C4.02c	0.92	2.57E-04
SPAC1527.03	1.05	2.09E-04	SPMIT.05	1.11	4.20E-02	SPAC57A7.06	0.91	2.39E-03
SPAC1F8.06	1.05	6.62E-03	SPAC1F7.06	1.11	5.51E-07	SPAC29A4.10	0.91	1.13E-03
SPCC330.09	1.05	4.29E-05	SPBC32F12.09	1.09	3.21E-05	SPBC27.03	0.91	6.32E-05
SPBC29A3.14c	1.05	3.35E-07	SPAC16E8.02	1.09	2.42E-04	SPAC323.04	0.91	2.86E-04
SPAC1B3.15c	1.05	4.06E-04	SPCC569.02c	1.09	2.26E-05	SPAC27D7.13c	0.91	1.39E-05
SPCC1827.01c	1.05	8.20E-03	SPBC14C8.07c	1.08	1.46E-03	SPAC8C9.10c	0.91	7.80E-03
SPCC18.05c	1.05	2.63E-05	SPCC306.08c	1.08	9.75E-04	SPBC19G7.06	0.91	5.88E-06
SPAC27D7.05c	1.05	2.84E-03	SPBC36B7.06c	1.08	1.61E-03	SPAC31A2.12	0.91	3.11E-05
SPBC24C6.02	1.05	1.52E-03	SPBC21C3.10c	1.07	1.17E-02	SPBC19C7.03	0.91	1.21E-03

SPAC22H10.10	1.05	1.91E-05	SPBC947.15c	1.07	1.15E-03	SPCC13B11.04c	0.90	7.51E-04
SPBC947.13	1.05	2.26E-04	SPBC14C8.01c	1.07	1.43E-03	SPAC23D3.01	0.90	8.16E-06
SPBC6B1.05c	1.04	1.08E-05	SPAC22F3.03c	1.06	9.08E-03	SPBC1604.01	0.90	1.04E-05
SPBC119.14	1.04	2.29E-05	SPBC1D7.05	1.06	7.39E-04	SPBC26H8.08c	0.90	4.23E-03
SPBC21C3.10c	1.04	2.55E-02	SPAC1006.01	1.05	1.71E-05	SPAC922.03	0.90	4.12E-06
SPAC12D12.09	1.04	1.44E-05	SPBC24C6.09c	1.05	5.72E-04	SPAC6G9.01c	0.90	5.00E-03
SPCC24B10.14c	1.04	7.59E-05	SPBC1A4.01	1.05	4.79E-04	SPCC576.17c	0.89	3.00E-04
SPCC126.02c	1.04	4.35E-05	SPAC26F1.04c	1.05	1.33E-04	SPCC1322.03	0.89	4.63E-03
SPAC8F11.05c	1.03	7.83E-04	SPAC17A2.07c	1.05	2.17E-05	SPCC1281.07c	0.89	1.32E-02
SPAC25B8.06c	1.03	9.70E-05	SPAC26H5.04	1.04	3.82E-06	SPCC613.12c	0.89	1.30E-04
SPCC1020.05	1.03	1.54E-03	SPCC191.01	1.04	3.16E-03	SPAC6B12.08	0.89	2.84E-04
SPBC1703.04	1.03	3.15E-08	SPBP23A10.04	1.04	7.76E-07	SPBC651.01c	0.89	9.17E-04
SPAC3A11.07	1.02	1.42E-02	SPAC513.02	1.03	3.47E-03	SPAC4A8.07c	0.89	2.87E-04
SPAC1F8.02c	1.02	4.31E-03	SPCPJ732.02c	1.03	2.86E-05	SPAC7D4.08	0.89	6.16E-03
SPCC584.02	1.02	4.23E-04	SPAC3A11.07	1.03	6.82E-03	SPBC19F5.02c	0.89	6.72E-04
SPBC651.01c	1.02	7.35E-04	SPAC23C11.06c	1.03	9.35E-03	SPBC354.09c	0.88	9.76E-07
SPBC3F6.04c	1.02	2.21E-03	SPBC27B12.01c	1.02	1.84E-03	SPBC1685.12c	0.88	2.53E-02
SPBC29A10.14	1.02	1.71E-04	SPBC3D6.03c	1.01	5.93E-04	SPCC290.02	0.88	1.31E-03
SPBC16D10.07c	1.01	7.85E-08	SPCC330.04c	1.01	1.22E-05	SPAC1093.05	0.88	1.13E-03
SPBC3D6.10	1.01	9.35E-04	SPAC26F1.14c	1.01	9.34E-03	SPAC1006.03c	0.88	2.78E-05
SPAC22G7.11c	1.01	1.86E-03	SPBC336.12c	1.01	8.55E-07	SPAC2E1P5.05	0.87	2.06E-03
SPCC297.06c	1.01	6.67E-03	SPAC1805.09c	1.01	5.47E-04	SPBC530.07c	0.87	1.81E-05
SPAC30D11.07	1.00	7.39E-05	SPAC4G8.04	1.00	1.11E-06	SPBC1711.07	0.87	6.41E-03
SPAC27D7.11c	1.00	3.16E-02	SPCC338.08	1.00	1.28E-04	SPCC737.03c	0.87	1.40E-04
SPAC890.05	1.00	3.10E-03	SPCC285.16c	1.00	5.57E-07	SPBC1703.08c	0.87	2.74E-02
SPBC336.05c	1.00	2.80E-02	SPBC19C2.09	1.00	2.43E-02	SPAPJ760.03c	0.86	6.24E-03
SPBC3H7.06c	0.99	1.00E-02	SPBC800.14c	0.99	8.10E-04	SPCC417.03	0.86	1.81E-05
SPAC26A3.12c	0.99	4.17E-05	SPCC162.04c	0.99	2.34E-04	SPBP8B7.30c	0.86	1.80E-05
SPBC25H2.08c	0.99	8.82E-04	SPAC3A11.06	0.99	8.27E-04	SPAC24H6.13	0.85	3.80E-03
SPCC23B6.03c	0.99	1.85E-04	SPAC22G7.11c	0.99	8.41E-04	SPBC947.04	0.85	4.95E-02
SPBC13E7.02	0.98	7.76E-05	SPBC25H2.08c	0.99	3.31E-04	SPAC3G6.04	0.85	3.25E-03

SPAC17A5.04c	0.98	6.53E-07	SPAC3A11.10c	0.99	2.36E-03	SPCC1020.13c	0.85	3.53E-04
SPCC1450.09c	0.98	6.63E-05	SPBC19C7.03	0.98	5.76E-04	SPBC1271.07c	0.85	2.62E-04
SPBC1718.02	0.97	1.14E-03	SPAC31G5.19	0.98	2.16E-04	SPCC1259.14c	0.85	1.16E-04
SPAC17A5.11	0.96	9.07E-04	SPAC13F5.07c	0.98	1.21E-02	SPBC902.06	0.85	3.79E-04
SPCC16C4.05	0.96	3.24E-03	SPAC1399.01c	0.98	1.43E-04	SPBC6B1.05c	0.85	3.50E-05
SPAC4H3.05	0.95	4.22E-06	SPBC651.05c	0.98	1.87E-04	SPAC22F8.09	0.85	5.01E-02
SPCC613.12c	0.95	2.03E-04	SPBC18A7.01	0.97	5.17E-04	SPBC1198.12	0.84	5.63E-04
SPAC16.04	0.95	5.06E-04	SPAC24C9.07c	0.97	2.49E-04	SPCC1494.06c	0.84	2.97E-03
SPBC119.17	0.95	7.86E-04	SPBC1683.01	0.97	2.71E-04	SPAC29B12.12	0.84	3.97E-03
SPCC1494.06c	0.95	2.86E-03	SPBC25H2.09	0.97	2.22E-04	SPAC17A2.12	0.84	2.00E-03
SPBC337.11	0.95	2.04E-06	SPCPJ732.03	0.97	6.75E-04	SPAC140.02	0.84	1.10E-02
SPAC9E9.12c	0.95	1.98E-05	SPAC1952.15c	0.96	1.46E-04	SPCC757.09c	0.83	1.22E-03
SPBC11C11.04c	0.95	1.99E-05	SPAC3C7.05c	0.96	1.99E-04	SPAC19B12.01	0.83	5.83E-03
SPCC1393.05	0.95	2.55E-04	SPBC1773.08c	0.96	1.16E-06	SPBC19C2.01	0.83	5.22E-04
SPBC19C7.03	0.95	2.12E-03	SPBC23G7.06c	0.95	1.53E-04	SPAC26A3.03c	0.83	3.14E-06
SPBC26H8.08c	0.94	6.64E-03	SPBC1604.18c	0.94	4.20E-04	SPAC1D4.11c	0.83	1.19E-04
SPBP19A11.07c	0.94	3.19E-05	SPBC2F12.05c	0.94	5.74E-05	SPCC550.10	0.83	4.58E-05
SPAC24H6.06	0.94	5.61E-05	SPAC513.05	0.94	1.15E-03	SPBC18H10.07	0.83	2.78E-05
SPBC1773.08c	0.94	6.57E-06	SPCC584.15c	0.94	2.45E-04	SPAC16.04	0.83	6.80E-04
SPCC622.19	0.94	2.77E-04	SPCC1322.08	0.94	1.06E-02	SPAC11E3.09	0.83	4.23E-04
SPAC4D7.10c	0.94	6.09E-04	SPAC6G9.16c	0.94	2.13E-04	SPAC30D11.03	0.82	2.24E-03
SPAP8A3.11c	0.94	1.17E-03	SPAC16A10.01	0.94	4.99E-04	SPBC30B4.01c	0.82	4.11E-02
SPCC895.06	0.93	3.39E-04	SPCC1442.05c	0.93	8.43E-04	SPBC1D7.05	0.82	5.43E-03
SPBC106.08c	0.93	5.68E-04	SPAC19A8.05c	0.93	1.83E-04	SPAPB1E7.01c	0.82	2.10E-03
SPAP7G5.03	0.93	5.23E-05	SPCC1183.11	0.93	1.45E-03	SPAC167.08	0.82	4.99E-03
SPCC576.19c	0.93	1.54E-03	SPAC227.15	0.93	4.12E-05	SPAC22E12.09c	0.82	1.57E-03
SPCP20C8.02c	0.93	3.76E-02	SPBC1105.10	0.93	1.11E-05	SPAC17A5.04c	0.82	1.52E-06
SPBP8B7.27	0.93	1.77E-05	SPAC1093.06c	0.93	1.90E-04	SPBPB8B6.05c	0.82	1.20E-02
SPBC359.04c	0.92	6.29E-03	SPBC660.06	0.92	2.82E-03	SPBC18H10.21c	0.81	2.95E-04
SPCC550.12	0.92	4.92E-03	SPAPB2B4.06	0.92	6.13E-05	SPBC16G5.16	0.81	3.19E-05
SPAC328.08c	0.92	5.57E-03	SPCC737.09c	0.92	6.96E-05	SPAC26F1.12c	0.81	1.48E-02

SPAC24H6.13	0.92	5.05E-03	SPBP8B7.24c	0.92	1.65E-03	SPBP23A10.12	0.81	1.24E-03
SPBC19G7.06	0.92	2.13E-05	SPAC16C9.01c	0.92	4.20E-03	SPAC869.11	0.81	9.45E-04
SPBC36.07	0.92	1.15E-04	SPCC24B10.14c	0.92	8.40E-05	SPAPB1A10.06c	0.81	1.39E-04
SPAC29A4.13	0.92	2.51E-04	SPAC22E12.03c	0.92	6.85E-04	SPAC328.08c	0.81	6.19E-03
SPAC18B11.09c	0.91	9.67E-04	SPBC902.05c	0.92	3.85E-04	SPBC14C8.08c	0.81	3.66E-04
SPAC1527.01	0.91	1.68E-04	SPAC15A10.05c	0.91	1.25E-02	SPAC8E11.03c	0.81	1.80E-02
SPCC1919.07	0.91	1.22E-02	SPAC824.07	0.91	1.48E-05	SPAC821.04c	0.81	2.29E-02
SPBC1198.12	0.90	8.31E-04	SPCC4B3.01	0.90	6.97E-04	SPBC2G2.01c	0.80	1.63E-03
SPAC823.04	0.90	4.09E-02	SPBC3H7.08c	0.90	3.06E-04	SPCC895.06	0.80	4.86E-04
SPCC970.01	0.90	2.98E-05	SPBC23E6.09	0.90	1.15E-02	SPAC11D3.06	0.80	5.61E-06
SPCC70.09c	0.90	8.42E-04	SPAC11H11.01	0.90	1.54E-03	SPAC20H4.04	0.80	1.11E-07
SPAC167.08	0.90	5.84E-03	SPAC17G6.12	0.90	2.27E-04	SPCC645.06c	0.80	3.91E-04
SPBC31F10.11c	0.90	5.38E-05	SPAPB2B4.03	0.90	6.99E-03	SPBC1773.13	0.80	6.50E-05
SPBC29A10.09c	0.89	5.25E-04	SPBC839.06	0.89	6.58E-05	SPCC63.06	0.80	5.51E-05
SPCC1223.11	0.89	9.64E-03	SPBC32F12.03c	0.89	9.60E-04	SPCC1739.03	0.80	4.76E-06
SPAC11E3.09	0.89	5.72E-04	SPAC959.05c	0.89	2.20E-05			
SPAC1250.02	0.89	1.99E-03	SPAC23C4.13	0.89	1.19E-02			
SPCC162.03	0.89	2.71E-04	SPBC19F5.01c	0.88	1.39E-03			
SPAC29A4.19c	0.89	6.59E-07	SPAC31G5.10	0.88	1.91E-03			
SPAPB1A10.06c	0.89	1.72E-04	SPAC22A12.02c	0.88	1.08E-04			
SPAC922.07c	0.89	6.15E-03	SPCC1281.04	0.88	1.23E-03			
SPAC23H3.11c	0.88	1.23E-03	SPCC736.15	0.88	1.19E-02			
SPAC2E1P5.05	0.88	4.49E-03	SPAC20G4.02c	0.87	9.70E-03			
SPCC4B3.08	0.88	6.47E-04	SPCC16A11.15c	0.87	6.26E-04			
SPCC24B10.22	0.88	7.65E-04	SPCP1E11.03	0.87	7.00E-07			
SPCC126.07c	0.88	1.18E-05	SPCC23B6.03c	0.87	2.24E-04			
SPCC330.03c	0.88	6.23E-03	SPBC106.08c	0.87	3.82E-04			
SPAC16E8.06c	0.88	8.30E-03	SPBC27B12.04c	0.86	2.96E-06			
SPBP8B7.04	0.88	7.41E-04	SPAC17G6.04c	0.86	6.89E-04			
SPBC29A10.10c	0.87	2.59E-03	SPBC1271.05c	0.86	5.59E-03			
SPBC713.06	0.87	2.32E-03	SPAC19B12.10	0.86	3.43E-04			

SPCC645.13	0.87	8.86E-05	SPBC16G5.07c	0.86	8.04E-06
SPCC11E10.01	0.87	2.67E-04	SPBC16A3.02c	0.85	6.22E-04
SPBP35G2.03c	0.87	2.63E-03	SPCC338.12	0.85	8.62E-03
SPAC3F10.10c	0.87	1.95E-04	SPBC1718.01	0.85	1.88E-06
SPAC6F6.04c	0.87	7.95E-06	SPAC630.05	0.85	1.75E-03
SPAC19A8.07c	0.86	1.19E-02	SPAP14E8.02	0.84	1.91E-02
SPAC3G6.04	0.86	6.58E-03	SPCC737.03c	0.84	1.86E-04
SPCC594.04c	0.86	5.09E-04	SPBC428.07	0.84	1.37E-04
SPBC577.09	0.86	3.21E-05	SPAC1952.16	0.84	1.14E-04
SPBC21H7.04	0.86	2.32E-03	SPBC776.15c	0.84	6.71E-03
SPAPB24D3.02c	0.85	1.39E-05	SPAC458.04c	0.84	8.96E-03
SPBC1198.04c	0.85	7.63E-06	SPAC1296.01c	0.84	3.61E-06
SPAC1805.15c	0.85	1.95E-03	SPCC1682.11c	0.84	4.61E-04
SPCC70.08c	0.85	1.41E-02	SPCC162.02c	0.84	2.12E-05
SPAC16C9.01c	0.85	1.43E-02	SPAC4F10.02	0.84	2.72E-05
SPBC28F2.12	0.85	2.46E-03	SPAC4F10.07c	0.84	3.46E-06
SPCC1919.11	0.84	7.62E-06	SPBC15D4.01c	0.83	7.54E-03
SPCC11E10.03	0.84	2.53E-04	SPBC216.03	0.83	5.43E-04
SPAC222.05c	0.84	4.44E-06	SPBC6B1.05c	0.83	4.28E-05
SPAC14C4.01c	0.84	1.70E-02	SPBC83.05	0.83	6.11E-04
SPCC553.01c	0.84	6.21E-07	SPBC16C6.02c	0.83	2.60E-05
SPBC582.10c	0.84	3.30E-03	SPBC21C3.11	0.83	1.69E-03
SPCC417.11c	0.83	8.90E-03	SPBC1718.07c	0.83	1.26E-07
SPAC1952.07	0.83	8.84E-07	SPAC20G4.05c	0.82	1.01E-04
SPBC17D1.02	0.83	2.69E-03	SPAC22F8.04	0.81	1.50E-03
SPCC663.14c	0.83	5.39E-04	SPBC1718.06	0.81	2.95E-06
SPAC19G12.07c	0.83	6.46E-05	SPAC607.08c	0.81	2.43E-03
SPCC18.13	0.83	8.00E-03	SPBC1E8.05	0.80	8.54E-03
SPAC977.03	0.83	2.37E-03	SPAC823.16c	0.80	1.05E-04
SPAC2F7.17	0.83	1.82E-03			
SPAC56E4.06c	0.83	4.76E-05			

SPBC23G7.06c	0.83	1.55E-03
SPCC4E9.01c	0.83	2.64E-04
SPBC1271.07c	0.82	1.01E-03
SPBC8D2.05c	0.82	9.74E-05
SPAC22F8.11	0.82	2.36E-05
SPCC364.04c	0.81	5.08E-05
SPCC1322.03	0.81	1.67E-02
SPBC651.05c	0.81	2.59E-03
SPBC30B4.02c	0.81	7.95E-03
SPCC1442.04c	0.81	3.50E-04
SPCC63.06	0.81	1.57E-04
SPAC6G9.12	0.81	5.39E-04
SPAC1D4.11c	0.81	4.84E-04
SPBC21.03c	0.80	1.04E-02
SPAC20H4.04	0.80	5.11E-07
SPAC25B8.05	0.80	3.42E-04
SPCP31B10.05	0.80	4.38E-05
SPAC16E8.14c	0.80	3.88E-04
SPAC23C11.03	0.80	7.87E-03
SPAC17G6.12	0.80	1.89E-03
SPBC1685.04	0.80	1.10E-03
SPCC553.08c	0.80	1.08E-03
SPAC3A11.05c	0.80	1.50E-03

Δ*gcn5* Δ*elp3* Δ*mst2*

Gene	Log2 Change	pvalue	Gene	Log2 Change	pvalue	Gene	Log2 Change	pvalue
SPAC1F8.03c	7.18	1.76E-07	SPCC417.04	1.47	1.65E-06	SPAC29A4.19c	1.03	2.24E-06
SPBCPT2R1.08c	6.37	1.88E-04	SPBC3H7.06c	1.47	3.50E-03	SPBC146.05c	1.02	4.38E-03
SPAC1F8.02c	6.36	4.47E-12	SPAC22F3.02	1.46	3.66E-06	SPAC1805.15c	1.02	2.97E-03
SPAC513.03	6.13	1.53E-02	SPBC660.07	1.46	2.43E-03	SPCC962.02c	1.02	1.94E-03

SPBC1711.02	5.33	1.64E-02	SPAC2F7.06c	1.45	1.73E-02	SPAC637.11	1.02	6.69E-04
SPAC3G9.11c	4.62	6.82E-08	SPBPB2B2.02	1.44	4.01E-02	SPBC23G7.06c	1.02	1.91E-03
SPCC1795.06	4.47	1.54E-11	SPAC1F7.06	1.44	1.71E-06	SPCC576.04	1.02	3.56E-03
SPBC1683.09c	4.31	2.61E-04	SPBC1685.06	1.44	1.42E-03	SPCC1739.03	1.02	1.70E-05
SPAC1F8.01	4.18	3.58E-02	SPCC1020.01c	1.43	1.01E-02	SPCC1442.04c	1.01	3.83E-04
SPBC23G7.10c	4.12	2.21E-03	SPAC8C9.03	1.43	3.28E-03	SPBC609.01	1.01	4.17E-06
SPBC839.06	3.64	1.71E-11	SPBC21B10.12	1.43	5.28E-07	SPBC660.15	1.01	6.78E-03
SPCC1739.08c	3.56	4.61E-02	SPAC5H10.04	1.43	3.12E-03	SPAC16C9.01c	1.00	2.11E-02
SPCC188.12	3.56	9.35E-04	SPAC4G9.05	1.42	1.17E-03	SPBC1271.01c	1.00	9.67E-05
SPCC737.04	3.55	7.02E-05	SPBC24C6.09c	1.42	8.69E-04	SPAC57A7.08	1.00	3.98E-03
SPBC56F2.06	3.51	2.76E-04	SPCC569.04	1.42	6.19E-07	SPAC977.06	1.00	1.19E-04
SPAC977.16c	3.41	4.14E-07	SPBC13A2.04c	1.41	4.62E-02	SPAC1006.01	0.99	1.13E-03
SPBC947.05c	3.29	1.29E-08	SPCC830.07c	1.40	6.04E-03	SPCC1393.07c	0.99	1.37E-03
SPAC1F7.08	3.06	1.45E-03	SPAC23G3.03	1.39	1.54E-03	SPAC6C3.02c	0.98	2.53E-02
SPBC359.06	2.98	3.49E-02	SPBC947.13	1.39	1.38E-04	SPAC6G10.03c	0.98	7.71E-03
SPAPB8E5.05	2.89	3.07E-02	SPCC576.17c	1.39	1.15E-04	SPCC1235.01	0.98	6.12E-03
SPAC27D7.03c	2.82	2.54E-02	SPAC25H1.03	1.38	9.10E-05	SPBC13E7.02	0.98	8.26E-04
SPAC31G5.09c	2.76	1.09E-02	SPBC1347.11	1.38	1.54E-02	SPBC25H2.08c	0.98	6.14E-03
SPBC19C7.04c	2.68	2.75E-03	SPCC24B10.14c	1.38	4.26E-05	SPAC222.15	0.98	8.65E-04
SPAC1F8.08	2.67	6.91E-06	SPBC725.03	1.38	2.54E-02	SPCC11E10.09c	0.98	2.57E-04
SPCC1442.01	2.67	2.09E-03	SPCC126.07c	1.38	7.12E-07	SPBC119.14	0.97	5.68E-04
SPBC1347.03	2.67	9.57E-08	SPAC13D6.01	1.38	2.81E-04	SPCC548.05c	0.97	4.97E-04
SPCC1906.04	2.58	1.04E-05	SPBC106.02c	1.37	3.46E-02	SPAPB8E5.10	0.97	7.90E-03
SPAC22H10.13	2.53	2.35E-03	SPBC4F6.17c	1.36	1.43E-02	SPAC24H6.13	0.97	1.57E-02
SPAC1952.04c	2.53	1.24E-04	SPBC215.13	1.36	4.54E-06	SPCC553.07c	0.97	1.15E-03
SPAC922.03	2.52	1.72E-10	SPAC15A10.10	1.36	7.97E-05	SPAC3A11.05c	0.97	2.10E-03
SPBC2G2.17c	2.42	5.92E-05	SPCC285.07c	1.36	1.95E-03	SPAC24H6.06	0.97	4.79E-04
SPAC13G7.02c	2.41	9.23E-03	SPAC4D7.02c	1.36	2.65E-03	SPAC328.02	0.96	2.66E-05
SPBC1685.13	2.39	9.44E-03	SPCC330.03c	1.35	1.43E-03	SPAC2C4.06c	0.96	3.74E-03
SPAC11H11.04	2.39	3.15E-02	SPBC1198.14c	1.34	1.86E-04	SPBC1861.06c	0.96	1.98E-02
SPAC6B12.03c	2.37	4.00E-03	SPAC29B12.12	1.33	1.61E-03	SPBC1D7.05	0.95	1.85E-02

SPAC11E3.06	2.34	2.81E-03	SPCC1259.14c	1.32	4.21E-05	SPBC354.12	0.95	9.50E-03
SPAC8E11.03c	2.33	4.79E-05	SPBC36B7.06c	1.31	4.97E-03	SPAC18B11.09c	0.95	4.68E-03
SPAC27D7.09c	2.32	6.89E-03	SPBC32H8.06	1.30	4.54E-04	SPAPB1E7.01c	0.94	9.35E-03
SPAC4H3.03c	2.32	4.33E-03	SPCC777.03c	1.29	7.02E-03	SPAC17A2.07c	0.94	1.94E-03
SPCC777.04	2.26	3.99E-03	SPCC736.05	1.29	2.29E-04	SPCP1E11.03	0.94	2.20E-05
SPCC330.04c	2.25	2.32E-08	SPAC1F8.05	1.29	2.13E-02	SPBC19C2.11c	0.94	5.95E-03
SPCC1840.12	2.25	8.28E-05	SPACUNK4.10	1.29	1.79E-02	SPBC36.01c	0.94	2.45E-02
SPCC162.10	2.22	3.47E-03	SPAC30D11.02c	1.29	2.17E-02	SPAC1565.03	0.94	3.36E-02
SPBC31F10.08	2.20	1.44E-04	SPAC31G5.10	1.27	1.66E-03	SPAC56F8.14c	0.93	4.31E-02
SPCC338.18	2.19	1.39E-03	SPCC1020.05	1.27	1.86E-03	SPAC3F10.17	0.93	1.31E-03
SPAC1006.04c	2.18	3.18E-06	SPBC21D10.08c	1.27	3.81E-02	SPCC11E10.03	0.92	9.64E-04
SPAC1F7.07c	2.17	7.51E-03	SPAC23G3.02c	1.26	7.51E-05	SPBC83.09c	0.92	1.70E-02
SPAC14C4.01c	2.07	6.99E-05	SPCC338.08	1.26	4.14E-04	SPAC328.08c	0.92	2.26E-02
SPBC1685.05	2.07	5.19E-07	SPBC6B1.05c	1.25	2.02E-05	SPBC12C2.03c	0.92	3.51E-03
SPCC70.04c	2.06	7.02E-05	SPAC22H12.01c	1.25	4.08E-03	SPBC685.03	0.92	1.42E-04
SPBC1778.04	2.04	2.92E-06	SPAC4H3.08	1.25	3.05E-04	SPBC17D1.01	0.92	2.88E-03
SPBC14C8.01c	2.04	7.84E-05	SPBC32H8.11	1.24	7.27E-03	SPCP31B10.06	0.91	3.64E-02
SPAC20H4.11c	2.03	8.15E-04	SPAC27D7.11c	1.23	3.55E-02	SPBC6B1.06c	0.90	1.16E-03
SPBC32C12.02	2.03	1.20E-02	SPBC15D4.01c	1.23	5.60E-03	SPCC737.03c	0.90	2.31E-03
SPCC74.02c	2.02	5.55E-07	SPBC9B6.03	1.23	1.44E-02	SPBC1604.18c	0.90	9.35E-03
SPCPB16A4.06c	2.02	1.50E-04	SPBC1A4.01	1.22	2.57E-03	SPAC17C9.02c	0.90	1.13E-03
SPAC29A4.12c	2.02	9.22E-04	SPAPB1A10.08	1.22	1.47E-04	SPAC56E4.06c	0.90	2.53E-04
SPCC1020.09	1.99	2.30E-03	SPBC19C2.06c	1.22	3.32E-02	SPAC6G9.12	0.89	1.77E-03
SPBC354.08c	1.97	8.65E-05	SPAC4H3.11c	1.21	5.74E-04	SPCC16A11.08	0.89	3.06E-03
SPBC1685.14c	1.96	6.09E-05	SPCC285.09c	1.21	2.04E-03	SPCC162.05	0.89	4.18E-03
SPBC725.10	1.95	1.55E-02	SPAC821.04c	1.20	1.73E-02	SPBP8B7.04	0.89	4.46E-03
SPBC1289.16c	1.92	9.16E-03	SPAC11D3.09	1.20	8.54E-04	SPAC4H3.02c	0.89	3.06E-03
SPAC1F12.10c	1.92	2.92E-02	SPCC1393.13	1.20	1.34E-03	SPCPJ732.02c	0.89	3.35E-03
SPAC4F10.08	1.91	4.96E-07	SPBC651.05c	1.20	7.43E-04	SPAPB24D3.04c	0.89	8.76E-03
SPBC609.04	1.89	8.18E-03	SPBC409.03	1.18	4.28E-02	SPBC8E4.02c	0.88	3.79E-03
SPAC23H3.15c	1.86	3.19E-02	SPAC19G12.01c	1.18	5.96E-07	SPBC660.06	0.88	3.09E-02

SPAC24C9.15c	1.84	7.31E-03	SPBC21C3.11	1.16	1.77E-03	SPCC1442.07c	0.88	4.26E-02
SPAC1610.03c	1.83	3.64E-06	SPBP19A11.07c	1.15	4.44E-05	SPCC338.12	0.88	4.48E-02
SPAC1952.15c	1.83	4.67E-06	SPBC1105.14	1.15	3.40E-02	SPCC1281.04	0.88	1.46E-02
SPBC23G7.11	1.81	6.89E-04	SPAC17A5.04c	1.15	1.74E-06	SPBC3D6.10	0.87	1.49E-02
SPBC16D10.08c	1.80	2.21E-02	SPAC4A8.04	1.15	1.48E-02	SPAC23H4.11c	0.87	3.97E-02
SPMIT.10	1.79	3.82E-05	SPBC14C8.11c	1.15	5.68E-03	SPBP22H7.04	0.87	1.55E-03
SPAC6C3.07	1.79	2.44E-06	SPAC31A2.12	1.15	1.04E-04	SPACUNK4.16c	0.87	3.94E-02
SPBC14C8.05c	1.78	2.72E-07	SPAC17H9.19c	1.14	4.48E-02	SPAC6G9.08	0.87	1.94E-03
SPAC1F8.04c	1.78	1.61E-02	SPBC1E8.05	1.14	8.60E-03	SPCC550.12	0.87	2.86E-02
SPAC22F3.12c	1.77	3.16E-02	SPBC6B1.03c	1.13	7.01E-04	SPAC1805.09c	0.87	1.98E-02
SPAC13C5.03	1.76	9.05E-06	SPCC794.02	1.13	6.53E-03	SPAC10F6.15	0.87	7.47E-03
SPBC1685.12c	1.76	3.02E-03	SPAC227.13c	1.13	9.05E-04	SPAC631.02	0.86	2.33E-02
SPCC4G3.03	1.75	4.36E-05	SPBC1683.12	1.13	4.88E-02	SPCC417.11c	0.86	2.76E-02
SPAC3F10.10c	1.74	7.67E-07	SPCC548.04	1.12	2.73E-03	SPAC4F10.09c	0.86	1.08E-02
SPCC584.02	1.73	1.85E-05	SPCC1739.15	1.12	8.50E-04	SPBC16E9.08	0.86	2.15E-03
SPAC25H1.09	1.72	2.27E-05	SPBC18H10.09	1.12	5.21E-05	SPBC26H8.08c	0.86	4.12E-02
SPCC1919.14c	1.72	4.18E-05	SPAC6C3.05	1.12	2.08E-03	SPBC16D10.07c	0.85	1.81E-05
SPBC146.11c	1.71	3.61E-06	SPBC1105.17	1.12	1.71E-03	SPBC16A3.17c	0.85	3.80E-02
SPCC830.04c	1.71	2.58E-04	SPAC644.14c	1.12	1.04E-04	SPCC63.06	0.85	8.88E-04
SPBC27.03	1.71	2.12E-06	SPBC19G7.13	1.11	1.60E-04	SPAC328.03	0.85	3.05E-02
SPBC3H7.08c	1.69	1.40E-05	SPAC22E12.03c	1.11	2.59E-03	SPAC56F8.13	0.85	6.71E-04
SPAC869.02c	1.69	3.83E-03	SPAC17G6.13	1.10	2.19E-02	SPCC830.03	0.85	2.44E-03
SPBC21C3.02c	1.68	6.85E-06	SPCC18.10	1.10	1.63E-05	SPAC23D3.01	0.84	7.34E-04
SPBC3D6.11c	1.66	2.48E-06	SPCC965.05c	1.09	5.91E-05	SPAC15A10.04c	0.84	3.61E-02
SPAC18G6.09c	1.66	8.05E-03	SPAC8C9.12c	1.09	6.22E-04	SPAC8C9.11	0.84	2.94E-02
SPAC2E1P3.02c	1.64	1.28E-04	SPAC926.08c	1.08	3.51E-02	SPAC328.04	0.83	4.68E-04
SPCPB1C11.02	1.62	1.31E-03	SPAC25G10.04c	1.08	7.18E-03	SPAC1250.02	0.83	1.62E-02
SPBC530.11c	1.61	4.67E-04	SPCC1223.12c	1.08	1.04E-02	SPAC24B11.06c	0.83	7.40E-04
SPBC4F6.09	1.61	2.36E-02	SPAC20G4.03c	1.08	3.75E-02	SPAC13D6.02c	0.83	3.94E-03
SPAC1A6.11	1.60	5.63E-06	SPBC1711.11	1.08	1.41E-03	SPBC3F6.04c	0.82	3.46E-02
SPAC4H3.04c	1.60	1.88E-03	SPAC1250.01	1.08	5.08E-04	SPCC61.01c	0.82	8.37E-03

SPAC5H10.07	1.58	8.84E-05	SPCC4F11.02	1.08	7.99E-03	SPAC56F8.16	0.82	1.56E-02
SPAC22A12.02c	1.56	7.62E-06	SPBC337.11	1.08	8.06E-06	SPBC18H10.21c	0.82	5.14E-03
SPAC6G9.01c	1.54	1.12E-03	SPAC1565.04c	1.07	3.04E-02	SPAC1002.12c	0.82	1.95E-02
SPAPB1A11.03	1.52	1.40E-05	SPCC1322.03	1.06	1.38E-02	SPAPB2B4.06	0.81	4.54E-03
SPCC1223.02	1.52	1.04E-04	SPAC19B12.08	1.06	3.93E-02	SPCC132.03	0.81	1.40E-02
SPBC18E5.10	1.52	3.93E-05	SPAC15F9.01c	1.06	7.42E-03	SPCC70.09c	0.81	1.06E-02
SPCC970.11c	1.51	1.10E-03	SPAC186.01	1.05	1.34E-02	SPAC6B12.08	0.81	9.73E-03
SPCC965.08c	1.51	5.89E-08	SPBC8D2.19	1.04	1.55E-03	SPAC13G7.07	0.81	2.00E-02
SPBC1718.02	1.51	1.62E-04	SPCC126.02c	1.04	4.81E-04	SPCC584.15c	0.81	1.29E-02
SPAC22G7.11c	1.50	4.45E-04	SPAC589.08c	1.04	2.30E-02	SPAC1B3.15c	0.81	1.70E-02
SPAC16E8.05c	1.49	5.39E-05	SPAC1F7.05	1.04	4.25E-02	SPAC688.03c	0.80	1.92E-02
SPAC1556.06b	1.49	3.37E-03	SPCC1020.13c	1.04	1.35E-03	SPAC1527.03	0.80	1.23E-02
SPAC27D7.05c	1.49	1.14E-03	SPBC354.06	1.04	3.10E-03	SPBC800.14c	0.80	3.46E-02
SPBC32H8.07	1.48	7.42E-05	SPAC4D7.10c	1.04	1.91E-03	SPCC16A11.15c	0.80	1.54E-02
SPAC343.07	1.48	1.58E-04	SPCC736.15	1.04	3.12E-02	SPAC13G6.05c	0.80	5.77E-03
			SPBC16A3.02c	1.03	2.41E-03	SPBC27B12.03c	0.80	1.05E-03

mst1^{ts}

Gene	Log2 Change	pvalue	Gene	Log2 Change	pvalue	Gene	Log2 Change	pvalue
SPAC869.09	6.68	2.91E-12	SPAC22F8.05	1.90	1.56E-03	SPBC56F2.05c	1.13	2.88E-07
SPAC869.06c	6.61	2.79E-13	SPBC1348.09	1.89	3.69E-11	SPCC830.04c	1.13	4.93E-04
SPAC1F8.05	5.36	1.87E-11	SPAC14C4.01c	1.87	3.69E-06	SPAC458.04c	1.13	9.70E-04
SPBC23G7.10c	5.25	4.94E-06	SPAC27E2.04c	1.87	1.98E-08	SPCC417.04	1.13	5.53E-07
SPAC22G7.11c	5.22	4.34E-14	SPCC576.17c	1.87	2.59E-08	SPAC1565.04c	1.12	2.73E-03
SPAC3G9.11c	5.13	5.88E-11	SPAC11D3.13	1.86	2.69E-03	SPBC1271.05c	1.12	6.81E-04
SPBPB21E7.01c	5.11	3.01E-13	SPAC186.08c	1.85	7.53E-13	SPBC14C8.01c	1.11	1.01E-03
SPAC186.02c	5.11	6.43E-15	SPAC13F5.03c	1.85	2.29E-04	SPCC338.08	1.08	5.52E-05
SPAC977.16c	5.02	4.45E-12	SPAC977.03	1.84	7.01E-08	SPAC4D7.02c	1.08	1.01E-03
SPBC24C6.09c	4.94	1.55E-13	SPAC750.04c	1.84	2.17E-06	SPBC19G7.06	1.07	6.95E-07
SPAC22H10.13	4.85	1.83E-08	SPAC6B12.03c	1.84	1.97E-03	SPAC15A10.05c	1.07	4.43E-03
SPBCPT2R1.08c	4.76	1.05E-04	SPBC1604.01	1.81	4.55E-10	SPAC10F6.15	1.07	5.54E-05

SPAC1F8.01	4.72	1.97E-03	SPAC26F1.04c	1.81	1.42E-07	SPAC57A10.05c	1.07	9.94E-05
SPBC1289.14	4.61	8.12E-16	SPBC725.03	1.80	2.78E-04	SPBC359.02	1.06	3.40E-05
SPCPB16A4.06c	4.53	1.21E-11	SPAC1002.20	1.78	1.18E-07	SPCC1682.11c	1.06	4.16E-05
SPAC139.05	4.49	1.53E-09	SPBC1685.12c	1.78	1.13E-04	SPAC57A7.05	1.06	2.98E-02
SPAC869.07c	4.43	2.62E-15	SPBC887.16	1.78	9.01E-05	SPAC25H1.09	1.05	1.16E-04
SPBC56F2.06	4.36	2.99E-07	SPAC8E11.03c	1.76	2.30E-05	SPAC3A11.10c	1.05	1.37E-03
SPBC1198.14c	4.32	3.57E-14	SPBCPT2R1.02	1.75	2.46E-06	SPAP7G5.03	1.05	3.04E-06
SPBC3H7.08c	4.09	9.33E-14	SPBC19C2.06c	1.74	1.97E-04	SPAC16E8.03	1.05	4.37E-04
SPAC869.08	4.08	1.01E-15	SPAC3C7.05c	1.73	1.30E-07	SPBC32F12.09	1.05	5.21E-05
SPAC29A4.12c	4.06	1.45E-09	SPCC1235.13	1.72	9.81E-09	SPAC19D5.06c	1.05	1.26E-08
SPBC16E9.16c	4.00	6.07E-13	SPBC1289.16c	1.69	1.86E-03	SPAPB1E7.08c	1.04	2.41E-03
SPAPB8E5.10	3.98	1.31E-12	SPAC1F7.08	1.68	9.05E-03	SPCC584.12	1.04	1.37E-03
SPBC1A4.01	3.96	4.49E-12	SPAC1F7.07c	1.67	4.16E-03	SPAC26F1.10c	1.03	2.03E-03
SPBC359.06	3.91	5.00E-04	SPAC16A10.01	1.67	5.76E-07	SPCC4G3.12c	1.03	4.78E-06
SPCC737.04	3.85	3.10E-07	SPAC750.07c	1.67	5.11E-04	SPAC1751.01c	1.03	3.57E-02
SPBC1683.09c	3.69	3.37E-05	SPAC977.07c	1.65	9.39E-07	SPCC550.10	1.02	3.46E-06
SPBPB21E7.04c	3.67	3.08E-06	SPAC26F1.14c	1.63	1.76E-04	SPAC5D6.10c	1.02	4.49E-06
SPCC663.06c	3.66	3.28E-05	SPBC1289.15	1.62	2.28E-04	SPAC56F8.13	1.02	1.65E-06
SPBP4G3.03	3.65	2.74E-13	SPAPB1A10.14	1.61	5.00E-05	SPAC9.08c	1.02	2.89E-02
SPBPB8B6.03	3.64	2.07E-12	SPBPB2B2.11	1.60	3.89E-06	SPBC9B6.03	1.02	5.43E-03
SPAC11D3.01c	3.61	1.72E-07	SPBPB2B2.01	1.59	2.51E-03	SPBC2F12.15c	1.01	1.47E-04
SPAC1F8.04c	3.59	5.41E-07	SPBC3E7.02c	1.59	3.78E-03	SPCC417.02	1.01	2.72E-04
SPAPB1A11.03	3.59	1.50E-13	SPAC22G7.08	1.59	1.64E-08	SPBC115.03	1.01	1.55E-02
SPAC22A12.17c	3.57	1.96E-11	SPAC13F5.07c	1.57	2.96E-04	SPAC1006.01	1.01	2.69E-05
SPBC1198.01	3.55	7.19E-16	SPBC354.11c	1.57	6.76E-06	SPAC11G7.05c	1.01	1.66E-07
SPAPJ695.01c	3.52	2.00E-09	SPAC3C7.02c	1.56	1.32E-06	SPAC29B12.12	1.01	8.96E-04
SPCC1795.06	3.52	2.50E-12	SPBC19C2.05	1.55	1.41E-03	SPAC27D7.11c	1.00	1.78E-02
SPAC1F8.03c	3.48	2.47E-05	SPCC417.10	1.55	1.01E-05	SPAC6C3.05	1.00	2.58E-04
SPBC609.04	3.44	4.99E-07	SPAC6G10.03c	1.53	3.38E-06	SPBC1198.07c	1.00	2.47E-04
SPAC23H3.15c	3.42	1.03E-05	SPAC977.06	1.52	4.06E-09	SPCC1739.10	1.00	1.24E-03
SPACUNK4.17	3.33	3.64E-07	SPBC4F6.09	1.52	4.04E-03	SPAC4F10.07c	1.00	3.28E-07

SPAC3G6.07	3.33	1.53E-05	SPAP11E10.02c	1.51	9.62E-04	SPAC1F7.10	0.98	6.97E-04
SPAC1F8.08	3.32	2.20E-09	SPCC320.07c	1.50	3.94E-08	SPBC1778.04	0.98	2.40E-04
SPAC977.05c	3.29	2.46E-05	SPAC13A11.06	1.50	2.85E-05	SPAC22E12.03c	0.98	3.60E-04
SPAC27D7.03c	3.25	8.89E-04	SPAC27D7.09c	1.49	1.26E-02	SPAPB24D3.02c	0.98	5.43E-07
SPAC31G5.09c	3.17	2.17E-04	SPAC27F1.05c	1.49	6.79E-06	SPBC23G7.08c	0.98	4.93E-06
SPBPB2B2.12c	3.16	7.06E-03	SPCC965.06	1.48	8.33E-05	SPAC5D6.04	0.98	1.70E-05
SPAC4H3.08	3.16	4.98E-12	SPAC31G5.10	1.48	9.73E-06	SPAC2F7.06c	0.97	2.29E-02
SPCC757.03c	3.11	2.10E-09	SPCC188.09c	1.48	1.31E-09	SPBC800.14c	0.97	1.02E-03
SPCC1322.07c	3.10	9.51E-09	SPBC31F10.08	1.47	2.58E-04	SPCC1281.07c	0.97	7.95E-03
SPBPB2B2.18	3.05	1.46E-05	SPBC215.11c	1.47	4.82E-03	SPCC338.12	0.96	3.69E-03
SPCC338.18	3.04	7.26E-07	SPBC1683.01	1.47	2.17E-06	SPAC26H5.09c	0.96	1.85E-02
SPBPB8B6.04c	3.04	7.61E-08	SPAC23E2.03c	1.47	1.47E-02	SPAC22F8.02c	0.96	3.73E-05
SPBC1105.13c	3.02	1.10E-07	SPBC19F8.06c	1.45	1.04E-06	SPAPB1A10.13	0.96	2.42E-03
SPAC15E1.02c	3.00	8.68E-09	SPCP31B10.06	1.45	7.41E-05	SPBC3H7.06c	0.95	6.77E-03
SPAC23C11.06c	2.99	1.15E-07	SPAC22G7.07c	1.44	2.63E-07	SPCC31H12.06	0.95	4.50E-05
SPCC1739.08c	2.96	2.15E-02	SPBC1652.01	1.43	5.97E-05	SPBC1E8.05	0.94	2.77E-03
SPCC16A11.15c	2.94	5.09E-11	SPBC1105.14	1.43	8.01E-04	SPCC736.13	0.94	9.33E-05
SPAC4F10.17	2.88	6.57E-09	SPAC29A4.17c	1.42	3.30E-04	SPBC21C3.10c	0.94	2.41E-02
SPBC1685.05	2.86	1.62E-11	SPCC777.03c	1.42	1.75E-04	SPCC1183.09c	0.94	1.08E-03
SPBC1773.06c	2.76	4.69E-04	SPBC2F12.09c	1.42	1.58E-10	SPAC29E6.01	0.94	8.52E-06
SPAPJ691.02	2.72	1.52E-05	SPCC4G3.03	1.42	8.84E-06	SPAC3G9.08	0.94	2.44E-04
SPAC56F8.15	2.68	1.28E-03	SPAC56F8.14c	1.41	1.86E-04	SPCC417.11c	0.94	1.74E-03
SPBC2G2.17c	2.66	2.11E-07	SPBC36B7.06c	1.41	1.26E-04	SPAC1556.06b	0.93	7.79E-03
SPAC32A11.02c	2.65	4.69E-09	SPAC19G12.09	1.40	2.69E-05	SPBC146.11c	0.93	8.17E-05
SPCC1020.01c	2.64	6.19E-07	SPAC1A6.06c	1.39	1.65E-05	SPCC757.13	0.92	1.20E-05
SPAC26F1.05	2.64	1.00E-07	SPAC2E1P3.01	1.39	8.75E-05	SPAC4G8.04	0.92	3.46E-06
SPCC777.04	2.63	3.77E-05	SPBC21.07c	1.38	5.91E-08	SPAP27G11.12	0.92	3.35E-06
SPBPB2B2.08	2.60	7.87E-03	SPAC3F10.10c	1.37	1.64E-07	SPCC663.03	0.92	1.04E-03
SPCC1393.12	2.58	4.35E-11	SPAPB18E9.04c	1.37	3.66E-05	SPBC12C2.14c	0.92	3.64E-07
SPBC83.19c	2.58	3.49E-12	SPAC20H4.11c	1.37	1.26E-03	SPBC16E9.13	0.92	1.89E-07
SPBC725.10	2.57	9.52E-05	SPCPJ732.03	1.36	1.74E-05	SPCC1223.02	0.91	5.23E-04

SPCC663.08c	2.54	9.81E-03	SPAC343.12	1.32	8.35E-03	SPBC2D10.04	0.91	8.49E-06
SPAC5H10.02c	2.46	3.89E-11	SPCC794.03	1.32	2.22E-04	SPCC757.02c	0.91	5.53E-04
SPCC1906.04	2.44	5.08E-06	SPBC1D7.02c	1.32	1.30E-03	SPAC57A7.09	0.91	3.86E-03
SPBC1348.14c	2.41	2.14E-15	SPBC660.09	1.31	4.89E-08	SPAC25B8.19c	0.90	5.75E-06
SPAC977.17	2.40	1.80E-08	SPBC11C11.06c	1.30	2.04E-02	SPCC1529.01	0.90	2.94E-02
SPAPB24D3.10c	2.37	2.63E-02	SPBC660.06	1.30	1.25E-04	SPAC23C11.07	0.90	2.19E-04
SPBC839.06	2.36	6.88E-11	SPBC947.09	1.29	3.43E-07	SPAC14C4.08	0.89	3.44E-02
SPAC13G7.02c	2.34	8.34E-04	SPBC1773.05c	1.29	1.35E-05	SPBC19C7.03	0.88	1.47E-03
SPAC1F7.06	2.33	5.44E-12	SPCC1183.11	1.29	6.12E-05	SPCC550.07	0.88	7.70E-03
SPCC162.10	2.28	1.19E-04	SPAC30D11.02c	1.28	2.33E-03	SPBC2G2.10c	0.88	2.21E-06
SPBC32C12.02	2.27	3.35E-04	SPBC16A3.02c	1.28	7.49E-06	SPAC4G9.07	0.87	3.14E-03
SPCPB1C11.02	2.25	7.04E-07	SPCC1442.07c	1.28	2.97E-04	SPBC409.07c	0.87	1.11E-06
SPAC513.06c	2.24	1.52E-09	SPCC830.05c	1.27	3.16E-09	SPCC330.04c	0.87	7.08E-05
SPCC1393.07c	2.23	4.88E-10	SPAPB2B4.04c	1.27	5.22E-05	SPAC11E3.13c	0.87	4.61E-06
SPBC36.02c	2.23	1.36E-04	SPAC4A8.04	1.26	5.82E-04	SPAC29A4.13	0.87	1.42E-04
SPBPB8B6.02c	2.23	1.43E-12	SPBP4H10.10	1.25	3.33E-02	SPBC19C7.05	0.86	1.49E-02
SPBC4.01	2.20	9.35E-05	SPBC21B10.12	1.25	2.42E-08	SPAPB1A11.01	0.86	2.50E-03
SPAC1F8.02c	2.18	3.57E-07	SPCC1281.08	1.25	1.62E-07	SPBC354.08c	0.86	6.08E-03
SPCC794.01c	2.18	4.23E-02	SPBC106.02c	1.25	8.64E-03	SPBC8D2.20c	0.86	1.96E-03
SPAPB15E9.02c	2.17	3.00E-08	SPBC660.07	1.24	4.93E-04	SPCC794.02	0.85	4.13E-03
SPAC4H3.03c	2.14	4.86E-04	SPAC637.12c	1.23	1.21E-06	SPAC18G6.09c	0.85	4.39E-02
SPAC513.02	2.12	1.94E-06	SPAC11E3.14	1.22	1.11E-06	SPAC23A1.06c	0.85	2.28E-04
SPBC365.12c	2.11	9.95E-06	SPBC146.02	1.21	2.11E-04	SPBC8D2.19	0.85	4.47E-04
SPAC3H8.09c	2.11	1.77E-10	SPBC609.01	1.21	2.27E-09	SPBP19A11.07c	0.85	2.98E-05
SPBC947.05c	2.11	5.62E-08	SPAC1F7.12	1.21	6.28E-07	SPAC20G4.05c	0.84	7.65E-05
SPAC5H10.04	2.10	1.33E-06	SPCC126.07c	1.20	3.53E-08	SPBC1A4.02c	0.84	5.93E-06
SPBC19C7.04c	2.09	1.28E-03	SPACUNK4.19	1.20	1.53E-05	SPBC29A3.08	0.84	4.84E-03
SPBPB2B2.02	2.06	3.06E-04	SPAC19B12.08	1.19	2.44E-03	SPAC20G4.03c	0.83	2.51E-02
SPCC1840.12	2.05	4.17E-06	SPAC17A2.01	1.19	2.40E-07	SPBC16E9.11c	0.83	6.41E-06
SPCC1223.12c	2.05	4.69E-07	SPBC405.02c	1.18	7.98E-05	SPAC328.03	0.83	4.68E-03
SPBC4C3.08	2.04	6.23E-08	SPBC354.12	1.18	7.96E-05	SPCC736.11	0.83	6.80E-06

SPAC637.03	2.03	1.33E-04	SPAC167.06c	1.18	5.55E-03	SPCC285.16c	0.83	6.73E-06
SPBC8E4.05c	1.99	1.54E-08	SPBC685.03	1.16	8.69E-08	SPBC146.06c	0.82	5.62E-04
SPAC11E3.06	1.98	6.19E-04	SPAC1786.04	1.16	3.48E-05	SPAC688.04c	0.82	5.52E-04
SPAC9.10	1.98	8.57E-09	SPAC26H5.08c	1.16	1.50E-04	SPAC2C4.17c	0.82	1.01E-03
SPBC16A3.13	1.96	2.81E-05	SPCC1322.08	1.15	2.57E-03	SPAC186.09	0.82	5.01E-08
SPCC70.04c	1.95	2.09E-06	SPAP8A3.04c	1.15	3.11E-02	SPCC417.05c	0.81	3.23E-03
SPCC191.01	1.94	5.61E-06	SPCC576.01c	1.15	1.01E-03	SPCC364.02c	0.81	3.45E-04
SPBC23E6.03c	1.94	3.20E-06	SPBC15D4.02	1.14	1.67E-03	SPAC1610.03c	0.81	6.80E-04
SPBPB2B2.07c	1.93	9.41E-10	SPBC12C2.03c	1.14	1.51E-05	SPAC343.07	0.80	1.77E-03
SPBPB2B2.06c	1.93	9.87E-04	SPAC3A12.06c	1.14	2.93E-07	SPBP8B7.30c	0.80	3.90E-05
SPAC3C7.13c	1.92	3.23E-10	SPBC32H8.13c	1.14	6.14E-06	SPCC737.03c	0.80	3.26E-04
			SPBC24C6.06	1.13	2.19E-04	SPBC21H7.06c	0.80	2.18E-02
